# Non-fermented and fermented milk intake in relation to risk of ischemic heart disease and to circulating cardiometabolic proteins in swedish women and men: Two prospective longitudinal cohort studies with 100,775 participants

**DOI:** 10.1186/s12916-024-03651-1

**Published:** 2024-11-08

**Authors:** Karl Michaëlsson, Eva Warensjö Lemming, Susanna C. Larsson, Jonas Höijer, Håkan Melhus, Bodil Svennblad, John A. Baron, Alicja Wolk, Liisa Byberg

**Affiliations:** 1 Medical Epidemiology, Department of Surgical Sciences, Uppsala University, Uppsala, Sweden; 2 Unit of Cardiovascular and Nutritional Epidemiology, Institute of Environmental Medicine, Karolinska Institutet, Stockholm, Sweden; 3 Clinical Pharmacology, Department of Medical Sciences, Uppsala University, Uppsala, Sweden; 4 Department of Epidemiology, Gillings School of Global Public Health, University of North Carolina, Chapel Hill, NC USA

**Keywords:** Milk, Fermented, Non-fermented, Cohort, Myocardial infarction, Ischemic heart disease, Cohort, ACE2, FGF21

## Abstract

**Background:**

The effect of milk on the risk of ischemic heart disease (IHD) and acute myocardial infarction (MI) is unclear. We aimed to examine the association between non-fermented and fermented milk consumption on these endpoints and investigate the relationship between milk intake and cardiometabolic-related proteins in plasma.

**Methods:**

Our study is based on two Swedish prospective cohort studies that included 59,998 women and 40,777 men without IHD or cancer at baseline who provided repeated measures of diet and lifestyle factors and plasma proteomics data in two subcohorts. Through registry linkage, 17,896 cases with IHD were documented during up to 33 years of follow-up, including 10,714 with MI. We used time-updated multivariable Cox regression analysis to examine non-fermented or fermented milk intake with time to IHD or MI. Using high-throughput multiplex immunoassays, 276 cardiometabolic plasma proteins were measured in two subcohorts. We applied multivariable-adjusted regression models using a discovery-replication design to examine protein associations with increasing consumption of non-fermented or fermented milk.

**Results:**

The results for non-fermented milk differed by sex (*p*-value for interaction = 0.01). In women, we found a pattern of successively greater risk of IHD and MI at non-fermented milk intake levels higher than 1.5 glasses/day. Compared with an intake of 0.5 glass/day (100 mL/day), non-fermented milk intake of 2 glasses/day in women conferred a multivariable-adjusted hazard ratio (HR) of 1.05 (95% CI 1.01–1.08) for IHD, an intake of 3 glasses/day an HR of 1.12 (95% CI 1.06–1.19), and an intake of 4 glasses/day an HR of 1.21 (95% CI 1.10–1.32). Findings were similar for whole, medium-fat, and low-fat milk. We did not detect higher risks of IHD with increasing milk intakes in men. Fermented milk intake was unrelated to the risk of IHD or MI in either sex. Increasing non-fermented milk intake in women was robustly associated with a higher concentration of plasma ACE2 and a lower concentration of FGF21.

**Conclusions:**

We show a positive association between high amounts of non-fermented milk intake and IHD in women but not men. We suggest metabolic pathways related to ACE2 and FGF21 potentially underlie the association.

**Graphical abstract:**

Our analysis of two large cohort studies involving 100,775 participants and 17,896 clinically confirmed IHD events supports a dose–response positive association between non-fermented milk intake higher than 300 mL/day with higher rates of IHD (and acute MI specifically) in women, but not in men. The higher risk of IHD with high milk intake in women was evident, irrespective of the fat content of the milk. Fermented milk intake was unrelated to the risk of IHD in both women and men. Non-fermented milk intake was associated in different directions with circulating levels of ACE2 and FGF21 in women—two essential cardiometabolic proteins, also related to IHD in women in our study.

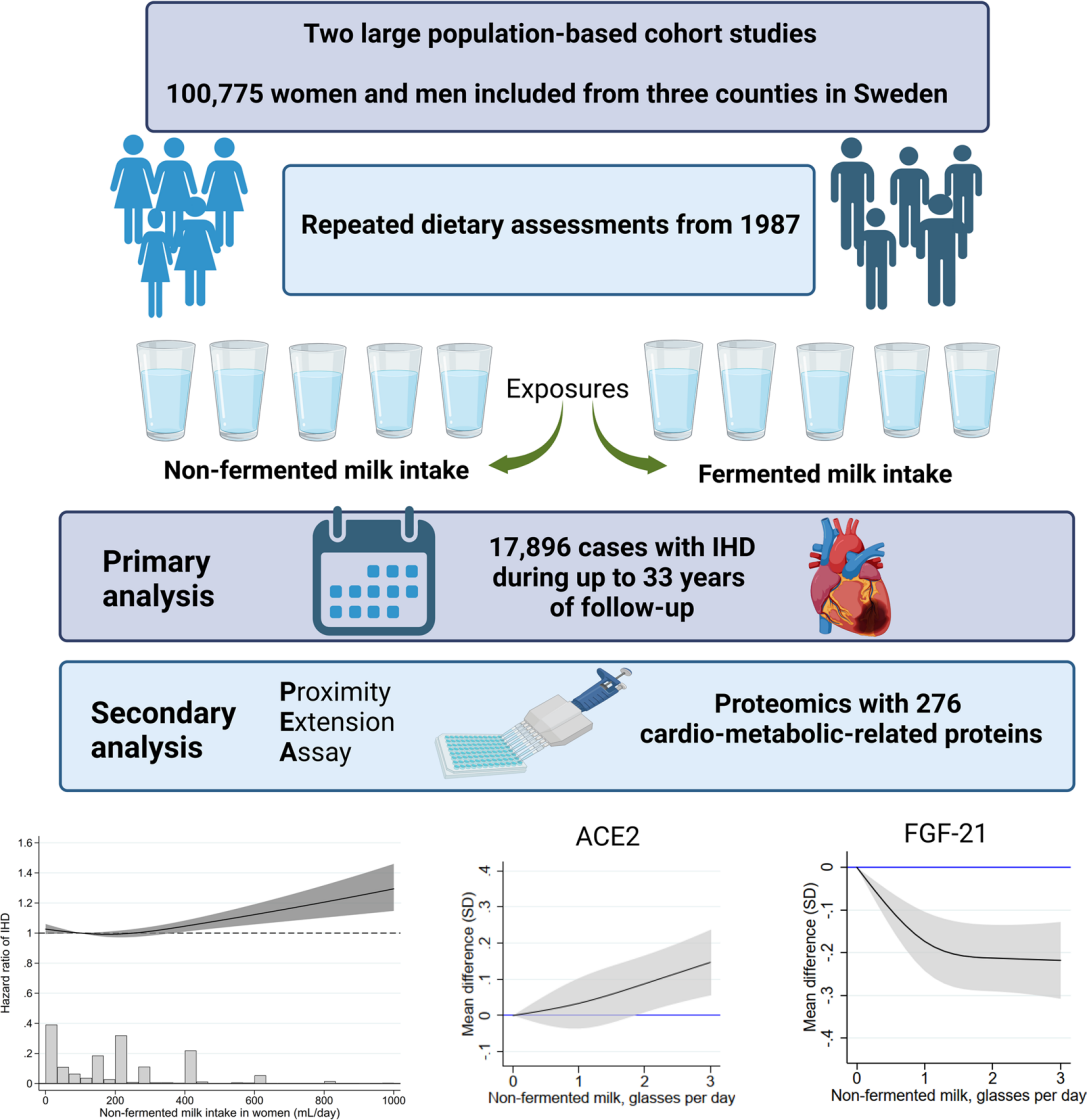

**Supplementary Information:**

The online version contains supplementary material available at 10.1186/s12916-024-03651-1.

## Background

Ischemic heart disease (IHD), a consequence of genetic and environmental influences [1], is globally the leading cause of years of life lost [2, 3]. It is recognized that a healthy diet is essential for the prevention of cardiovascular diseases [4]. However, there is uncertainty about the effect of milk products on the risk of IHD: results from cohort studies have been conflicting [5–8], and further research on the topic has been recommended [6]. Because of potential non-linear associations, including individuals with low and pronounced high milk intakes is essential. However, few studies have sufficient individuals with high milk consumption [5–9]. Non-fermented and fermented milk is widely consumed, especially in Scandinavian countries, but may affect cardiovascular health differently [5, 6, 10–14]. In addition, consumption patterns of different dairy products have changed considerably over the last half-century, with, on average, lower intake of non-fermented milk combined with increases in fermented milk in many settings. The study design should ideally capture these changes.

Due to the uncertainty regarding the impact of milk intake on IHD and other outcomes, recommendations regarding the type and amount of milk products vary. In the United States, the recommended intake of non-fermented milk or equivalent portions of fermented milk products is three 8-oz (237 mL) daily adult servings. European recommendations are more moderate (300–500 mL/day), emphasizing low-fat milk products.

We used data from two large population-based Swedish longitudinal cohorts of women and men to assess the risk of IHD and myocardial infarction (MI) with a wide range of non-fermented and fermented milk consumption patterns, including milk with different fat contents. We further examined non-fermented and fermented milk consumption with patterns of cardiometabolic plasma protein concentrations.

## Methods

### Study population

The study population consisted of participants from two population-based cohort studies in Sweden: the Swedish Mammography Cohort (SMC) and the Cohort of Swedish Men (COSM), part of the national research infrastructure SIMPLER (www.simpler4health.se) [11, 15]. SMC was established in 1987–1990 when all women born during1914–1948 (*n* = 90,303) and residing in two counties (Uppsala and Västmanland) were invited to participate in a questionnaire survey covering diet and lifestyle. This was completed by 66,432 (74%) of the women. In the fall of 1997, a second extended questionnaire was sent to all SMC participants still alive and residing in the study area (*n* = 56,030). This included almost 350 items that covered diet (a validated semi-quantitative food frequency questionnaire, FFQ), lifestyle factors, body weight and height, dietary supplement use, alcohol consumption, smoking, physical activity, socio-demographic data, self-perceived health status, and family history of MI before the age of 60. The questionnaire was completed by 39,227 (70%) of the women. COSM was established in late 1997 when all male residents (*n* = 100,303) of two counties (Örebro and Västmanland) born between 1918 and 1952 were sent a questionnaire similar to that used in SMC in 1997. This was completed by 48,850 (49%) of the men.

In 2008, a questionnaire covering general health, lifestyle, and diseases was sent to all SMC and COSM participants who had completed the 1997 questionnaire and lived in the study areas. The response rate was 63% in SMC and 78% in COSM. Those who responded to the 2008 questionnaire received an expanded FFQ in 2009; the response rate was 84% and 90% in SMC and COSM, respectively. Compared with the official Statistics of Sweden data, the cohorts represented the Swedish population well in terms of age distribution, educational level, the prevalence of overweight and obesity, and smoking status [16]. Participants with prior IHD (see below for definition), cancer diagnosis, or energy intakes deemed implausible (± 3 SD from the mean of log-transformed energy intake) were excluded, and 59,988 women in the SMC and 40,777 men in the COSM remained for the present analysis.

This study is reported per the Strengthening the Reporting of Observational Studies in Epidemiology (STROBE) guidelines. The study has ethical approvals from the Ethical Review Boards in Uppsala and Stockholm, Sweden, and the participants provided written informed consent. An analysis *plan* can be found in the Additional file 1: Analysis plan.

### Dietary information

The participants reported their average frequency of consumption of up to 132 foods and beverages during the past year [11, 15, 17, 18], i.e., how many servings per day or week they consumed of everyday foods. The FFQ included information about non-fermented milk (3%, 1.5%, or 0.5% fat separately) and fermented milk (soured milk and yogurt but not fermented cream or cheese products). The stated serving size of milk was 200 ml. In the first FFQ (1987, women only), the frequency options for milk consumption were pre-specified, while in the second and the third questionnaires (FFQ 1997 and FFQ 2009—women and men), participants could fill in the exact number of servings of the dairy products they consumed per day or week. Milk intake was specified according to fat content, and intake was summed into a single measure representing total milk intake on a continuous scale and within three milk categories of fat content (< 0.5%, 1.5%, and 3%). Food and nutrient intakes were estimated by multiplying each food item’s reported frequency of consumption by age-specific portion sizes and by the nutrient content of specific foods obtained from the Swedish Food Agency food database [11, 15]. Using these data, we computed total energy intake, coffee, cheese, non-fermented milk and fermented milk, fruit and vegetables, red meat, soft drinks and juice, total fat, and saturated fat.

According to validation studies of milk intake, the correlation between the FFQ and four 7-day food records every third month over a year, a gold standard reference, was approximately 0.7 [19]. Furthermore, in women and men, we have found a positive association between the reported milk intake and the fat tissue content of pentadecanoic acid, a biological marker reflecting the average long-term intake of milk fat from non-fermented and fermented milk products [20, 21].

### Ascertainment of IHD and MI

Incident IHD (any ischemic heart disease event, including angina pectoris and myocardial infarction) and acute myocardial infarction (MI) were our primary outcomes, with information obtained from the continuously updated National Patient Registry and the Cause of Death Registry. A complete linkage with the registries is possible since all Swedish residents have a unique personal identity number. We used the main diagnosis to define our primary outcomes; the ICD9 and ICD10 version codes 410–414 and I20-I25 defined IHD (including angina pectoris, myocardial infarction, and other ischemic heart diseases). The validity, in terms of specificity, of primary diagnoses of MI in the Swedish Patient Register is 98–100% [22]. The accuracy of the classification of causes of death in the Cause of Death Registry is also high [23].

### Assessment of covariates and effect modifiers

Covariates obtained from the questionnaires were age, body mass index (BMI, kg/m^2^), smoking status, walking/cycling, use of vitamin and mineral supplements, alcohol consumption, hypertension, hypercholesterolemia, intakes of fruits and vegetables, intakes of processed meat, intake of soft drink and juice, leisure time physical activity during the past year, and, as indicators of socioeconomic status, educational level and cohabiting/marital status. The physical activity questions have been validated against activity records and accelerometer data [24]. Comorbidity, expressed as Charlson’s weighted comorbidity index [25], was defined using International Classification of Diseases (ICD) diagnosis codes (ICD versions 7, 8, 9, and 10) from the National Patient Registry from 1964 to before baseline. We retrieved information on diabetes mellitus from the National Patient Registry and the National Diabetes Register as well as from the questionnaire self-reports.

### Assessment of plasma proteomics in two subcohorts

We used two sub-cohorts (*n* = 12,406) of SMC and COSM for proteomic analyses, randomly selected from the parent cohorts. The discovery sub-cohort sample for the present study comprised 5022 women who participated in a health examination in Uppsala between 2003 and 2009. The replication sub-cohort included 7384 individuals (2760 women and 4754 men) participating in a health examination in Västerås, Västmanland County, between 2010 and 2019. Blood samples in both sub-cohorts were collected in the morning following an overnight fast. For plasma preparation, blood samples were light-protected. After a 15–20-min delay at room temperature, samples were spun at 1615 g for 11 min at 4 °C. The plasma was frozen in aliquots and stored at – 80 °C until analysis. Analysis of 276 protein biomarkers was accomplished using three high-throughput, multiplex immunoassays: the Olink Proseek Multiplex CVD 2, CVD 3, and Metabolism panels (Olink Bioscience, Uppsala, Sweden). The proteomic profiling was done at the Clinical Biomarkers Facility, SciLifeLab, Uppsala, Sweden. All protein names and abbreviations are found in Additional file 1: Table. S1. Each panel enables simultaneous analysis of 92 protein biomarkers. Each assay uses a proximity extension assay (PEA) technology, in which 92 oligonucleotide-labeled antibody probe pairs are allowed to bind to their target present in the sample. PEA uses pairs of antibodies equipped with DNA reporter molecules, producing DNA amplicons, which are subsequently quantified using a Fluidigm BioMark HD real-time polymerase chain reaction platform. The method has acceptable reproducibility and repeatability, with mean intra-assay and interassay coefficients of variation of < 8% and < 12%, respectively. Because only correctly matched antibody pairs produce a signal, the PEA technique has an accuracy advantage over conventional multiplex immunoassays. The relative concentration of the proteins in plasma was measured, which provides normalized protein expression values on a log2 scale standardized per analysis plate. Olink NPX Manager software was applied for data analysis, and a one-unit higher NPX value represents a doubling of measured protein concentration. In this study, we also use plasma concentrations expressed as standard deviation differences as our outcome. Participants in the sub-cohorts had responded to lifestyle, food frequency, and health questionnaires in 1987–1990 (only women), 1997, 2008–2009, and additionally 1 month before the clinical examination.

### Statistical analyses

We calculated the time at risk for IHD and MI for each participant from baseline (1987–1990 for women and 1998 for men) until the date of the first outcome diagnosis, date of death, or end of follow-up on December 31, 2021, whichever came first. We excluded individuals with a history of IHD or cancer before baseline. When MI was the outcome, we censored for other types of IHD at the first date of those diagnoses. Analyses were performed among both sexes and women and men separately. A sex interaction analysis used 1998 as the baseline for both women and men. In the primary analyses, we used time-updated information on exposure and covariates from the following investigations: 1987–1990 (women only), 1997, and 2008/2009.

The associations of non-fermented and fermented milk with incident IHD and MI were assessed as age-adjusted and multivariable-adjusted hazard ratios (HR) with 95% confidence intervals (CI) by Cox proportional hazards regression models with time-updated information of all variables except education, diabetes mellitus, major cardiovascular disease other than IHD, and Charlson’s comorbidity index, which were all assessed at baseline. We used calendar dates as the time scale. The proportional hazard assumptions were confirmed graphically using Schoenfeld’s residuals.

Missing values for individual dairy products were imputed as zero intakes [25]. The small proportion of missing data reported on single items, regarded as zero consumption, is unlikely to represent a bias for the observed findings [25]. Of those who did not report milk consumption by the FFQ part of the questionnaire, 92% reported that they did not consume milk when posed a specific question, and 99.8% consumed less than one glass/day according to complementary open-ended questions regarding dairy consumption. Missing data [11, 15] on covariates were imputed (20 imputations) using Stata’s “mi” package (multiple imputations using chained equations). Missing information on single covariates was imputed unless the whole follow-up questionnaire was missing or the follow-up questionnaire had implausible energy intake, in which case no update of dietary information was made. Less than 1% of the participants’ data for most variables in the main cohorts were missing. The variables with the most missing data were physical activity (9%), living alone (6%), and BMI (4%).

We used age-adjusted and multivariable models for each analysis, with covariates determined by a directed acyclic graph. The models included age (splines with three knots), educational level (≤ 9, 10–12, > 12 years, other), living alone (yes, no), leisure time exercise during the past year (< 1 h/week, 1 h/week, 2–3 h/week, 4–5 h/week, > 5 h/week), walking/cycling (almost never, < 20 min/day, 20–40 min/day, 40–60 min/day, 1–1.5 h/day, > 1.5 h/day), BMI (kg/m^2^; splines with three knots), height (cm; splines with three knots), energy intake (kcal/day; splines with three knots), fermented milk (mL/day; splines with three knots in analyses of non-fermented milk) or non-fermented milk intake (mL/day in analyses of fermented milk), cheese intake (g/day), alcohol consumption (g/day), fruit and vegetables intake (servings/day), red meat intake (g/day), soft drink and juice intake (mL/day), coffee intake (mL/day), total fat intake (g/day), saturated fat intake (g/day), smoking habits (current, former, never), use of vitamin- and mineral supplements (yes, no), baseline Charlson’s weighted comorbidity index (continuous), baseline major cardiovascular disease other than IHD (yes, no; ICD9 codes 415–438 and ICD10 codes I26-I69), and baseline diabetes mellitus as a separate marker variable (yes, no). All nutrient intakes were adjusted for total energy intake using the residual method [26]. We deliberately did not include hypertension or hypercholesterolemia in the multivariable model since we consider them as intermediate variables between milk consumption and IHD.

We examined non-linear trends in risk for IHD or MI with exposure using restricted cubic spline analysis with three knots (10th, 50th, and 90th percentiles) and 100 mL per day (0.5 serving or 0.5 glass) as a reference. From these curves, we computed specific HRs at 0, 200 (1 glass), 400 (2 glasses), 600 (3 glasses), and 800 mL or 4 glasses)/day of non-fermented or fermented milk intake compared with the reference level at 100 mL per day. Non-fermented and fermented milk intakes were analyzed as total intake and non-fermented intake also according to fat content (< 0.5%, 1.5%, and 3%) of the milk consumed, as previously described [27]. In the latter analysis, for each specific fat content type of milk, we restricted the dataset to those who consumed less than one serving per day for other milk fat content types [27]. Intakes were also divided into pre-specified interval categories, with < 200, 200–399, 400–599, and ≥ 600 mL per day for non-fermented milk and 0, 0–199, 200–399, and ≥ 400 mL per day for fermented milk; the category with the lowest intake was used as a reference. We also assessed non-fermented and fermented milk intakes as continuous exposures, with HR presented per 200 mL/day.

### Stratified analyses

We performed stratified analyses within categories of covariates in which non-fermented and fermented milk intake per 200 mL/day were examined. The possibility of effect modification was evaluated by including an interaction term between the milk product and the potential effect modifier in the Cox model. We used Wald tests to compare models with and without interaction terms.

### Substitution within the group of dairy foods

We assessed HRs with 95% CIs to describe the association of substituting non-fermented for fermented milk on the risk of IHD. The substitution models included the sum of serving intakes of non-fermented, fermented milk, and cheese and specific dairy subgroup intakes (fermented milk and cheese), except the non-fermented milk intake category to be substituted. The estimated HR can be interpreted as the HR for replacing a daily serving of non-fermented milk with a serving of fermented milk.

### Sensitivity analyses

In sensitivity analyses, participants with pre-existing cardiovascular disease or diabetes before baseline were excluded because these conditions may have influenced the participants’ dietary habits and lifestyle as part of the treatment of these conditions. We tested the impact of excluding fruit yogurt from the fermented milk exposure. Using the repeat questionnaire responses, we evaluated whether a history of MI or new comorbidities was associated with subsequent non-fermented and fermented milk consumption. A complete case analysis was also performed, as well as analyses in which we retained participants with IHD before baseline or with the exclusion of participants with missing data on any dairy product item. We also tested models without time-updating of our exposures and used later questionnaire data as baselines. We repeated the analyses after excluding the first two years of follow-up to determine whether the overall results might be influenced by reverse causality.

### Analyses of cardiometabolic proteomics in sub-cohorts

In exploratory analyses using a discovery and replication design, we used data from the two sub-cohorts to examine the associations of non-fermented or fermented milk intake with plasma concentrations of 276 cardiometabolic-related proteins (Additional file: Table S1). Firstly, we selected proteins related to the intake of non-fermented milk, fermented milk, or both types of milk in linear regression analyses with the protein as an outcome. In the discovery analysis, we adjusted for multiple testing using the false discovery rate (FDR), and we conservatively applied a *Q*-value threshold of 0.05 in both minimally adjusted (age, year of blood draw) and multivariable-adjusted models. The latter included age and year of blood draw, plus the same covariates as in the multivariable-adjusted survival models described above. For reasons of stringency, in the second subcohort analysis, we deemed as replicated proteins that remained nominally statistically significant (*p* < 0.05) after age and multivariable adjustment.

Secondly, the associations between milk intake and plasma protein concentration (SD difference) were displayed in spline graphs using three knots at the 10th, 50th, and 90th percentiles of milk intake. We used zero consumption as the reference in the graphs to ease interpretation. Since the discovery cohort consists only of women, we present results for the replication cohort both overall and for women separately.

Thirdly, for the proteins deemed replicated, we used the STRING (Search Tool for the Retrieval of Interacting Genes/Proteins) database (http://string-db.org) to analyze known and predicted protein–protein interactions between replicated measured proteins and unmeasured proteins. Using three scenarios, we let STRING add related but unmeasured proteins to the network, increasing complexity consecutively. STRING derives interactions from genomic context predictions and co-expression analyses, results from high-throughput laboratory experiments, automated text mining of scientific articles, and previous knowledge accessed in databases.

In addition, using multivariable-adjusted restricted cubic splines (same model as in the main analysis), we separately examined whether non-fermented and fermented milk intakes were associated with established risk biomarkers for IHD, including plasma levels of C-reactive protein (CRP) and blood lipids (total cholesterol, low-density lipoprotein [LDL] cholesterol, high-density lipoprotein [HDL] cholesterol, and triglycerides).

Finally, for replicated proteins, we examined the multivariable-adjusted HRs for incident IHD using Cox proportional hazards regression models (the same model as in the main analysis). Results were presented as spline curves and as one-unit higher NPX values. All statistical analyses were conducted in Stata version 15.1 (StataCorp, College Station, TX, USA).

## Results

Table 1 shows the characteristics of the study population according to baseline non-fermented milk intake categories. The mean intake of was 240 mL a day in women and 290 mL a day in men. About 9% of the women (*n* = 5456) and 15% of the men (*n* = 6178) reported an intake of 600 mL/day or more. Compared with low consumers, women and men with a high non-fermented milk consumption had, on average, higher energy intake, higher consumption of red meat, modestly higher BMI, lower educational level, and lower alcohol intake. On average, reported milk intake did not change after an MI or new comorbidity (Additional file 1: Table S2). However, there were clear time trends of lowered non-fermented milk and increases in fermented milk intake.

**Table 1:** Baseline characteristics in women from the Swedish Mammography Cohort in 1987–1990 and men from the Cohort of Swedish Men in 1997, according to groups of non-fermented milk intake. Values are means (standard deviations) unless stated otherwise

	**Non-fermented milk intake categories (mL/day)**				
**Characteristics**	**0–199**	**200–399**	**400–599**	** ≥ 600**	**Total**
**Women 1987–1990**					
No of participants	16,554	22,917	15,071	5456	59,998
Age (years)	53.5 (9.5)	54.4 (9.7)	54.4 (9.8)	53.1 (9.5)	54.0 (9.7)
Body mass index (kg/m^2^)	24.4 (3.9)	24.7 (3.8)	25.0 (4.0)	24.9 (4.2)	24.7 (3.9)
Height (cm)	164.1 (5.9)	164.1 (5.8)	164.1 (5.9)	164.3 (6.0)	164.1 (5.8)
Non-fermented milk (mL/day)	17.1 (37.1)	201.6 (14.9)	400.2 (5.9)	676.4 (151.1)	243.8 (201.4)
Fermented milk (mL/day)	102.3 (116.9)	97.6 (101.1)	93.1 (104.8)	86.7 (111.3)	96.8 (107.6)
Cheese (g/day)	27.0 (21.3)	26.4 (19.6)	26.9 (19.9)	27.8 (22.0)	26.8 (20.4)
Alcohol intake (g/day)	3.1 (4.0)	2.6 (3.5)	2.1 (3.0)	2.0 (3.0)	2.6 (3.5)
Coffee intake (cups/day)	2.3 (1.1)	2.4 (1.0)	2.4 (1.0)	2.5 (1.1)	2.4 (1.1)
Fruit and vegetable intake (serv/day)	3.5 (2.0)	3.3 (1.8)	3.2 (1.9)	3.1 (2.0)	3.3 (1.9)
Red meat intake (g/day)	70.6 (42.0)	75.8 (39.9)	80.0 (40.5)	85.6 (44.7)	76.3 (41.4)
Soft drink intake (serv/day)	0.5 (0.7)	0.5 (0.6)	0.5 (0.6)	0.5 (0.7)	0.5 (0.6)
Energy intake (kcal/day)	1413.9 (433.0)	1538.1 (414.1)	1708.7 (433.4)	1965.7 (524.2)	1585.6 (464.1)
Fat intake (g/day)	54.4 (9.7)	54.7 (8.6)	54.5 (8.8)	54.6 (9.7)	54.5 (9.1)
Saturated fat intake (g/day)	23.5 (5.6)	24.1 (5.2)	24.3 (5.6)	25.0 (6.3)	24.1 (5.5)
Diabetes mellitus	185 (1.1%)	322 (1.4%)	195 (1.3%)	81 (1.5%)	783 (1.3%)
Weighted Charlson comorbidity index					
0	15,250 (92.1%)	21,260 (92.8%)	13,881 (92.1%)	4954 (90.8%)	55,345 (92.2%)
1	977 (5.9%)	1215 (5.3%)	888 (5.9%)	368 (6.7%)	3448 (5.7%)
2 or more	327 (2.0%)	442 (1.9%)	302 (2.0%)	134 (2.5%)	1205 (2.0%)
Prevalent cardiovascular disease	221 (1.3%)	331 (1.4%)	251 (1.7%)	91 (1.7%)	894 (1.5%)
Smoking status^a^					
Current	5766 (23.1%)	1571 (23.2%)	553 (27.5%)	153 (25.1%)	8043 (23.4%)
Former	6082 (24.3%)	1294 (19.1%)	370 (18.4%)	145 (23.8%)	7891 (22.9%)
Never	13,148 (52.6%)	3917 (57.8%)	1085 (54.0%)	311 (51.1%)	18,461 (53.7%)
Walking/bicycling^a^					
Never/seldom	2386 (10.2%)	714 (11.2%)	249 (13.2%)	61 (10.9%)	3410 (10.6%)
< 20 min/day	4472 (19.0%)	1215 (19.0%)	317 (16.9%)	88 (15.7%)	6092 (18.9%)
20–40 min/day	8244 (35.1%)	2135 (33.5%)	610 (32.4%)	180 (32.1%)	11,169 (34.6%)
40–60 min/day	4368 (18.6%)	1210 (19.0%)	353 (18.8%)	105 (18.8%)	6036 (18.7%)
1–1.5 h/day	2414 (10.3%)	646 (10.1%)	197 (10.5%)	67 (12.0%)	3324 (10.3%)
> 1.5 h/day	1594 (6.8%)	459 (7.2%)	154 (8.2%)	59 (10.5%)	2266 (7.0%)
Exercise^a^					
< 1 h/week	4448 (19.4%)	1208 (19.6%)	348 (19.4%)	115 (21.7%)	6119 (19.5%)
1 h/week	5506 (24.1%)	1390 (22.6%)	408 (22.7%)	107 (20.2%)	7411 (23.6%)
2–3 h/week	7745 (33.9%)	2136 (34.7%)	599 (33.4%)	159 (30.0%)	10,639 (33.9%)
4–5 h/week	2651 (11.6%)	749 (12.2%)	216 (12.0%)	66 (12.5%)	3682 (11.7%)
> 5 h/week	2523 (11.0%)	678 (11.0%)	225 (12.5%)	83 (15.7%)	3509 (11.2%)
Living alone	3838 (23.4%)	5134 (22.6%)	3606 (24.2%)	1391 (25.7%)	13,969 (23.5%)
Education (years)					
≤ 9	11,031 (67.3%)	15,747 (69.3%)	10,965 (73.4%)	3858 (71.4%)	41,601 (69.9%)
10–12	1639 (10.0%)	2046 (9.0%)	1219 (8.2%)	444 (8.2%)	5348 (9.0%)
> 12	2371 (14.5%)	3098 (13.6%)	1553 (10.4%)	594 (11.0%)	7616 (12.8%)
Other	1361 (8.3%)	1838 (8.1%)	1206 (8.1%)	509 (9.4%)	4914 (8.3%)
**Men 1997**					
No of participants	18,303	9609	6687	6178	40,777
Age (years)	59.3 (9.4)	60.8 (9.7)	60.8 (9.7)	59.9 (9.4)	60.0 (9.6)
Body mass index (kg/m^2^)	25.6 (3.2)	25.6 (3.3)	25.8 (3.3)	26.4 (3.6)	25.7 (3.3)
Height (cm)	177.5 (6.7)	177.5 (6.6)	177.3 (6.7)	177.5 (6.8)	177.5 (6.7)
Non-fermented milk (g/day)	64.2 (68.2)	267.2 (59.2)	468.0 (48.9)	915.1 (378.1)	307.2 (334.7)
Fermented milk (g/day)	182.2 (237.7)	165.5 (223.4)	177.3 (247.3)	183.3 (285.6)	177.6 (244.0)
Cheese (g/day)	74.3 (60.5)	72.3 (55.5)	76.4 (58.2)	87.8 (66.5)	76.2 (60.2)
Alcohol intake (g/day)	12.3 (11.0)	9.9 (9.5)	8.8 (9.3)	8.0 (10.1)	10.5 (10.4)
Coffee intake (cups/day)	3.4 (2.1)	3.4 (2.0)	3.6 (2.1)	4.0 (2.3)	3.5 (2.1)
Fruit and vegetable intake (serv/day)	4.0 (2.4)	4.0 (2.3)	3.8 (2.3)	3.5 (2.3)	3.9 (2.4)
Red meat intake (g/day)	103.4 (63.4)	103.9 (62.2)	104.9 (57.7)	110.6 (59.6)	104.8 (61.7)
Soft drink intake (serv/day)	0.9 (1.3)	0.9 (1.1)	0.9 (1.2)	1.1 (1.5)	0.9 (1.3)
Energy intake (kcal/day)	2534.4 (792.7)	2612.7 (772.6)	2772.5 (791.8)	3163.1 (913.4)	2687.1 (836.1)
Fat intake (g/day)	89.9 (15.4)	89.8 (14.4)	89.6 (14.8)	89.5 (15.4)	89.8 (15.1)
Saturated fat intake (g/day)	40.5 (9.5)	40.9 (9.0)	41.4 (9.4)	42.4 (10.0)	41.0 (9.5)
Diabetes mellitus	1323 (7.2%)	854 (8.9%)	599 (9.0%)	539 (8.7%)	3315 (8.1%)
Weighted Charlson comorbidity index					
0	16,367 (89.4%)	8490 (88.4%)	5916 (88.5%)	5428 (87.9%)	36,201 (88.8%)
1	1443 (7.9%)	845 (8.8%)	573 (8.6%)	547 (8.9%)	3408 (8.4%)
2 or more	493 (2.7%)	274 (2.9%)	198 (3.0%)	203 (3.3%)	1168 (2.9%)
Prevalent cardiovascular disease	1201 (6.6%)	727 (7.6%)	461 (6.9%)	457 (7.4%)	2846 (7.0%)
Smoking status					
Current	4292 (23.7%)	2231 (23.5%)	1732 (26.4%)	1786 (29.4%)	10,041 (25.0%)
Former	7273 (40.2%)	3451 (36.4%)	2339 (35.6%)	2161 (35.5%)	15,224 (37.9%)
Never	6508 (36.0%)	3800 (40.1%)	2499 (38.0%)	2137 (35.1%)	14,944 (37.2%)
Walking/bicycling					
Never/seldom	2221 (13.4%)	1092 (12.5%)	803 (13.2%)	785 (14.2%)	4901 (13.3%)
< 20 min/day	4167 (25.2%)	2096 (24.0%)	1446 (23.7%)	1353 (24.5%)	9062 (24.6%)
20–40 min/day	4972 (30.1%)	2619 (30.0%)	1761 (28.9%)	1475 (26.7%)	10,827 (29.3%)
40–60 min/day	2515 (15.2%)	1359 (15.5%)	954 (15.7%)	787 (14.2%)	5615 (15.2%)
1–1.5 h/day	1360 (8.2%)	808 (9.2%)	568 (9.3%)	503 (9.1%)	3239 (8.8%)
> 1.5 h/day	1293 (7.8%)	768 (8.8%)	560 (9.2%)	627 (11.3%)	3248 (8.8%)
Exercise					
< 1 h/week	3665 (22.4%)	1753 (20.3%)	1308 (21.9%)	1356 (25.1%)	8082 (22.2%)
1 h/week	3169 (19.3%)	1669 (19.3%)	1178 (19.7%)	981 (18.1%)	6997 (19.2%)
2–3 h/week	5182 (31.6%)	2788 (32.3%)	1836 (30.7%)	1588 (29.3%)	11,394 (31.3%)
4–5 h/week	2071 (12.6%)	1089 (12.6%)	779 (13.0%)	647 (12.0%)	4586 (12.6%)
> 5 h/week	2295 (14.0%)	1334 (15.5%)	882 (14.7%)	840 (15.5%)	5351 (14.7%)
Living alone	3002 (16.5%)	1601 (16.7%)	1173 (17.6%)	1223 (19.9%)	6999 (17.3%)
Education (years)					
≤ 9	11,627 (63.7%)	6466 (67.5%)	4850 (72.8%)	4705 (76.4%)	27,648 (68.0%)
10–12	2961 (16.2%)	1407 (14.7%)	814 (12.2%)	666 (10.8%)	5848 (14.4%)
> 12	3588 (19.7%)	1669 (17.4%)	977 (14.7%)	758 (12.3%)	6992 (17.2%)
Other	70 (0.4%)	41 (0.4%)	22 (0.3%)	27 (0.4%)	160 (0.4%)

^a^Estimated from the second examination in 1997

### Non-fermented milk and IHD

During a median of 30 years of follow-up for women and 22 years for men, we identified 9534 women and 8362 men diagnosed with incident IHD, providing a total of 17,896 cases. The number of women and men diagnosed with an incident acute MI was 5574 and 5140, respectively.

**Fig. 1:**
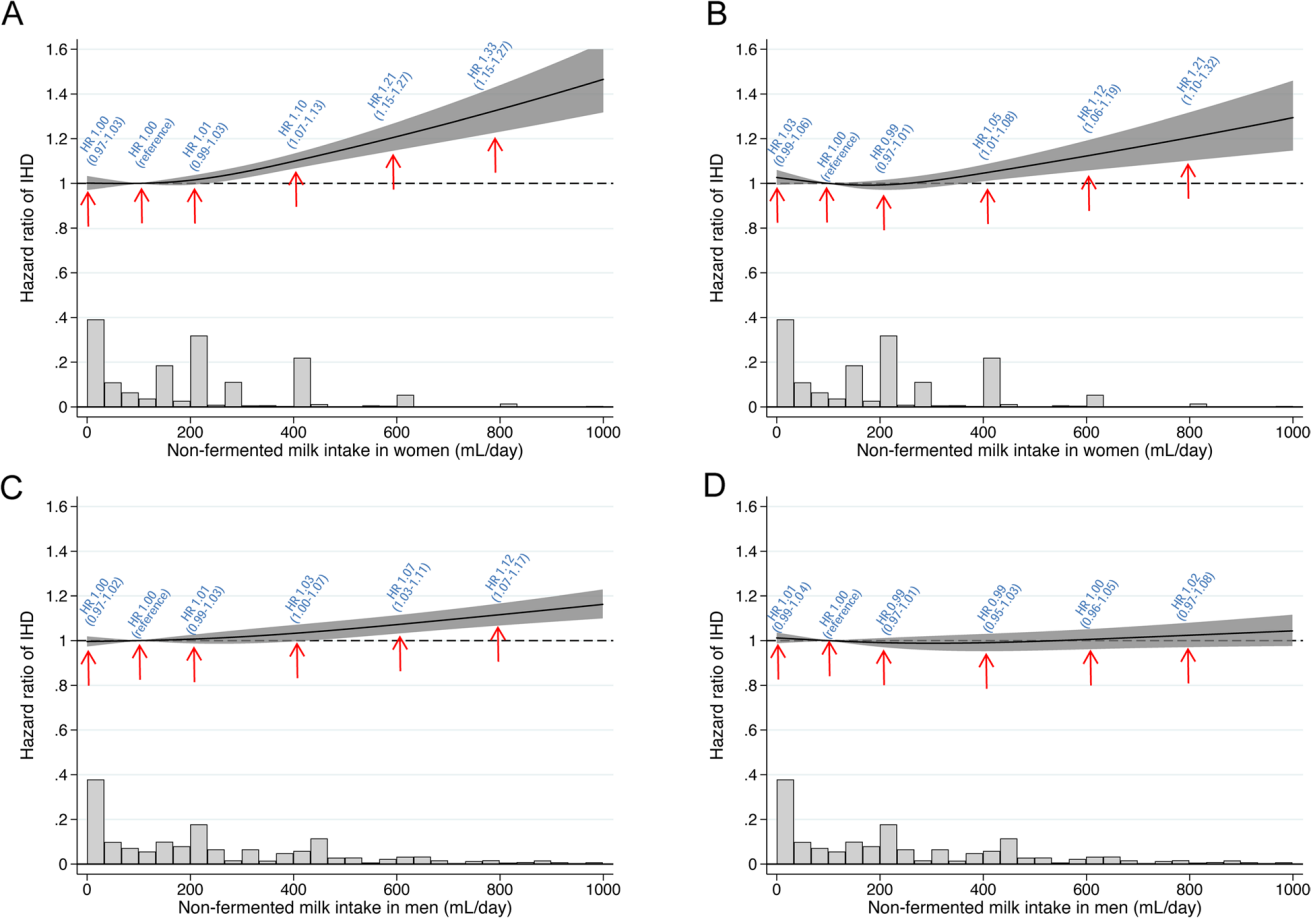
Sex-specific spline curves of the relation between non-fermented milk intake with time to ischemic heart disease. **A** (age-adjusted) and **B** (multivariable-adjusted) illustrate the pattern for women, and **C** (age-adjusted) and **D** (multivariable-adjusted) for men. Covariates were age, time-updated total energy intake, fermented milk intake, cheese intake, intake of fruit and vegetables, intake of red meat, intake of soft drinks and juice, intake of coffee, alcohol intake, total fat intake, saturated fat intake, vitamin- and mineral supplement use, body mass index, height, educational level, living alone, calcium supplementation, vitamin D supplementation, ever use of cortisone, leisure time exercise, walking/cycling, smoking status, baseline cardiovascular disease other than IHD, baseline diabetes mellitus, and baseline weighted Charlson’s comorbidity index. The bar plot shows the distribution of non-fermented milk intake. One glass of milk corresponds to 200 mL

In a non-categorical age-adjusted model with time-updated exposures, non-fermented milk intake was positively associated with the risk of IHD in both women (Fig. 1A) and men (Fig. 1C) but with larger hazard ratios in women. However, after adjustment for covariates, non-fermented milk intake was positively associated with a higher risk of IHD only in women (*p*-value = 0.01 for sex interaction performed with baseline in both women and men in 1997; Fig. 1B, D). A categorical analysis revealed similar findings (Additional file 1: Table S3). In the multivariable model, we detected a non-linear relation between non-fermented milk intake and the risk of IHD in women (Fig. 1B; *p* for non-linearity < 0.001). With an inflection point at 300 mL/day, higher intakes were associated with higher risk. Compared with an intake of 100 mL/day, a milk intake of 400 mL (2 glasses/day) conferred an HR of 1.05 (95% CI 1.01–1.08), an intake of 600 mL (three glasses per day), an HR of 1.12 (95% CI 1.06–1.19), and an intake of 800 mL (4 glasses per day) an HR of 1.21 (95% CI 1.10–1.32). We found similar patterns for the association of non-fermented milk intake with the risk of acute MI (Additional file 1: Fig. S1). In men, increasing non-fermented milk intake was not associated with higher IHD risk (Fig. 1D; *p* = 0.17).

**Fig. 2:**
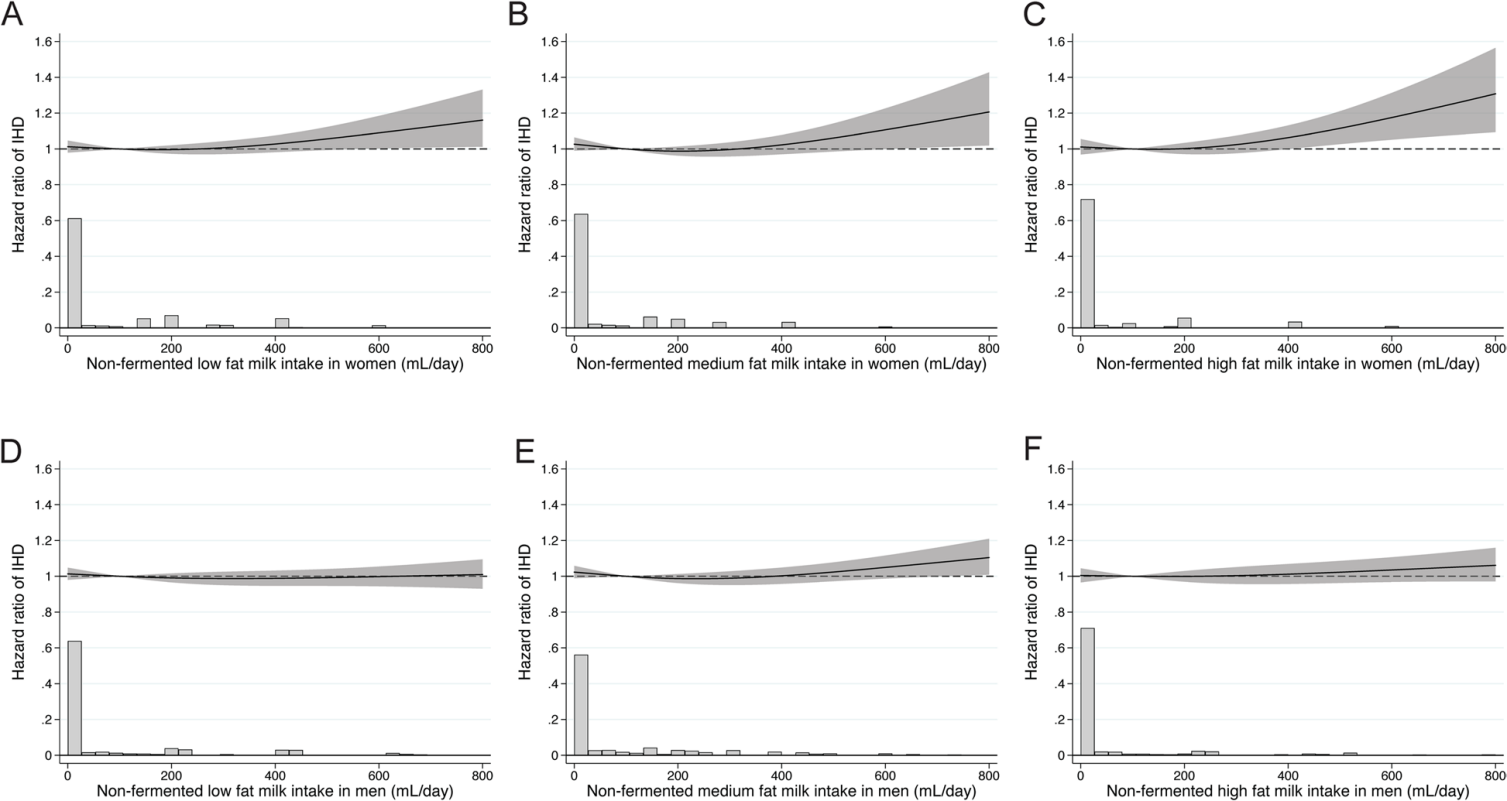
Sex-specific multivariable adjusted spline curves of the relation between non-fermented milk intake by fat content with time to ischemic heart disease. **A**–**C** illustrates the pattern for women, and **D**–**F** demonstrates the pattern for men. For each investigated fat % type of milk intake, individuals with milk intakes consisting of other fat concentrations of 200 mL or higher were censored [27]. Covariates were age, time-updated total energy intake, fermented milk intake, cheese intake, intake of fruit and vegetables, intake of red meat, intake of soft drinks and juice, intake of coffee, alcohol intake, total fat intake, saturated fat intake, vitamin- and mineral supplement use, body mass index, height, educational level, living alone, calcium supplementation, vitamin D supplementation, ever use of cortisone, leisure time exercise, walking/cycling, smoking status, baseline cardiovascular disease other than IHD, baseline diabetes mellitus, and baseline weighted Charlson’s comorbidity index. The bar plot shows the distribution of milk intake. One glass of milk corresponds to 200 mL

Associations with non-fermented milk intake were also analyzed by fat content [27]. The higher multivariable-adjusted risk of IHD was evident among women with a high milk intake irrespective of fat content: 0.5%, 1.5%, or 3% (Fig. 2A low-fat milk, Fig. 2B medium-fat milk, Fig. 2C high-fat milk). Again, the threshold for the higher risk seemed to be positioned at the 300 mL/day intake level. We detected no such pattern in men (Fig. 2D–F).

### Sensitivity analysis for the non-fermented milk and IHD associations

We obtained similar results in complete case analyses excluding those with missing data for any non-fermented milk, fermented milk, or cheese product (data not shown), with the exclusion of prevalent cardiovascular disease or diabetes (Additional file 1: Fig S2), or with the inclusion of individuals with pre-existing ischemic heart disease (data not shown). Forgoing exposure updating or using later questionnaire responses as baselines modestly attenuated the estimates (data now shown), whereas censoring of IHD events during the first 2 years of observation did not change our estimates more than marginally (data not shown).

### Fermented milk and IHD

**Fig. 3:**
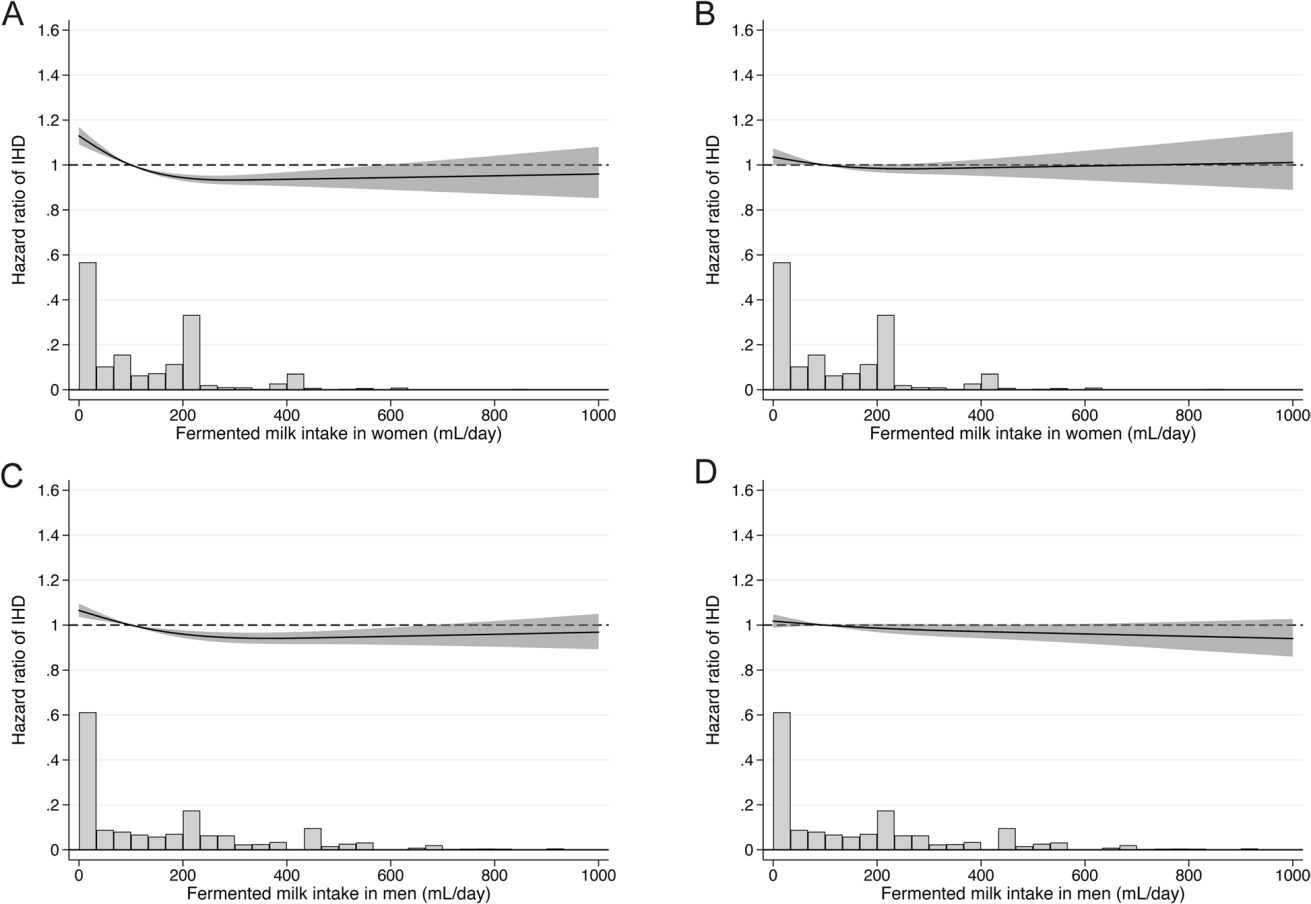
Sex-specific spline curves of the relation between fermented milk intake with time to ischemic heart disease. **A** (age-adjusted) and **B** (multivariable-adjusted) illustrate the pattern for women, and **C** (age-adjusted) and **D** (multivariable-adjusted) for men. Covariates were age, time-updated total energy intake, non-fermented milk intake, cheese intake, intake of fruit and vegetables, intake of red meat, intake of soft drinks and juice, intake of coffee, alcohol intake, total fat intake, saturated fat intake, vitamin- and mineral supplement use, body mass index, height, educational level, living alone, calcium supplementation, vitamin D supplementation, ever use of cortisone, leisure time exercise, walking/cycling, smoking status, baseline cardiovascular disease other than IHD, baseline diabetes mellitus, and baseline weighted Charlson’s comorbidity index. The bar plot shows the distribution of fermented milk intake

Although age-adjusted models suggested IHD risk reduction with fermented milk intake (Fig. 3A and C), there was no association after multivariable adjustment (Fig. 3B and D; Additional file 1: Table S4) or MI (Additional file 1: Fig S3) in either women or men (*p* = 0.51 for interaction). The multivariable-adjusted HR for IHD per 200 mL/day intake was lower for fermented milk than for non-fermented milk in both women (*p* = 0.003) and men (*p* = 0.02). The results remained similar after we excluded fermented milk products flavored with fruit and higher sugar content (data not shown).

### Substitution of non-fermented milk with fermented milk

Substitution analysis of replacing 200 mL/day non-fermented milk intake with a corresponding amount of fermented milk showed HRs for IHD of 0.95 (95% CI 0.92–0.98) in women and 0.97 (95% CI 0.95, 1.00) in men. The HRs for MI were 0.94 (95% CI 0.90–0.98) and 0.98 (95% CI 0.96–1.01) respectively.

### Stratified analyses

**Fig. 4:**
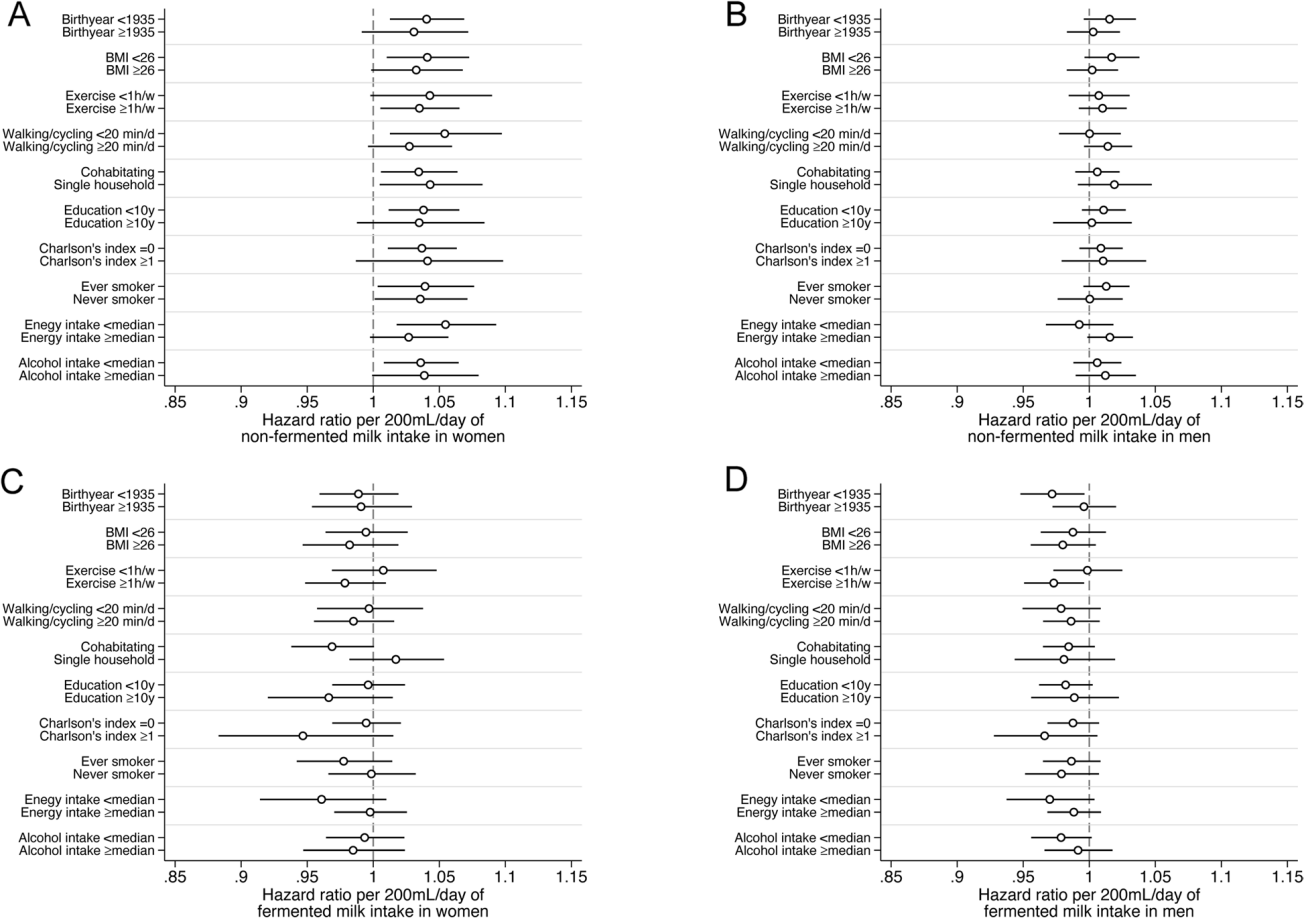
Baseline characteristic-stratified multivariable-adjusted hazard ratios of ischemic heart disease in women (**A**) and men (**B**) per time-updated serving (200 mL) of non-fermented milk or per serving (200 mL) of fermented milk intake (**C** for women and **D** for men). The model included age, time-updated total energy intake, non-fermented milk intake, cheese intake, intake of fruit and vegetables, intake of red meat, intake of soft drinks and juice, intake of coffee, alcohol intake, total fat intake, saturated fat intake, vitamin- and mineral supplement use, body mass index, height, educational level, living alone, calcium supplementation, vitamin D supplementation, ever use of cortisone, leisure time exercise, walking/cycling, smoking status, baseline cardiovascular disease other than IHD, baseline diabetes mellitus, and baseline weighted Charlson’s comorbidity index

The pattern of higher multivariable-adjusted IHD risk with increasing consumption of non-fermented milk in women was observed in all strata of BMI, socioeconomic status, physical activity, exercise level, comorbidity index, smoking status, energy intake, and alcohol intake (Fig. 4A) with *p*-values for interaction between 0.23 and 0.90). Figure 4B displays the results for men. Figures 4C (women) and 4D (men) provide similar stratified analyses with fermented milk intake as exposure.

### Association of cardiometabolic-related proteins with milk intake and with IHD

**Fig. 5:**
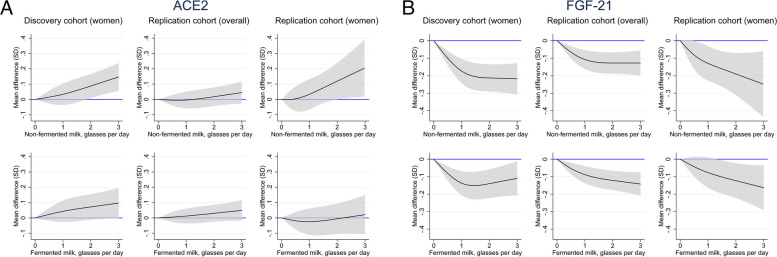
Multivariable-adjusted spline curves of the relation between non-fermented milk intake with the proteins ACE2 (**A**) and FGF-21 (**B**) in the discovery and the replication subcohort. Covariates were age, time-updated total energy intake, fermented milk intake, cheese intake, intake of fruit and vegetables, intake of red meat, intake of soft drinks and juice, intake of coffee, alcohol intake, total fat intake, saturated fat intake, vitamin- and mineral supplement use, body mass index, height, educational level, living alone, calcium supplementation, vitamin D supplementation, ever use of cortisone, leisure time exercise, walking/cycling, smoking status, baseline cardiovascular disease other than IHD, baseline diabetes mellitus, and baseline weighted Charlson’s comorbidity index

Out of 276 cardiometabolic related proteins, two had robustly replicated associations with non-fermented milk intake after FDR control in both sexes combined and in women only: angiotensin-converting enzyme 2 (ACE2) and fibroblast growth factor 21 (FGF21). ACE2 levels increased with increasing intake of non-fermented milk in women, but there was no uniform pattern for fermented milk intake (Fig. 5A). Lower concentrations of FGF21 were observed with higher non-fermented milk intake as well as with fermented milk intake but with a less clear pattern (Fig. 5B). The association was more evident in women than in men. Baseline characteristics of participants in the replication and discovery proteomics cohorts are displayed in Additional file 1: Table. S5. Moreover, the results of the association between milk intake and all analyzed proteins are shown in Additional file 1: Table S6.

Additional file 1: Fig. S4A shows a STRING network analysis displaying pathways involving ACE2 and FGF21 and their close relation with cardiometabolic proteins not measured in the current study. ACE2 is closely related to AGT (angiotensinogen), DPP4 (type 4 dipeptidyl peptidase), KLB, and TMPRSS2 (Transmembrane Serine Protease 2), and FGF21 is also related to these proteins and KLB. Adding more protein nodes to the network (Additional file 1: Fig. S4B-C) displays ACE2’s relation to REN/renin, and FGF21 is connected to several FGFs, including FGF23 and their receptors. Notably, non-fermented and fermented milk intake was unrelated to higher CRP or blood lipid concentrations (Additional file 1: Fig. S5A-J).

Additional file 1: Fig. S6 displays age-adjusted and multivariable-adjusted spline curves for the associations of ACE2 and FGF21 with IHD. Higher concentrations of both proteins were associated with higher rates of IHD in women but not men. One SD higher NPX value for ACE2 in women conferred a multivariable-adjusted HR of 1.29 (95% CI 1.07–1.55) for IHD and 1.07 (95% CI 0.81–1.41) in men (*p*-value = 0.07 for interaction). The corresponding HRs for FGF21 were 1.18 (95% CI 1.05–1.31) and 1.00 (95% CI 0.86–1.16), with a *p*-value = 0.02 for interaction. These results remained similar after mutual adjustment for the two proteins.

## Discussion

### Principal findings

Our analysis of two large cohort studies involving 100,775 participants and 17,896 clinically confirmed IHD events supports a dose–response positive association between non-fermented milk intake higher than 300 mL/day with higher rates of IHD (and acute MI specifically) in women, but not in men. The higher risk of IHD with high milk intake in women was evident, irrespective of the fat content of the milk. In contrast, fermented milk intake was unrelated to the risk of IHD in both women and men. The preliminary substitution analysis, limited by an observational design, suggested replacing non-fermented milk with moderate fermented milk intake could lower women’s IHD and MI rates. Our complementary analyses of potential protein pathways underlying the observed association showed that non-fermented milk intake was associated in different directions with circulating levels of ACE2 and FGF21 in women—two essential cardiometabolic proteins, also related to IHD in women in our study.

### Comparison with other studies

Previous cohort studies of the association between non-fermented, fermented, and total milk intake with IHD incidence are heterogeneous, showing lower risks, null findings, and higher risks with increasing milk consumption [5–8]. Discrepant results regarding IHD risk from milk intake may be due to different types of milk products investigated and different amounts of milk consumed. Study-related factors may also underly differences in findings, including sample size, follow-up time, variability in the range of intakes [28], pooled results for women and men, self-reported versus complete register-based identification of cases, and standardized 200 mL/day intake measures that can obscure non-linear associations [6–8, 29–31]. In meta-analyses, the underlying cohort studies have varied considerably in average intake levels, from < 20 mL/day to > 200 mL/day, with ranges that do not overlap at the high intake ranges [27, 28].

Only in the recent decade has there been an emphasis on analyzing different milk products separately. In the most recent meta-analysis [6], based on five studies, there was a non-significant tendency for higher risk of coronary heart disease with increasing non-fermented milk consumption (risk ratio of 1.02 (95% CI 1.00–1.04; *p* = 0.12) per 200 g higher intake of milk/day). The authors concluded that there is low confidence in the effect estimates of individual studies and meta-analyses [6]. The largest of the previous cohort studies, the EPIC study with 7198 IHD cases (2590 female cases) from 9 European countries [13], had a mean non-fermented milk intake of 150 g/day in women; the median of the highest analyzed category was approximately 400 g/day. The relative risk of IHD in women was 1.05 per 200 g/day (95% CI 0.98–1.12; *p* = 0.19), with a *p*-value of 0.47 for heterogeneity by sex, an estimate comparable to the present study HR of 1.04 (1.01, 1.06; *p* = 0.002) per 200 mL/day in women. We included more than three times the number of female cases in our analysis, and our highest analyzed category was 800 mL/day of non-fermented milk intake.

Alternative designs have also been used to assess the association of milk intake with IHD. On average, milk consumption is higher in individuals with the lactase persistence genotype, a variant used as a genetic instrument to reduce confounding of dairy exposure. Mendelian randomization studies have generally shown null results for the association between lactase persistence and IHD [32, 33]. Still, such studies cannot capture high intakes or differentiate the type of dairy consumed, and the lactase persistence genotype may also be pleiotropic [33–36]. No randomized trial has examined the effect of milk intake on the incidence of IHD, and such a trial is unlikely to be ever implemented.

We have previously reported that all-cause and cardiovascular mortality rates were increased at more moderate non-fermented milk consumption in women compared with men; the excess mortality was observed already at 2 or more glasses per day of non-fermented milk vs. < 1 glass in women, while twice the amount was necessary for observing modest excess mortality in men. We also noted that oxidative stress (urine 8-iso-PGF2α) and inflammation markers (serum interleukin 6) increased with non-fermented milk consumption but not with fermented milk [11]. We now corroborate these earlier findings by showing that higher non-fermented milk intake is associated with higher rates of IHD and MI in women and robustly to higher circulating concentrations of ACE2 and lower FGF21.

### Pathogenic mechanisms

One possible underlying biological mechanism whereby high amounts of non-fermented milk intake could impact circulating cardiometabolic protein concentrations and lead to ischemic heart disease in women involves milk’s content of lactose (glucose + galactose) in combination with an established sex-difference in galactose degradation through the Leloir pathway. The sex difference in sensitivity of galactose exposure has been confirmed experimentally [37–39], and the galactose elimination capacity is also higher in men than women [40–42]. Incomplete galactose degradation of the galactose component of lactose leads to oxidative stress and inflammation [11]. Chronic galactose exposure in animals, an established aging model, with a dose corresponding to 1–2 glasses of milk in humans [11, 43], has the potential for harm through its effects on oxidative stress and chronic inflammation [43–46].

Furthermore, galactose specifically induces cardiac aging in rodents [47]. Experimental evidence shows that galactose reduces KLB (β-Klotho) expression [48], whereas KLB substitution can eliminate D-galactose-induced aging and cardiac impairment by reducing oxidative stress [48–50]. Overexpression of the Klotho gene in mice extends life span, and the gene’s disruption induces phenotypes resembling premature human aging [51]. Treatment of human lung epithelial cells with galactose leads to increased mRNA levels of ACE2 [52]. Additionally, milk components such as branched-chained amino acids and milk-derived exosomes can stimulate the mammalian target of the rapamycin (mTORC1) pathway, leading to earlier development of age-related disorders [12]. Increased mTORC1 signaling shortens lifespan and accelerates aging-related processes such as cellular senescence and stem cell exhaustion [12]. In contrast, fermented milk is not a promoter of mTORC1-driven aging [12]. Milk-fermenting bacteria degrade milk’s branched-chained amino acids, galactose, and bioactive exosomal microRNAs that synergistically activate mTORC1 [12].

### Two potential mediating proteins between milk intake and IHD

We found that higher intakes of non-fermented milk in women conferred higher plasma concentrations of ACE2 and lower concentrations of FGF21. Both proteins are well-known cardiometabolic proteins with systemic effects in opposite directions. Higher ACE2 concentrations are a strong risk factor for cardiovascular mortality [53], while higher FGF21 levels have been shown to prevent cardiometabolic outcomes and extend animal lifespan [54, 55].

ACE2 is a membrane-bound protein with the highest levels of transcripts in the lungs, the gastrointestinal and renal systems, and all cardiac tissues. Both membrane-bound and soluble ACE2 are parts of the renin–angiotensin–aldosterone system (RAAS), and ACE2 is also involved in the breakdown of bradykinin, a potent vasodilator in the kallikrein–kinin system. Higher plasma ACE2 is a marker of dysregulation of the RAAS, and circulating ACE2 levels are a stronger risk for cardiovascular and non-cardiovascular death than classical cardiovascular risk factors (smoking, diabetes, blood pressure, BMI, and non-HDL cholesterol levels). ACE2 levels are also strong predictors of MI risk [53]. We show that higher consumption of non-fermented milk is related to higher concentrations of ACE2, and we confirmed the higher risk of IHD with increasing circulating concentrations of ACE2 [53]. There have been suggestions that targeting individuals with increased circulating ACE2 levels with intensive lifestyle or pharmacological interventions might reduce the risk of cardiometabolic health outcomes [53, 56–58]. In animal models, the concentration of angiotensin II in plasma, converted from angiotensin I by ACE2 activity, is significantly increased by galactose administration [59], whereas angiotensin II-receptor blockers can prevent galactose-induced aging effects [60]. GWAS analyses have shown that genetic variants leading to higher plasma ACE2 share risk genes with cardiovascular diseases, including IHD [58]. However, MR analysis restricted to the cis-pQTL of the ACE2 locus on chromosome X does not indicate a causal relationship between ACE2 and CVD-related outcomes [58]. Thus, there seems not to be a direct effect of circulating ACE2 on developing IHD.

Circulating FGF21, a liver-secreted protein, is an extracellularly acting regulator of lipids and energy metabolism; in extra-hepatic tissues, the protein is believed to primarily have autocrine or paracrine functions. KLB interacts with FGF receptors to enhance the binding affinity for FGF21, and loss of KLB at the receptor site renders receptors unresponsive to FGF21. KLB activation of the FGF21 protein has a protective effect on heart muscle cells [61]. FGF21 regulates several metabolic pathways, and in animal models, FGF21 has been shown to have beneficial effects on cardiometabolic outcomes and lifespan [54, 55]. Genetically determined lower circulating concentrations of FGF21 in humans seem to lead to higher plasma cholesterol and apolipoprotein B, higher CRP, higher liver enzyme concentrations, and impaired insulin sensitivity [54, 55, 62].

FGF21 is a crucial regulator of systemic metabolic effects through binding to receptors FGFR1, FGFR3, and FGFR4 (Additional file 1: Fig. S4C) [63, 64]. Recent clinical treatment trials with FGF21 mimetics have shown benefits on the atherogenic lipid profile, insulin sensitivity, hepatic fat fraction, liver damage, and fibrosis markers [65–67]. However, we found moderately higher IHD rates with increasing plasma FGF21 concentrations, a surprising finding given the effect of FGF21 mimetics. The same observation has been seen in previous cohort studies. An underlying explanation might be the fact that FGF21 levels are elevated in individuals with subclinical atherosclerosis, diabetes mellitus, and nonalcoholic fatty liver disease [68]. FGF21 elevation could be explained by a compensatory response to underlying metabolic stress or by FGF21 resistance due to impaired FGF21 signaling [68]. As with insulin resistance, this may suggest a requirement for a supraphysiological dose of FGF21 to meet concentration demands [68].

According to the STRING analysis, FGF21 and ACE2 are intimately connected with AGT, DPP4, KLB, and TMPRSS proteins. AGT is a precursor of angiotensin, a potent direct vasoconstrictor that increases blood pressure and has indirect prothrombotic effects [69, 70]. A genetic polymorphism that increases the level of circulating AGT in the blood is related to a higher risk of myocardial infarction [69].

ACE2 interacts with the cell surface protease TMPRSS2, which, together with ACE2, is upregulated with D-galactose treatment [52, 58]. TMPRSS2 is abundantly found in the endothelium of coronary arteries and pericytes of intramyocardial microvessels, and TMPRSS2 inhibitors are being studied as a potential treatment for cardiovascular disease by reducing atherothrombosis and inflammation [71].

DDP4 affects the incretin system and is related to FGF21, ACE2, and AGT. Inhibitors of DDP4 have been developed and approved for the oral treatment of type 2 diabetes, and these drugs induce FGF21 expression and reduce angiotensin levels [72]. Recent analysis of RCTs displays cardioprotective effects independent of the DDP4 inhibitor’s ability to lower blood glucose levels [73].

KLB, predominantly expressed in the liver and an obligatory coreceptor for FGF21 [65], is an anti-aging substance proposed to be a critical regulator of the development of CVD. Low levels of circulating Klotho have been linked to the occurrence and severity of CVD, while high levels are associated with reduced cardiovascular risk [65]. Polymorphisms of the KLB gene have been associated with the incidence of cardiovascular events [65]. The interest in developing FGF21-mimetics has also led to the recognition of KLB as a promising drug target for treating metabolic and cardiovascular diseases [65]. Experimental studies indicate that KLB improves endothelial function and has anti-inflammatory and anti-thrombotic action, and KLB deficiency results in continuous activation of Wnt signaling, which leads to increased production of reactive oxygen species [65, 74].

### Strengths and limitations

Our study has several strengths. The robustness of our findings was confirmed by using a time-varying measure of intake of milk products, a broad exposure range, and updated covariate information. Specifically, non-fermented and fermented milk intake and covariate information were measured repeatedly in both cohorts, and we time-updated intake to minimize exposure misclassification. The misclassification of our exposure is most likely non-differential, leading to a conservative bias of our estimates. The study was executed in a setting with a considerable variation in the range of intakes of different dairy products, and we could separately analyze the impact of non-fermented and fermented milk intake in both women and men. Our large sample size and number of outcomes provided sufficient power to detect only modestly strong associations. We included more cases in our study than the total number of cases in the last meta-analysis [6]. Unlike many earlier studies that used self-reports of outcomes, we have the advantage of virtually no loss to follow-up and complete identification of disease outcomes using our national registers and individual personal registration numbers provided to all Swedish citizens. A further strength is the linkage of milk intake to proteomics with a specific focus on cardiometabolic proteins, with a discovery and replication design. We are the first to use proteomics in relation to non-fermented and fermented milk intake.

Nonetheless, several limitations also need to be considered. Firstly, the cohort participants are predominantly of Scandinavian ancestry with a wide range and long history of non-fermented and fermented milk intake, including a relatively large proportion in the population with high intakes, which could limit the generalizability of the findings to other nationalities and races with different dairy cultural contexts and lower prevalence of the lactase persistence gene. The intake range of fermented milk intake was narrower than that of non-fermented milk intake, and on average, those with high consumption of fermented milk had historically this consumption pattern during a shorter period than the average person with increased consumption of non-fermented milk. Secondly, our observational study design could not directly establish a causal relationship between women’s non-fermented milk intake and IHD. Furthermore, cohort designs are more likely to generate non-differential misclassification of continuous exposures, leading to attenuation of risk estimates. A much larger study would be needed to disentangle any actual small differences in risk among low milk consumers in those consuming 0–200 mL/day. Exclusion of IHD events during the first 2 years of observation did not change our estimates more than marginally, which lessened the likelihood of a reverse causation phenomenon. The reporting of milk consumption did not seem to be influenced by comorbid conditions (Additional file 1: Table S3). We performed several additional types of sensitivity analyses supporting our findings. In the analysis, we have adjusted for several important covariates, but residual confounding cannot be excluded. For example, although the results were adjusted for several aspects of lifestyle (physical activity, different dietary components, and body composition) and socio-economic status (education and marital status), our observational study design may not have adequately captured additional nuances. We evaluated the possibility of a plasma protein mediation analysis for the association between milk intake and IHD, but given that the discovery subcohort with proteomics data had only 148 incident IHD cases and the replication cohort 126 cases, we find it not meaningful to present results for such analysis.

## Conclusions

Our results support a direct association between non-fermented milk intake, irrespective of fat content, at intakes higher than 300 mL/day and IHD in women but not in men. Fermented milk intake was unrelated to the risk of IHD. Substitution analysis indicated that women should choose a high intake of fermented milk over non-fermented milk. Consumption of non-fermented milk was associated with circulating levels of ACE2 and FGF21 in women, two crucial cardiometabolic proteins with complex regulatory effects, suggesting potential pathogenic mechanisms for our results.

## Supplementary information

Additional file 1. Figs. S1-S6. Fig S1. Sex-specific spline curves of the relation between non-fermented milk intake with time to myocardial infarction. Fig S2. Sex-specific spline curves of the relation between non-fermented milk intake with time to ischemic heart disease after exclusion of baseline cardiovascular disease and diabetes mellitus. Fig S3. Sex-specific spline curves of the relation between fermented milk intake with time to myocardial infarction. Fig S4. Construction of an interaction network between the two replicated and milk-intake-related proteins FGF21 and ACE2 using STRING tools. Fig S5. Multivariable-adjusted spline curves of the relation between both non-fermented and fermented milk intake with CRP and blood lipids. Fig S6. Sex-specific multivariable-adjusted spline curves of the relation between plasma concentrations of ACE2 and FGF21 with time to ischemic heart disease. Additional file 1: Tables S1-S6. Table S1. Proteins included in the proteomics analysis. Table S2. Change over time in reported daily non-fermented and fermented milk consumption in women and men. Table S3. Non-fermented milk consumption and time to ischemic heart disease. Table S4. Fermented milk consumption and time to ischemic heart disease. Table S5. Baseline characteristics of the participants in the discovery and replication cohort with proteomics data. Table S6. Linear association proteomics results for both non-fermented and fermented milk intake in the discovery and replication cohort. Additional file 1: Statistical Analysis Plan

## Data Availability

Data cannot be shared publicly because of the sensitive nature of the data and the GDPR legislation. Data are available from the national research infrastructure SIMPLER for researchers who meet the criteria for access to confidential data. Details of obtaining data from the national research infrastructure SIMPLER can be obtained at the website www.simpler4health.se. Our study has the SIMPLER project reference SIMP2019017.

## Abbreviations

IHD: Ischemic heart disease
MI: Myocardial infarction
SMC: Swedish Mammography Cohort
COSM: Cohort of Swedish Men
FFQ: Food frequency questionnaire
BMI: Body mass index
ICD: International Classification of Diseases
HR: Hazard ratio
CI: Confidence interval
PEA: Proximity extension assay
STRING: Search Tool for the Retrieval of Interacting Genes/Proteins
FDR: False discovery rate
STROBE: Strengthening the Reporting of Observational Studies in Epidemiology
FGF21: Fibroblast growth factor 21
FGFR: Fibroblast growth factor receptor
ACE2: Angiotensin-converting enzyme 2
KLB: β-Klotho
AGT: Angiotensinogen, DDP4 type 4 dipeptidyl peptidase
CRP: C-reactive protein
TMPRSS2: Transmembrane serine protease 2
RAAS: Renin–angiotensin–aldosterone system
mTORC1: Mammalian target of the rapamycin
LDL: Low-density lipoprotein cholesterol
HDL: High-density lipoprotein cholesterol
