# Supplementary material for: Non-fermented and fermented milk intake in relation to risk of ischemic heart disease and to circulating cardiometabolic proteins in swedish women and men: Two prospective longitudinal cohort studies with 100,775 participants
Source: BMC Med. 2024 Nov 8;22:483. doi: 10.1186/s12916-024-03651-1 (PMC11546556; doi:10.1186/s12916-024-03651-1)
Supplement: Supplementary file 1 — Additional file 1. Figs. S1-S6. Fig S1. Sex-specific spline curves of the relation between non-fermented milk intake with time to myocardial infarction. Fig S2. Sex-specific spline curves of the relation between non-fermented milk intake with time to ischemic heart disease after exclusion of baseline cardiovascular disease and diabetes mellitus. Fig S3. Sex-specific spline curves of the relation between fermented milk intake with time to myocardial infarction. Fig S4. Construction of an interaction network between the two replicated and milk-intake-related proteins FGF21 and ACE2 using STRING tools. Fig S5. Multivariable-adjusted spline curves of the relation between both non-fermented and fermented milk intake with CRP and blood lipids. Fig S6. Sex-specific multivariable-adjusted spline curves of the relation between plasma concentrations of ACE2 and FGF21 with time to ischemic heart disease. Additional file 1: Tables S1-S6. Table S1. Proteins included in the proteomics analysis. Table S2. Change over time in reported daily non-fermented and fermented milk consumption in women and men. Table S3. Non-fermented milk consumption and time to ischemic heart disease. Table S4. Fermented milk consumption and time to ischemic heart disease. Table S5. Baseline characteristics of the participants in the discovery and replication cohort with proteomics data. Table S6. Linear association proteomics results for both non-fermented and fermented milk intake in the discovery and replication cohort. Additional file 1: Statistical Analysis Plan [file 12916_2024_3651_MOESM1_ESM.pdf]

Additional file

Non-fermented and fermented liquid milk intake in relation to risk of ischemic heart disease and to circulating cardio-metabolic proteins in Swedish women and men: two prospective longitudinal cohort studies with 100,775 participants

Karl Michaëlsson et al

Content

Figures S1-S5

Tables S1-S6

Statistical Analysis Plan

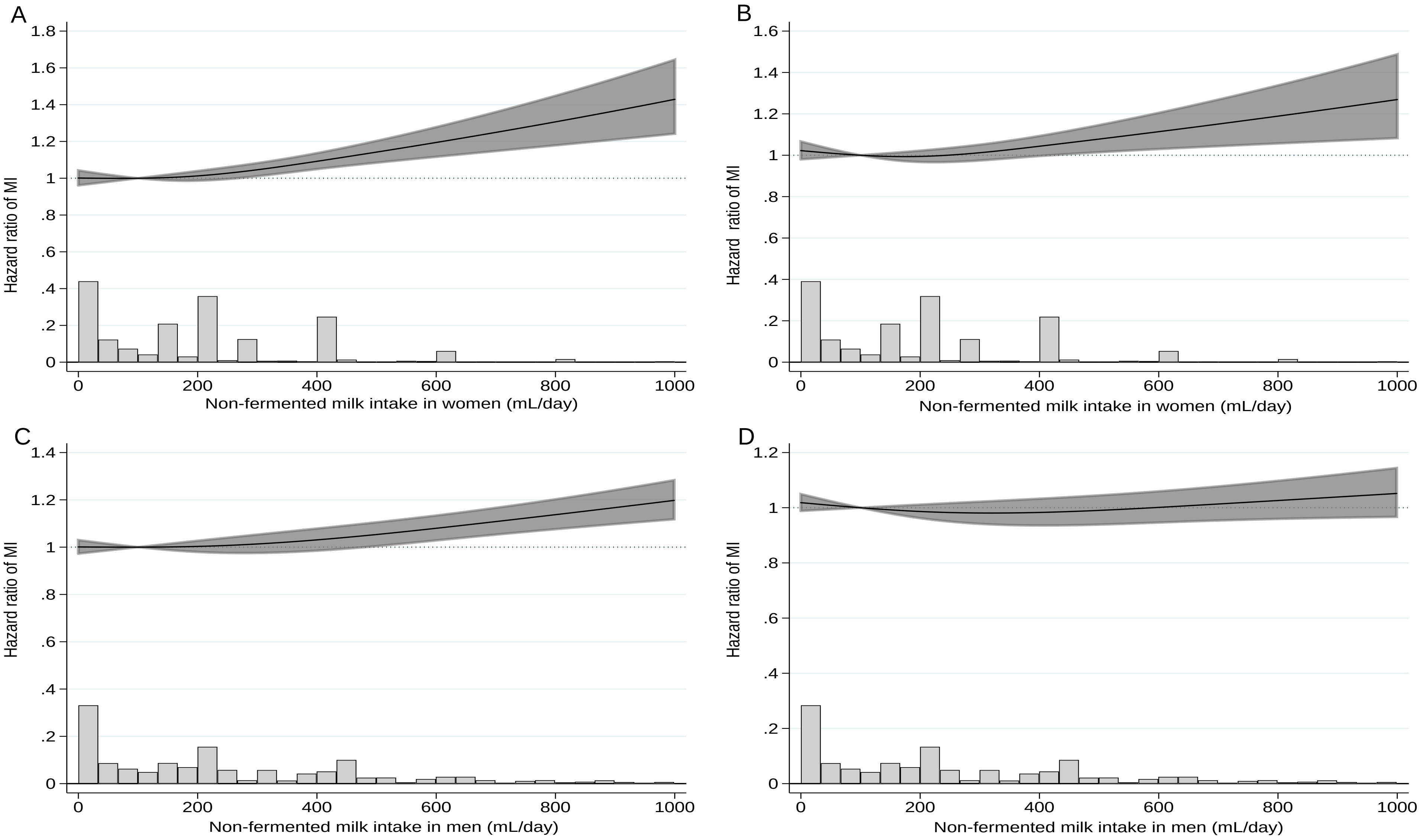

Additional File Figure S1. Sex-specific spline curves of the relation between non-fermented milk intake with time to myocardial infarction. Figure S1A (age-adjusted) and Figure S1B (multivariable-adjusted) illustrates the pattern for women, and Figure S1C (age-adjusted) and Figure S1D (multivariable-adjusted) for men. Covariates were age, time-updated total energy intake, fermented milk intake, cheese intake, intake of fruit and vegetables, intake of red meat, intake of soft drinks and juice, intakeof coffee, total fat intake, saturated fat intake, vitamin- and mineral supplement use, body mass index, height, educational level, living alone, calcium supplementation, vitamin D supplementation, ever use of cortisone, healthy dietary pattern, leisure time exercise, walking/cycling, smoking status, and baseline cardio-vascular disease, baseline diabetes mellitus, and baseline weighted Charlson's comorbidity index. The spike plot represents the distribution of non-fermented milk intake. One glass of milk corresponds to 200 mL.

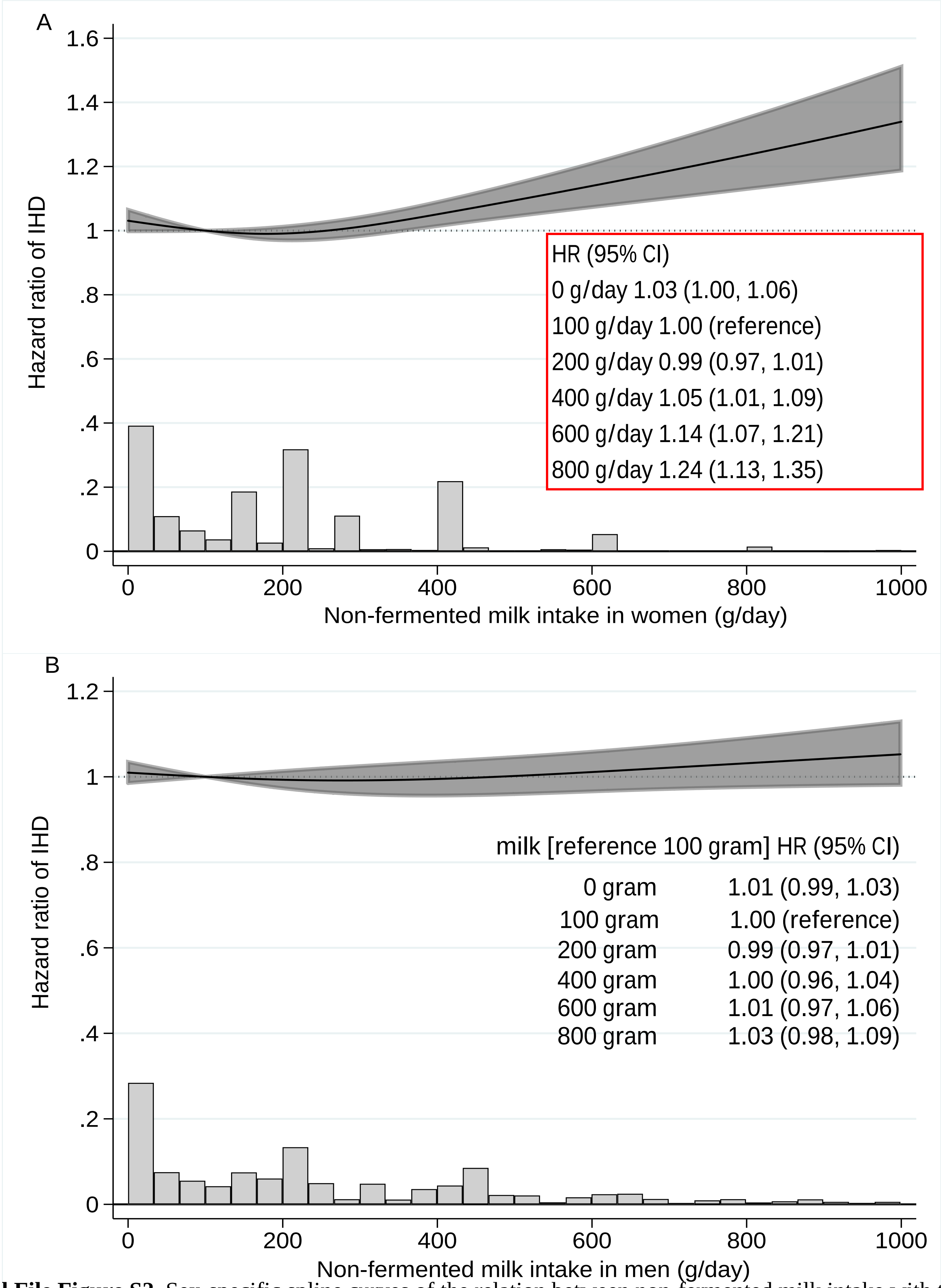

Additional File Figure S2. Sex-specific spline curves of the relation between non-fermented milk intake with time to ischemic heart disease after exclusion of baseline cardiovascular disease and diabetes mellitus. Figure S3A (multivariable-adjusted) illustrates the pattern for women, and Figure S3B (multivariable-adjusted) for men. Covariates were age, timeupdated of red meat, intake of soft drinks and juice, intake of coffee, total fat intake, saturated fat intake, vitamin- and mineral supplement use, body mass index, height, educational level, living alone, calcium supplementation, vitamin D supplementation, ever use of cortisone, healthy dietary pattern, leisure time exercise, walking/cycling, smoking status, and baseline cardiovascular disease, baseline diabetes mellitus, and baseline weighted Charlson's comorbidity index.

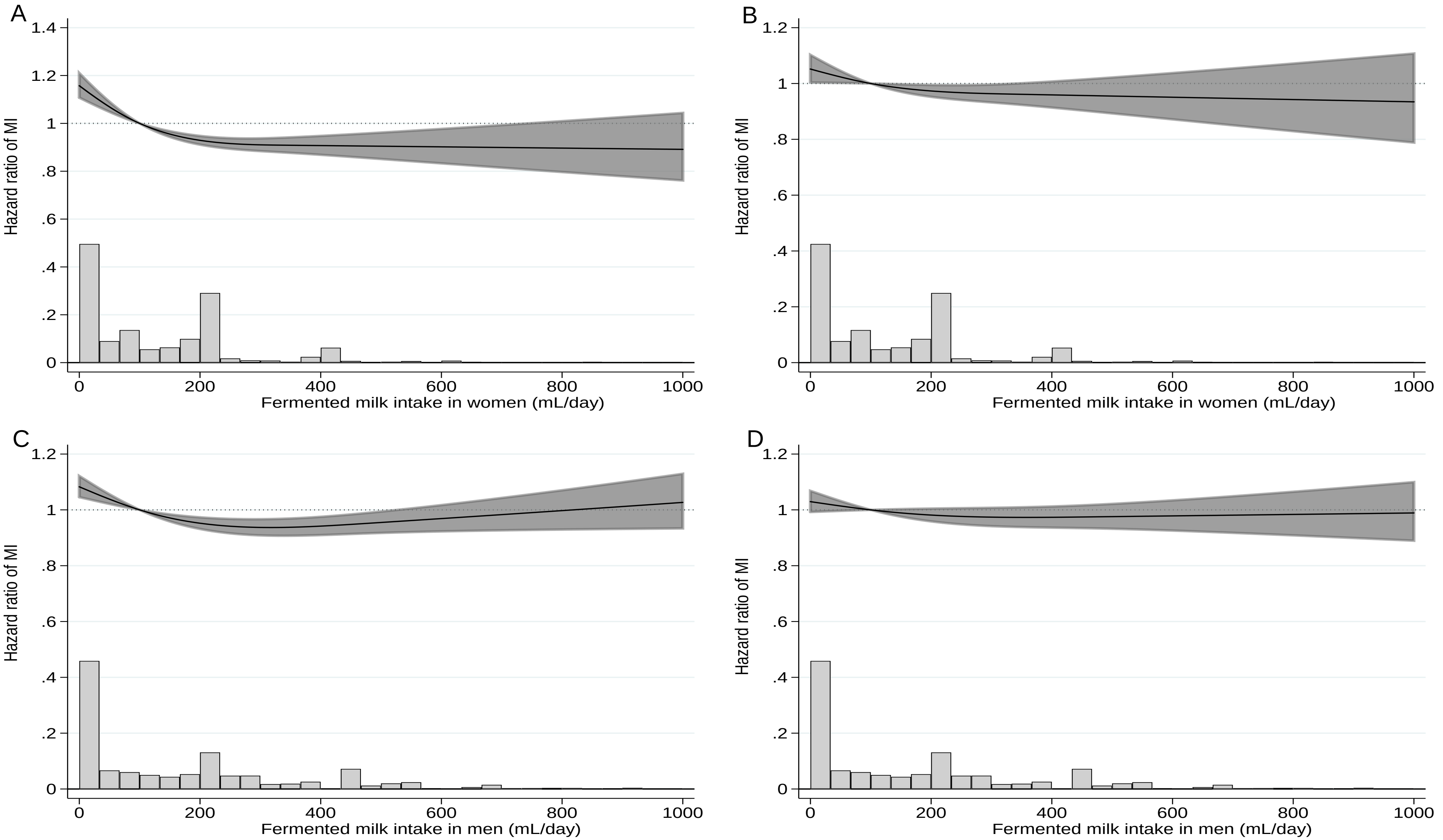

Additional File Figure S3. Sex-specific spline curves of the relation between fermented milk intake with time to myocardial infarction. Figure S2A (age-adjusted) and Figure S2B (multivariable-adjusted) illustrates the pattern for women, and Figure S2C (age-adjusted) and Figure S2D (multivariable-adjusted) for men. Covariates were age, time-updated total energy intake, non-fermented milk intake, cheese intake, intake of fruit and vegetables, intake of red meat, intake of soft drinks and juice, intake of coffee, total fat intake, saturated fat intake, vitamin- and mineral supplement use, body mass index, height, educational level, living alone, calcium supplementation, vitamin D supplementation, ever use of cortisone, healthy dietary pattern, leisure time exercise, walking/cycling, smoking status, and baseline cardiovascular disease, baseline diabetes mellitus, and baseline weighted Charlson's comorbidity index. The spike plot represents the distribution of fermented milk intake.

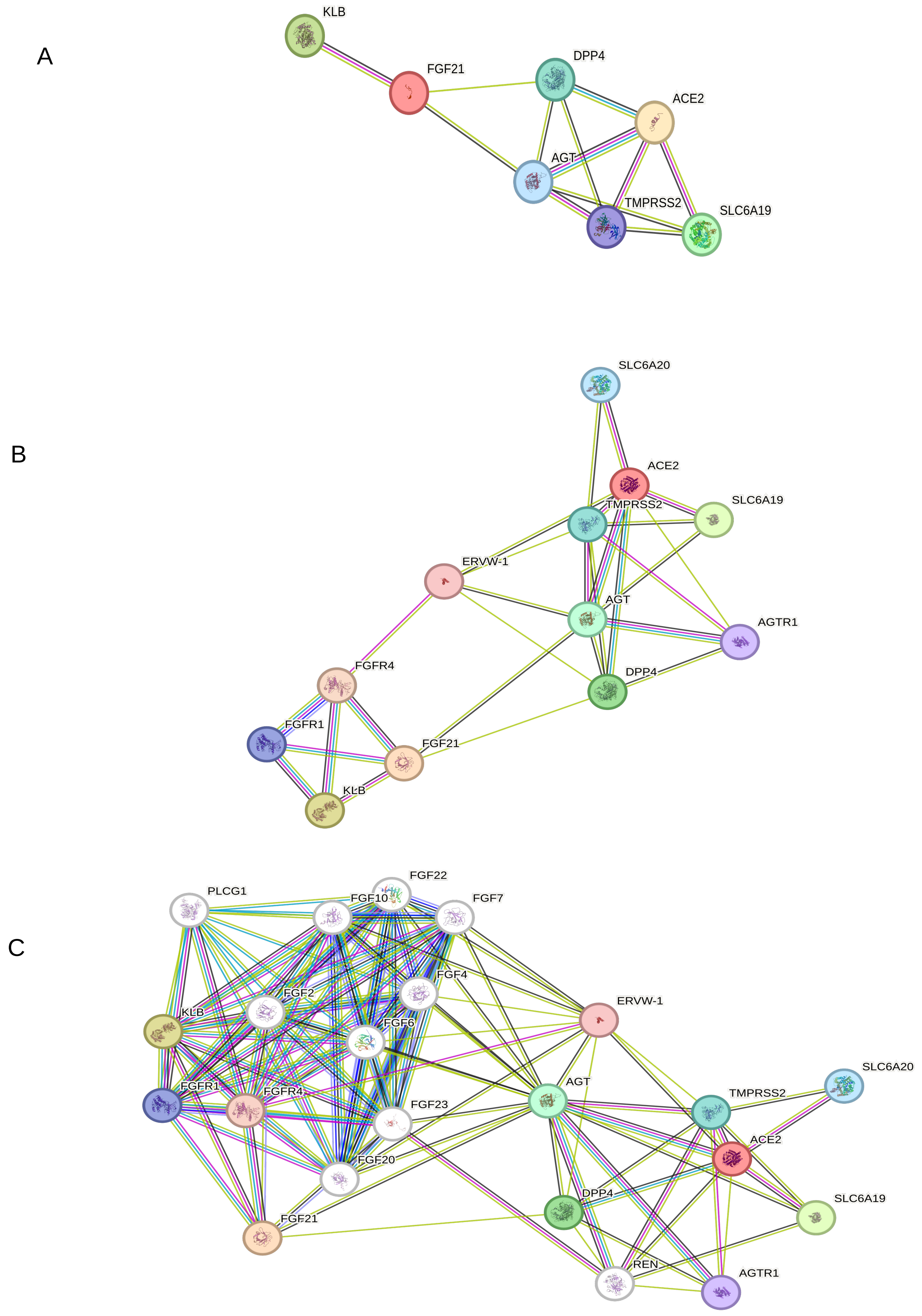

Additional File Figure S4. Construction of an interaction network between the two replicated and milk-intake-related proteins FGF21 and ACE2 using STRING tools. The STRING tool has also added proteins theoretically related to these two proteins, for each panel with an increasing number of nodes in the network. Panel A is a simple STRING-constructed network of 7 nodes, panel B has 12 nodes, and panel C has 22 nodes.

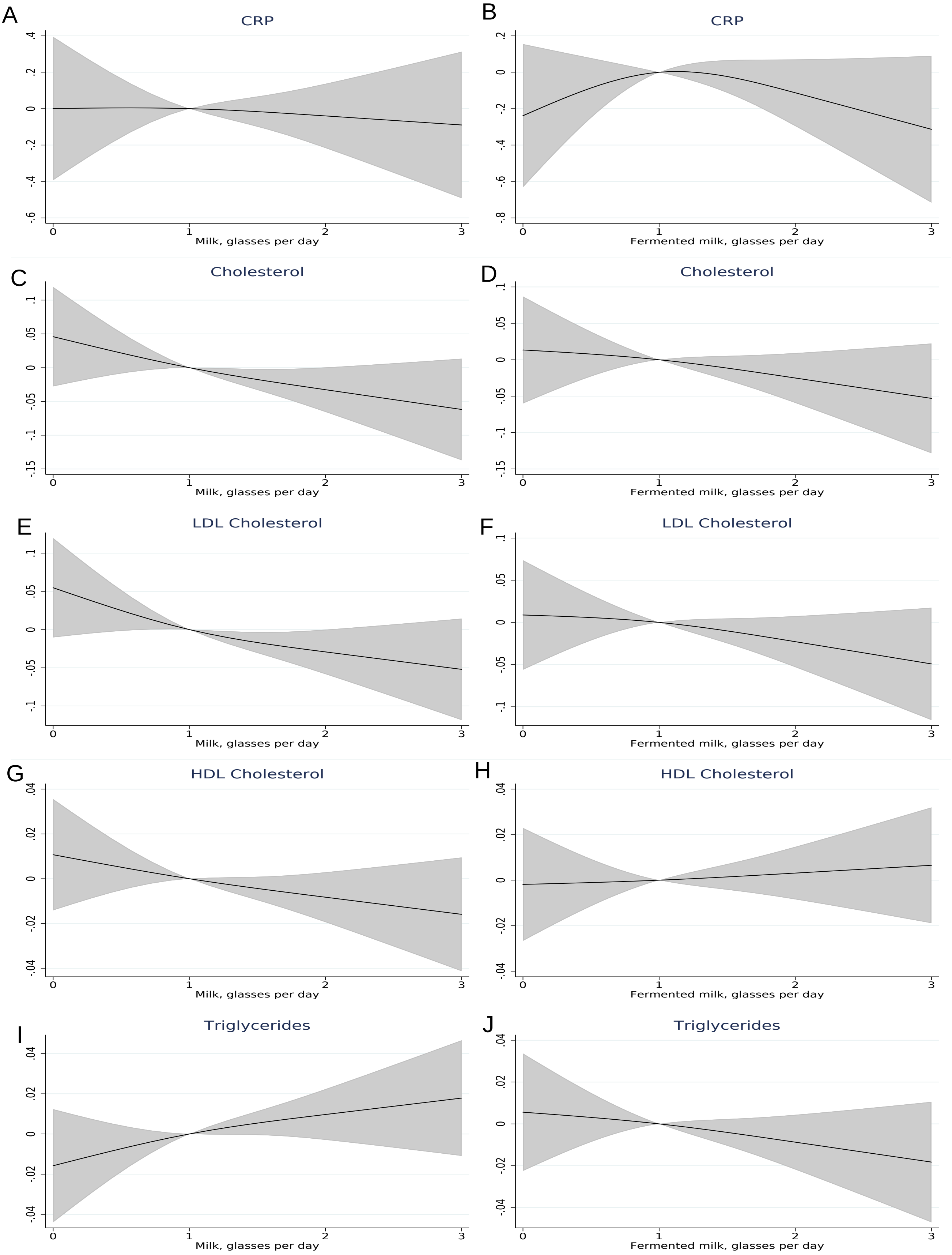

Additional File Figure S5. Multivariable-adjusted spline curves of the relation between both non-fermented and fermented milk intake with plasma CRP (Figure S5A and B), total cholesterol (Figure S5C and D), LDL cholesterol (Figure S5E and F), HDL cholesterol (Figure S5G and H, and triglycerides (Figure S5I and J) in women. Covariates were age, timeupdated total energy intake, fermented milk intake, cheese intake, intake of fruit and vegetables, intake of red meat, intake of soft drinks and juice, intake of coffee, total fat intake, saturated fat intake, vitamin- and mineral supplement use, body mass index, height, educational level, living alone, calcium supplementation, vitamin D supplementation, ever use of cortisone, healthy dietary pattern, leisure time exercise, walking/cycling, smoking status,and baseline cardiovascular disease, baseline diabetes mellitus, and baseline weighted Charlson's comorbidity index.

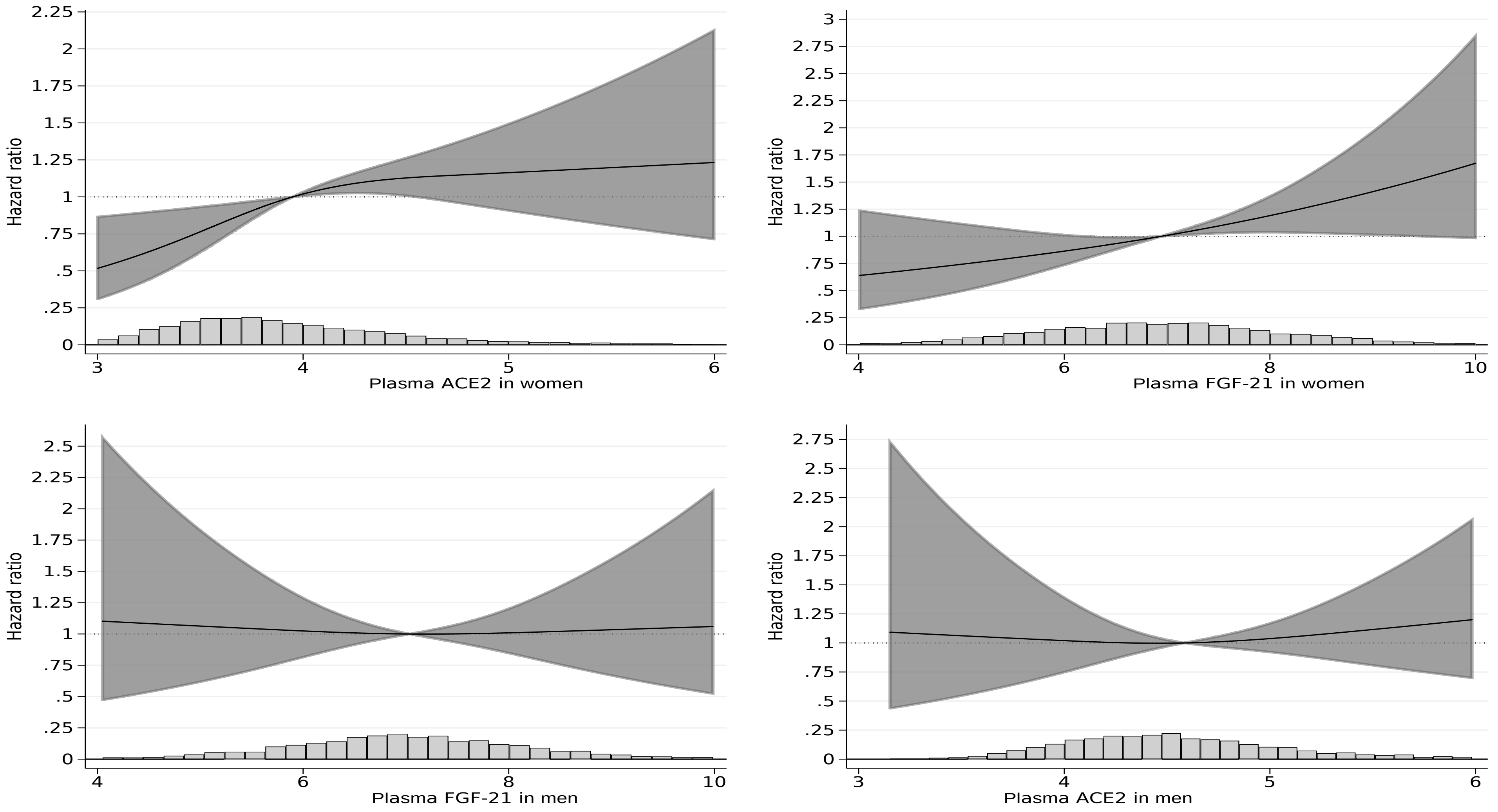

Additional File Figure S6. Sex-specific multivariable-adjusted spline curves of the relation between plasma concentrations of ACE2 and FGF21 with time to ischemic heart disease. Figure S6A and Figure S6B illustrates the associations in women and Panels C and D in men. Covariates were age, time-updated total energy intake, fermented milk intake, cheese intake, intake of fruit and vegetables, intake of red meat, intake of soft drinks and juice, intake of coffee, total fat intake, saturated fat intake, vitamin- and mineral supplement use, body mass index, height, educational level, living alone, calcium supplementation, vitamin D supplementation, ever use of cortisone, healthy dietary pattern, leisure time exercise, walking/cycling, smoking status, and baseline cardiovascular disease, baseline diabetes mellitus, and baseline weighted Charlson's comorbidity index.

Additional File Table S1. All 276 proteins included in the proteomics analysis, sorted by the short protein name.

Long protein name (short name)	Short protein name	Uniprot ID	OlinkID	Panel
Angiotensin-converting enzyme 2 (ACE2)	ACE2	Q9BYF1	OID00457	Olink CARDIOVASCULAR II(v.5003)
Lysophosphatidic acid phosphatase type 6 (ACP6)	ACP6		OID01129	Olink METABOLISM(v.3401)
A disintegrin and metalloproteinase with thrombospondin motifs 13 (ADAM-TS13)	ADAM-TS13	Q76LX8	OID00385	Olink CARDIOVASCULAR II(v.5003)
Adhesion G protein-coupled receptor E2 (ADGRE2)	ADGRE2	Q9UHX3	OID01180	Olink METABOLISM(v.3401)
Adhesion G-protein coupled receptor G2 (ADGRG2)	ADGRG2	Q8IZP9	OID01152	Olink METABOLISM(v.3401)
ADM (ADM)	ADM	P35318	OID00381	Olink CARDIOVASCULAR II(v.5003)
Agouti-related protein (AGRP)	AGRP	O00253	OID00448	Olink CARDIOVASCULAR II(v.5003)
Adenosylhomocysteinase (AHCY)	AHCY	P23526	OID01123	Olink METABOLISM(v.3401)
CD166 antigen (ALCAM)	ALCAM	Q13740	OID00572	Olink CARDIOVASCULAR III(v.6112)
Retinal dehydrogenase 1 (ALDH1A1)	ALDH1A1	P00352	OID01156	Olink METABOLISM(v.3401)
Protein AMBP (AMBP)	AMBP	P02760	OID00430	Olink CARDIOVASCULAR II(v.5003)
Angiopoietin-1 (ANG-1)	ANG-1	Q15389	OID00380	Olink CARDIOVASCULAR II(v.5003)
Angiopoietin-2 (ANGPT2)	ANGPT2	O15123	OID01132	Olink METABOLISM(v.3401)
Angiopoietin-related protein 1 (ANGPTL1)	ANGPTL1	O95841	OID01150	Olink METABOLISM(v.3401)
Angiopoietin-related protein 7 (ANGPTL7)	ANGPTL7	O43827	OID01134	Olink METABOLISM(v.3401)
Annexin A11 (ANXA11)	ANXA11	P50995	OID01159	Olink METABOLISM(v.3401)
Annexin A4 (ANXA4)	ANXA4	P09525	OID01158	Olink METABOLISM(v.3401)
DNA-(apurinic or apyrimidinic site) lyase (APEX1)	APEX1	P27695	OID01164	Olink METABOLISM(v.3401)
Amyloid-like protein 1 (APLP1)	APLP1	P51693	OID01151	Olink METABOLISM(v.3401)
Aminopeptidase N (AP-N)	AP-N	P15144	OID00611	Olink CARDIOVASCULAR III(v.6112)
Arginase-1 (ARG1)	ARG1	P05089	OID01199	Olink METABOLISM(v.3401)
Tyrosine-protein kinase receptor UFO (AXL)	AXL	P30530	OID00612	Olink CARDIOVASCULAR III(v.6112)
Azurocidin (AZU1)	AZU1	P20160	OID00597	Olink CARDIOVASCULAR III(v.6112)
Large proline-rich protein BAG6 (BAG6)	BAG6	P46379	OID01197	Olink METABOLISM(v.3401)
Bleomycin hydrolase (BLM hydrolase)	BLM hydrolase	Q13867	OID00581	Olink CARDIOVASCULAR III(v.6112)
Bone morphogenetic protein 6 (BMP-6)	BMP-6	P22004	OID00379	Olink CARDIOVASCULAR II(v.5003)
Brain natriuretic peptide (BNP)	BNP	P16860	OID00455	Olink CARDIOVASCULAR II(v.5003)
Brother of CDO (Protein BOC)	BOC	Q9BWV1	OID00386	Olink CARDIOVASCULAR II(v.5003)

Carbonic anhydrase 13 (CA13)	CA13	Q8N1Q1	OID01146	Olink METABOLISM(v.3401)
Carbonic anhydrase 5A, mitochondrial (CA5A)	CA5A	P35218	OID00464	Olink CARDIOVASCULAR II(v.5003)
Soluble calcium-activated nucleotidase 1 (CANT1)	CANT1	Q8WVQ1	OID01186	Olink METABOLISM(v.3401)
Caspase-3 (CASP-3)	CASP-3	P42574	OID00630	Olink CARDIOVASCULAR III(v.6112)
Coiled-coil domain-containing protein 80 (CCDC80)	CCDC80	Q76M96	OID01144	Olink METABOLISM(v.3401)
C-C motif chemokine 15 (CCL15)	CCL15	Q16663	OID00629	Olink CARDIOVASCULAR III(v.6112)
C-C motif chemokine 16 (CCL16)	CCL16	O15467	OID00654	Olink CARDIOVASCULAR III(v.6112)
C-C motif chemokine 17 (CCL17)	CCL17	Q92583	OID00439	Olink CARDIOVASCULAR II(v.5003)
C-C motif chemokine 24 (CCL24)	CCL24	O00175	OID00592	Olink CARDIOVASCULAR III(v.6112)
C-C motif chemokine 3 (CCL3)	CCL3	P10147	OID00440	Olink CARDIOVASCULAR II(v.5003)
Scavenger receptor cysteine-rich type 1 protein M130 (CD163)	CD163	Q86VB7	OID00577	Olink CARDIOVASCULAR III(v.6112)
Sialomucin core protein 24 (CD164)	CD164	Q04900	OID01127	Olink METABOLISM(v.3401)
T-cell surface glycoprotein CD1c (CD1C)	CD1C	P29017	OID01187	Olink METABOLISM(v.3401)
CD2-associated protein (CD2AP)	CD2AP	Q9Y5K6	OID01133	Olink METABOLISM(v.3401)
T-cell surface glycoprotein CD4 (CD4)	CD4	P01730	OID00466	Olink CARDIOVASCULAR II(v.5003)
CD40 ligand (CD40-L)	CD40-L	P29965	OID00382	Olink CARDIOVASCULAR II(v.5003)
B-cell antigen receptor complex-associated protein beta chain (CD79B)	CD79B	P40259	OID01157	Olink METABOLISM(v.3401)
SLAM family member 5 (CD84)	CD84	Q9UIB8	OID00420	Olink CARDIOVASCULAR II(v.5003)
Complement component C1q receptor (CD93)	CD93	Q9NPY3	OID00639	Olink CARDIOVASCULAR III(v.6112)
Cadherin-2 (CDH2)	CDH2	P19022	OID01189	Olink METABOLISM(v.3401)
Cadherin-5 (CDH5)	CDH5	P33151	OID00587	Olink CARDIOVASCULAR III(v.6112)
Cadherin-related family member 5 (CDHR5)	CDHR5	Q9HBB8	OID01143	Olink METABOLISM(v.3401)
Carcinoembryonic antigenrelated cell adhesion molecule 8 (CEACAM8)	CEACAM8	P31997	OID00436	Olink CARDIOVASCULAR II(v.5003)
Chitinase-3-like protein 1 (CHI3L1)	CHI3L1	P36222	OID00633	Olink CARDIOVASCULAR III(v.6112)
Chitotriosidase-1 (CHIT1)	CHIT1	Q13231	OID00605	Olink CARDIOVASCULAR III(v.6112)
Chordin-like protein 2 (CHRDL2)	CHRDL2	Q6WN34	OID01170	Olink METABOLISM(v.3401)
C-type lectin domain family 5 member A (CLEC5A)	CLEC5A	Q9NY25	OID01135	Olink METABOLISM(v.3401)
CXADR-like membrane protein (CLMP)	CLMP	Q9H6B4	OID01120	Olink METABOLISM(v.3401)
Calsyntenin-2 (CLSTN2)	CLSTN2	Q9H4D0	OID01166	Olink METABOLISM(v.3401)
Clusterin-like protein 1 (CLUL1)	CLUL1	Q15846	OID01168	Olink METABOLISM(v.3401)
Contactin-1 (CNTN1)	CNTN1	Q12860	OID00586	Olink CARDIOVASCULAR III(v.6112)

Collagen alpha-1(I) chain (COL1A1)	COL1A1	P02452	OID00641	Olink CARDIOVASCULAR III(v.6112)
Catechol O-methyltransferase (COMT)	COMT	P21964	OID01167	Olink METABOLISM(v.3401)
Carboxypeptidase A1 (CPA1)	CPA1	P15085	OID00624	Olink CARDIOVASCULAR III(v.6112)
Carboxypeptidase B (CPB1)	CPB1	P15086	OID00632	Olink CARDIOVASCULAR III(v.6112)
Crk-like protein (CRKL)	CRKL	P46109	OID01191	Olink METABOLISM(v.3401)
Cystatin-B (CSTB)	CSTB	P04080	OID00575	Olink CARDIOVASCULAR III(v.6112)
Chymotrypsin C (CTRC)	CTRC	Q99895	OID00414	Olink CARDIOVASCULAR II(v.5003)
Cathepsin D (CTSD)	CTSD	P07339	OID00622	Olink CARDIOVASCULAR III(v.6112)
Pro-cathepsin H (CTSH)	CTSH	P09668	OID01171	Olink METABOLISM(v.3401)
Cathepsin L1 (CTSL1)	CTSL1	P07711	OID00459	Olink CARDIOVASCULAR II(v.5003)
Cathepsin O (CTSO)	CTSO	P43234	OID01125	Olink METABOLISM(v.3401)
Cathepsin Z (CTSZ)	CTSZ	Q9UBR2	OID00643	Olink CARDIOVASCULAR III(v.6112)
C-X-C motif chemokine 1 (CXCL1)	CXCL1	P09341	OID00404	Olink CARDIOVASCULAR II(v.5003)
C-X-C motif chemokine 16 (CXCL16)	CXCL16	Q9H2A7	OID00601	Olink CARDIOVASCULAR III(v.6112)
Disabled homolog 2 (DAB2)	DAB2	P98082	OID01205	Olink METABOLISM(v.3401)
Decorin (DCN)	DCN	P07585	OID00444	Olink CARDIOVASCULAR II(v.5003)
Aromatic-L-amino-acid decarboxylase (DDC)	DDC	P20711	OID01128	Olink METABOLISM(v.3401)
2,4-dienoyl-CoA reductase, mitochondrial (DECR1)	DECR1	Q16698	OID00424	Olink CARDIOVASCULAR II(v.5003)
Diablo homolog, mitochondrial (DIABLO)	DIABLO	Q9NR28	OID01145	Olink METABOLISM(v.3401)
Dickkopf-related protein 1 (Dkk-1)	Dkk-1	O94907	OID00445	Olink CARDIOVASCULAR II(v.5003)
Protein delta homolog 1 (DLK-1)	DLK-1	P80370	OID00598	Olink CARDIOVASCULAR III(v.6112)
Dipeptidyl peptidase 2 (DPP7)	DPP7	Q9UHL4	OID01178	Olink METABOLISM(v.3401)
Epidermal growth factor receptor (EGFR)	EGFR	P00533	OID00637	Olink CARDIOVASCULAR III(v.6112)
Gamma-enolase (ENO2)	ENO2	P09104	OID01138	Olink METABOLISM(v.3401)
Ectonucleotide pyrophosphatase/phosphodiesterase family member 7 (ENPP7)	ENPP7	Q6UWV6	OID01181	Olink METABOLISM(v.3401)
Ectonucleoside triphosphate diphosphohydrolase 5 (ENTPD5)	ENTPD5	O75356	OID01165	Olink METABOLISM(v.3401)
Epithelial cell adhesion molecule (Ep-CAM)	Ep-CAM	P16422	OID00610	Olink CARDIOVASCULAR III(v.6112)
Ephrin type-B receptor 4 (EPHB4)	EPHB4	P54760	OID00569	Olink CARDIOVASCULAR III(v.6112)
Fatty acid-binding protein, intestinal (FABP2)	FABP2	P12104	OID00451	Olink CARDIOVASCULAR II(v.5003)
Fatty acid-binding protein, adipocyte (FABP4)	FABP4	P15090	OID00589	Olink CARDIOVASCULAR III(v.6112)
Protein FAM3C (FAM3C)	FAM3C	Q92520	OID01175	Olink METABOLISM(v.3401)

Tumor necrosis factor receptor superfamily member 6 (FAS)	FAS	P25445	OID00615	Olink CARDIOVASCULAR III(v.6112)
Fructose-1,6-bisphosphatase 1 (FBP1)	FBP1	P09467	OID01195	Olink METABOLISM(v.3401)
Fc receptor-like protein 1 (FCRL1)	FCRL1	Q96LA6	OID01126	Olink METABOLISM(v.3401)
Fibroblast growth factor 21 (FGF-21)	FGF-21	Q9NSA1	OID00410	Olink CARDIOVASCULAR II(v.5003)
Fibroblast growth factor 23 (FGF-23)	FGF-23	Q9GZV9	OID00415	Olink CARDIOVASCULAR II(v.5003)
Peptidyl-prolyl cis-trans isomerase FKBP4 (FKBP4)	FKBP4	Q02790	OID01201	Olink METABOLISM(v.3401)
Follistatin (FS)	FS	P19883	OID00418	Olink CARDIOVASCULAR II(v.5003)
Galanin peptides (GAL)	GAL	P22466	OID01188	Olink METABOLISM(v.3401)
Galectin-3 (Gal-3)	Gal-3	P17931	OID00578	Olink CARDIOVASCULAR III(v.6112)
Galectin-4 (Gal-4)	Gal-4	P56470	OID00626	Olink CARDIOVASCULAR III(v.6112)
Galectin-9 (Gal-9)	Gal-9	O00182	OID00406	Olink CARDIOVASCULAR II(v.5003)
Growth/differentiation factor 15 (GDF-15)	GDF-15	Q99988	OID00595	Olink CARDIOVASCULAR III(v.6112)
Growth/differentiation factor 2 (GDF-2)	GDF-2	Q9UK05	OID00450	Olink CARDIOVASCULAR II(v.5003)
Growth hormone (GH)	GH	P01241	OID00417	Olink CARDIOVASCULAR II(v.5003)
Appetite-regulating hormone (GHRL)	GHRL	Q9UBU3	OID01140	Olink METABOLISM(v.3401)
Gastric intrinsic factor (GIF)	GIF	P27352	OID00407	Olink CARDIOVASCULAR II(v.5003)
Lactoylglutathione lyase (GLO1)	GLO1	Q04760	OID00419	Olink CARDIOVASCULAR II(v.5003)
Glutaredoxin-1 (GLRX)	GLRX	P35754	OID01137	Olink METABOLISM(v.3401)
Platelet glycoprotein VI (GP6)	GP6	Q9HCN6	OID05026	Olink CARDIOVASCULAR III(v.6112)
GRB2-related adapter protein 2 (GRAP2)	GRAP2	O75791	OID01154	Olink METABOLISM(v.3401)
Granulins (GRN)	GRN	P28799	OID00579	Olink CARDIOVASCULAR III(v.6112)
Gastrotropin (GT)	GT	P51161	OID00454	Olink CARDIOVASCULAR II(v.5003)
Hydroxyacid oxidase 1 (HAOX1)	HAOX1	Q9UJM8	OID00470	Olink CARDIOVASCULAR II(v.5003)
Proheparin-binding EGF-like growth factor (HB-EGF)	HB-EGF	Q99075	OID00449	Olink CARDIOVASCULAR II(v.5003)
Hepatoma-derived growth factor (HDGF)	HDGF	P51858	OID01169	Olink METABOLISM(v.3401)
Heme oxygenase 1 (HO-1)	HO-1	P09601	OID00432	Olink CARDIOVASCULAR II(v.5003)
Osteoclast-associated immunoglobulin-like receptor (hOSCAR)	hOSCAR	Q8IYS5	OID00460	Olink CARDIOVASCULAR II(v.5003)
Heat shock 27 kDa protein (HSP 27)	HSP 27	P04792	OID00465	Olink CARDIOVASCULAR II(v.5003)
Intercellular adhesion molecule 2 (ICAM-2)	ICAM-2	P13598	OID00646	Olink CARDIOVASCULAR III(v.6112)
Alpha-L-iduronidase (IDUA)	IDUA	P35475	OID00393	Olink CARDIOVASCULAR II(v.5003)
Insulin-like growth factor-binding protein 1 (IGFBP-1)	IGFBP-1	P08833	OID00604	Olink CARDIOVASCULAR III(v.6112)

Insulin-like growth factor-binding protein 2 (IGFBP-2)	IGFBP-2	P18065	OID00650	Olink CARDIOVASCULAR III(v.6112)
Insulin-like growth factor-binding protein 7 (IGFBP-7)	IGFBP-7	Q16270	OID00638	Olink CARDIOVASCULAR III(v.6112)
Insulin-like growth factor-binding protein-like 1 (IGFBPL1)	IGFBPL1	Q8WX77	OID01192	Olink METABOLISM(v.3401)
Low affinity immunoglobulin gamma Fc region receptor II-b (IgG Fc receptor II-b)	IgG Fc receptor II-b	P31994	OID00442	Olink CARDIOVASCULAR II(v.5003)
Pro-interleukin-16 (IL16)	IL16	Q14005	OID00434	Olink CARDIOVASCULAR II(v.5003)
Interleukin-17D (IL-17D)	IL-17D	Q8TAD2	OID00403	Olink CARDIOVASCULAR II(v.5003)
Interleukin-17 receptor A (IL-17RA)	IL-17RA	Q96F46	OID00566	Olink CARDIOVASCULAR III(v.6112)
Interleukin-18 (IL-18)	IL18	Q14116	OID00409	Olink CARDIOVASCULAR II(v.5003)
Interleukin-18-binding protein (IL-18BP)	IL-18BP	O95998	OID00640	Olink CARDIOVASCULAR III(v.6112)
Interleukin-1 receptor antagonist protein (IL-1ra)	IL-1ra	P18510	OID00389	Olink CARDIOVASCULAR II(v.5003)
Interleukin-1 receptor-like 2 (IL1RL2)	IL1RL2	Q9HB29	OID00400	Olink CARDIOVASCULAR II(v.5003)
Interleukin-1 receptor type 1 (IL-1RT1)	IL-1RT1	P14778	OID00613	Olink CARDIOVASCULAR III(v.6112)
Interleukin-1 receptor type 2 (IL-1RT2)	IL-1RT2	P27930	OID00627	Olink CARDIOVASCULAR III(v.6112)
Interleukin-27 (IL-27)	IL-27	Q8NEV9, Q14213	OID00402	Olink CARDIOVASCULAR II(v.5003)
Interleukin-2 receptor subunit alpha (IL2-RA)	IL2-RA	P01589	OID00570	Olink CARDIOVASCULAR III(v.6112)
Interleukin-4 receptor subunit alpha (IL-4RA)	IL-4RA	P24394	OID00387	Olink CARDIOVASCULAR II(v.5003)
Interleukin-6 (IL6)	IL6	P05231	OID00390	Olink CARDIOVASCULAR II(v.5003)
Interleukin-6 receptor subunit alpha (IL-6RA)	IL-6RA	P08887	OID00602	Olink CARDIOVASCULAR III(v.6112)
Melusin (ITGB1BP2)	ITGB1BP2	Q9UKP3	OID00443	Olink CARDIOVASCULAR II(v.5003)
Integrin beta-2 (ITGB2)	ITGB2	P05107	OID00565	Olink CARDIOVASCULAR III(v.6112)
Integrin beta-7 (ITGB7)	ITGB7	P26010	OID01161	Olink METABOLISM(v.3401)
Junctional adhesion molecule A (JAM-A)	JAM-A	Q9Y624	OID00625	Olink CARDIOVASCULAR III(v.6112)
Kidney Injury Molecule (KIM1)	KIM1	Q96D42	OID00426	Olink CARDIOVASCULAR II(v.5003)
Kallikrein-10 (KLK10)	KLK10	O43240	OID01148	Olink METABOLISM(v.3401)
Kallikrein-6 (KLK6)	KLK6	Q92876	OID00647	Olink CARDIOVASCULAR III(v.6112)
Kynurenine-oxoglutarate transaminase 1 (KYAT1)	KYAT1	Q16773	OID01204	Olink METABOLISM(v.3401)
Low-density lipoprotein receptor (LDL receptor)	LDL receptor	P01130	OID00564	Olink CARDIOVASCULAR III(v.6112)
Leptin (LEP)	LEP	P41159	OID00463	Olink CARDIOVASCULAR II(v.5003)
Leukocyte immunoglobulin-like receptor subfamily A member 5 (LILRA5)	LILRA5	A6NI73	OID01155	Olink METABOLISM(v.3401)
Lectin-like oxidized LDL receptor 1 (LOX-1)	LOX-1	P78380	OID00405	Olink CARDIOVASCULAR II(v.5003)

Lipoprotein lipase (LPL)	LPL	P06858	OID00446	Olink CARDIOVASCULAR II(v.5003)
Leucine-rich repeats and immunoglobulin-like domains protein 1 (LRIG1)	LRIG1	Q96JA1	OID01121	Olink METABOLISM(v.3401)
Low-density lipoprotein receptor-related protein 11 (LRP11)	LRP11	Q86VZ4	OID01179	Olink METABOLISM(v.3401)
Lymphotoxin-beta receptor (LTBR)	LTBR	P36941	OID00583	Olink CARDIOVASCULAR III(v.6112)
Macrophage receptor MARCO (MARCO)	MARCO	Q9UEW3	OID00453	Olink CARDIOVASCULAR II(v.5003)
Myoglobin (MB)	MB	P02144	OID00616	Olink CARDIOVASCULAR III(v.6112)
Multiple coagulation factor deficiency protein 2 (MCFD2)	MCFD2	Q8NI22	OID01183	Olink METABOLISM(v.3401)
Monocyte chemotactic protein 1 (MCP-1)	MCP-1	P13500	OID00576	Olink CARDIOVASCULAR III(v.6112)
Meprin A subunit beta (MEP1B)	MEP1B	Q16820	OID01208	Olink METABOLISM(v.3401)
Matrix extracellular phosphoglycoprotein (MEPE)	MEPE	Q9NQ76	OID00132	Olink CARDIOVASCULAR III(v.6112)
Tyrosine-protein kinase Mer (MERTK)	MERTK	Q12866	OID00425	Olink CARDIOVASCULAR II(v.5003)
Meteorin-like protein (METRNL)	METRNL	Q641Q3	OID01207	Olink METABOLISM(v.3401)
Matrix metalloproteinase-12 (MMP-12)	MMP12	P39900	OID00456	Olink CARDIOVASCULAR II(v.5003)
Matrix metalloproteinase-2 (MMP-2)	MMP-2	P08253	OID00614	Olink CARDIOVASCULAR III(v.6112)
Matrix metalloproteinase-3 (MMP-3)	MMP-3	P08254	OID00644	Olink CARDIOVASCULAR III(v.6112)
Matrix metalloproteinase-7 (MMP-7)	MMP7	P09237	OID00441	Olink CARDIOVASCULAR II(v.5003)
Matrix metalloproteinase-9 (MMP-9)	MMP-9	P14780	OID00568	Olink CARDIOVASCULAR III(v.6112)
Myeloperoxidase (MPO)	MPO	P05164	OID00600	Olink CARDIOVASCULAR III(v.6112)
NAD kinase (NADK)	NADK	O95544	OID01139	Olink METABOLISM(v.3401)
Nectin-2 (NECTIN2)	NECTIN2	Q92692	OID01198	Olink METABOLISM(v.3401)
NF-kappa-B essential modulator (NEMO)	NEMO	Q9Y6K9	OID00467	Olink CARDIOVASCULAR II(v.5003)
Nodal modulator 1 (NOMO1)	NOMO1	Q15155	OID01172	Olink METABOLISM(v.3401)
Neurogenic locus notch homolog protein 3 (Notch 3)	Notch 3	Q9UM47	OID00584	Olink CARDIOVASCULAR III(v.6112)
Neural proliferation differentiation and control protein 1 (NPDC1)	NPDC1	Q9NQX5	OID01206	Olink METABOLISM(v.3401)
Neuronal pentraxin receptor (NPTXR)	NPTXR	O95502	OID01122	Olink METABOLISM(v.3401)
Ribosyldihydronicotinamide dehydrogenase [quinone] (NQO2)	NQO2	P16083	OID01173	Olink METABOLISM(v.3401)
N-terminal prohormone brain natriuretic peptide (NT-proBNP)	NT-proBNP	NA	OID01214	Olink METABOLISM(v.3401)
N-terminal prohormone brain natriuretic peptide (NT-proBNP)	NT-proBNP	NA	OID00131	Olink CARDIOVASCULAR III(v.6112)
Osteoprotegerin (OPG)	OPG	O00300	OID00571	Olink CARDIOVASCULAR III(v.6112)
Osteopontin (OPN)	OPN	P10451	OID00621	Olink CARDIOVASCULAR III(v.6112)
Phosphoprotein associated with glycosphingolipid-enriched microdomains 1 (PAG1	PAG1	Q9NWQ8	OID01203	Olink METABOLISM(v.3401)

Plasminogen activator inhibitor 1 (PAI)	PAI	P05121	OID00591	Olink CARDIOVASCULAR III(v.6112)
Pappalysin-1 (PAPPA)	PAPPA	Q13219	OID00421	Olink CARDIOVASCULAR II(v.5003)
Proteinase-activated receptor 1 (PAR-1)	PAR-1	P25116	OID00395	Olink CARDIOVASCULAR II(v.5003)
Poly [ADP-ribose] polymerase 1 (PARP-1)	PARP-1	P09874	OID00469	Olink CARDIOVASCULAR II(v.5003)
Proprotein convertase subtilisin/kexin type 9 (PCSK9)	PCSK9	Q8NBP7	OID00619	Olink CARDIOVASCULAR III(v.6112)
Platelet-derived growth factor subunit A (PDGF subunit A)	PDGF subunit A	P04085	OID00648	Olink CARDIOVASCULAR III(v.6112)
Platelet-derived growth factor subunit B (PDGF subunit B)	PDGF subunit B	P01127	OID00401	Olink CARDIOVASCULAR II(v.5003)
Programmed cell death 1 ligand 2 (PD-L2)	PD-L2	Q9BQ51	OID00458	Olink CARDIOVASCULAR II(v.5003)
Platelet endothelial cell adhesion molecule (PECAM-1)	PECAM-1	P16284	OID00652	Olink CARDIOVASCULAR III(v.6112)
Placenta growth factor (PGF)	PGF	P49763	OID00384	Olink CARDIOVASCULAR II(v.5003)
Peptidoglycan recognition protein 1 (PGLYRP1)	PGLYRP1	O75594	OID00623	Olink CARDIOVASCULAR III(v.6112)
Elafin (PI3)	PI3	P19957	OID00609	Olink CARDIOVASCULAR III(v.6112)
Polymeric immunoglobulin receptor (PIgR)	PIgR	P01833	OID00411	Olink CARDIOVASCULAR II(v.5003)
Paired immunoglobulin-like type 2 receptor beta (PILRB)	PILRB	Q9UKJ0	OID01149	Olink METABOLISM(v.3401)
Perlecan (PLC)	PLC	P98160	OID00582	Olink CARDIOVASCULAR III(v.6112)
Paraoxonase (PON3)	PON3	Q15166	OID00642	Olink CARDIOVASCULAR III(v.6112)
Protein phosphatase inhibitor 2 (PPP1R2)	PPP1R2	P41236	OID01177	Olink METABOLISM(v.3401)
Prolargin (PRELP)	PRELP	P51888	OID00431	Olink CARDIOVASCULAR II(v.5003)
Serine protease 27 (PRSS27)	PRSS27	Q9BQR3	OID00397	Olink CARDIOVASCULAR II(v.5003)
Prostasin (PRSS8)	PRSS8	Q16651	OID00447	Olink CARDIOVASCULAR II(v.5003)
Myeloblastin (PRTN3)	PRTN3	P24158	OID00618	Olink CARDIOVASCULAR III(v.6112)
P-selectin glycoprotein ligand 1 (PSGL-1)	PSGL-1	Q14242	OID00438	Olink CARDIOVASCULAR II(v.5003)
Pulmonary surfactant-associated protein D (PSP-D)	PSP-D	P35247	OID00608	Olink CARDIOVASCULAR III(v.6112)
Pentraxin-related protein PTX3 (PTX3)	PTX3	P26022	OID00437	Olink CARDIOVASCULAR II(v.5003)
Dihydropteridine reductase (QDPR)	QDPR	P09417	OID01162	Olink METABOLISM(v.3401)
Receptor for advanced glycosylation end products (RAGE)	RAGE	Q15109	OID00412	Olink CARDIOVASCULAR II(v.5003)
Retinoic acid receptor responder protein 2 (RARRES2)	RARRES2	Q99969	OID00645	Olink CARDIOVASCULAR III(v.6112)
Regenerating islet-derived protein 4 (REG4)	REG4	Q9BYZ8	OID01184	Olink METABOLISM(v.3401)
Renin (REN)	REN	P00797	OID00423	Olink CARDIOVASCULAR II(v.5003)
Resistin (RETN)	RETN	Q9HD89	OID00603	Olink CARDIOVASCULAR III(v.6112)
Eosinophil cationic protein (RNASE3)	RNASE3	P12724	OID01211	Olink METABOLISM(v.3401)

Inactive tyrosine-protein kinase transmembrane receptor ROR1 (ROR1)	ROR1	Q01973	OID01209	Olink METABOLISM(v.3401)
Reticulon-4 receptor (RTN4R)	RTN4R	Q9BZR6	OID01193	Olink METABOLISM(v.3401)
Protein S100-P (S100P)	S100P	P25815	OID01131	Olink METABOLISM(v.3401)
Stem cell factor (SCF)	SCF	P21583	OID00408	Olink CARDIOVASCULAR II(v.5003)
Secretoglobin family 3A member 2 (SCGB3A2)	SCGB3A2	Q96PL1	OID00636	Olink CARDIOVASCULAR III(v.6112)
Syndecan-4 (SDC4)	SDC4	P31431	OID01202	Olink METABOLISM(v.3401)
E-selectin (SELE)	SELE	P16581	OID00596	Olink CARDIOVASCULAR III(v.6112)
P-selectin (SELP)	SELP	P16109	OID00574	Olink CARDIOVASCULAR III(v.6112)
Semaphorin-3F (SEMA3F)	SEMA3F	Q13275	OID01147	Olink METABOLISM(v.3401)
Serpin A12 (SERPINA12)	SERPINA12	Q8IW75	OID00422	Olink CARDIOVASCULAR II(v.5003)
Serpin B6 (SERPINB6)	SERPINB6	P35237	OID01142	Olink METABOLISM(v.3401)
Serpin B8 (SERPINB8)	SERPINB8	P50452	OID01141	Olink METABOLISM(v.3401)
Tyrosine-protein phosphatase non-receptor type substrate 1 (SHPS-1)	SHPS-1	P78324	OID00628	Olink CARDIOVASCULAR III(v.6112)
Sialic acid-binding Ig-like lectin 7 (SIGLEC7)	SIGLEC7	Q9Y286	OID01160	Olink METABOLISM(v.3401)
SLAM family member 7 (SLAMF7)	SLAMF7	Q9NQ25	OID00383	Olink CARDIOVASCULAR II(v.5003)
Synaptosomal-associated protein 23 (SNAP23)	SNAP23	O00161	OID01163	Olink METABOLISM(v.3401)
Superoxide dismutase [Mn], mitochondrial (SOD2)	SOD2	P04179	OID00413	Olink CARDIOVASCULAR II(v.5003)
Sortilin (SORT1)	SORT1	Q99523	OID00435	Olink CARDIOVASCULAR II(v.5003)
Sclerostin (SOST)	SOST	Q9BQB4	OID01174	Olink METABOLISM(v.3401)
Spondin-1 (SPON1)	SPON1	Q9HCB6	OID00599	Olink CARDIOVASCULAR III(v.6112)
Spondin-2 (SPON2)	SPON2	Q9BUD6	OID00416	Olink CARDIOVASCULAR II(v.5003)
Proto-oncogene tyrosine-protein kinase Src (SRC)	SRC	P12931	OID00388	Olink CARDIOVASCULAR II(v.5003)
Scavenger receptor cysteine-rich domain-containing group B protein (SSC4D)	SSC4D	Q8WTU2	OID01182	Olink METABOLISM(v.3401)
ST2 protein (ST2)	ST2	Q01638	OID00634	Olink CARDIOVASCULAR III(v.6112)
Serine/threonine-protein kinase 4 (STK4)	STK4	Q13043	OID00392	Olink CARDIOVASCULAR II(v.5003)
Sulfatase-modifying factor 2 (SUMF2)	SUMF2	Q8NBJ7	OID01185	Olink METABOLISM(v.3401)
Tissue factor (TF)	TF	P13726	OID00399	Olink CARDIOVASCULAR II(v.5003)
Trefoil factor 2 (TFF2)	TFF2	Q03403	OID01130	Olink METABOLISM(v.3401)
Trefoil factor 3 (TFF3)	TFF3	Q07654	OID00573	Olink CARDIOVASCULAR III(v.6112)
Tissue factor pathway inhibitor (TFPI)	TFPI	P10646	OID00590	Olink CARDIOVASCULAR III(v.6112)
Protein-glutamine gamma-glutamyltransferase 2 (TGM2)	TGM2	P21980	OID00462	Olink CARDIOVASCULAR II(v.5003)

Thrombospondin-2 (THBS2)	THBS2	P35442	OID00427	Olink CARDIOVASCULAR II(v.5003)
Thimet oligopeptidase (THOP1)	THOP1	P52888	OID01124	Olink METABOLISM(v.3401)
Thrombopoietin (THPO)	THPO	P40225	OID00452	Olink CARDIOVASCULAR II(v.5003)
Angiopoietin-1 receptor (TIE2)	TIE2	Q02763	OID00398	Olink CARDIOVASCULAR II(v.5003)
Metalloproteinase inhibitor 4 (TIMP4)	TIMP4	Q99727	OID00585	Olink CARDIOVASCULAR III(v.6112)
Tubulointerstitial nephritis antigen-like (TINAGL1)	TINAGL1	Q9GZM7	OID01136	Olink METABOLISM(v.3401)
Trem-like transcript 2 protein (TLT-2)	TLT-2	Q5T2D2	OID00588	Olink CARDIOVASCULAR III(v.6112)
Thrombomodulin TM	TM	P07204	OID00428	Olink CARDIOVASCULAR II(v.5003)
Tumor necrosis factor receptor 1 (TNF-R1)	TNF-R1	P19438	OID00649	Olink CARDIOVASCULAR III(v.6112)
Tumor necrosis factor receptor 2 (TNF-R2)	TNF-R2	P20333	OID00567	Olink CARDIOVASCULAR III(v.6112)
Tumor necrosis factor receptor superfamily member 10A (TNFRSF10A)	TNFRSF10A	O00220	OID00391	Olink CARDIOVASCULAR II(v.5003)
Tumor necrosis factor receptor superfamily member 10C (TNFRSF10C)	TNFRSF10C	O14798	OID00594	Olink CARDIOVASCULAR III(v.6112)
Tumor necrosis factor receptor superfamily member 11A (TNFRSF11A)	TNFRSF11A	Q9Y6Q6	OID00394	Olink CARDIOVASCULAR II(v.5003)
Tumor necrosis factor receptor superfamily member 13B (TNFRSF13B)	TNFRSF13B	O14836	OID00461	Olink CARDIOVASCULAR II(v.5003)
Tumor necrosis factor receptor superfamily member 14 (TNFRSF14)	TNFRSF14	Q92956	OID00563	Olink CARDIOVASCULAR III(v.6112)
Tumor necrosis factor ligand superfamily member 13B (TNFSF13B)	TNFSF13B	Q9Y275	OID00617	Olink CARDIOVASCULAR III(v.6112)
Tissue-type plasminogen activator (t-PA)	t-PA	P00750	OID00635	Olink CARDIOVASCULAR III(v.6112)
Transferrin receptor protein 1 (TR)	TR	P02786	OID00593	Olink CARDIOVASCULAR III(v.6112)
TNF-related apoptosis-inducing ligand receptor 2 (TRAIL-R2)	TRAIL-R2	O14763	OID00396	Olink CARDIOVASCULAR II(v.5003)
Tartrate-resistant acid phosphatase type 5 (TR-AP)	TR-AP	P13686	OID00606	Olink CARDIOVASCULAR III(v.6112)
Thyrotropin subunit beta (TSHB)	TSHB	P01222	OID01196	Olink METABOLISM(v.3401)
Thioredoxin domain-containing protein 5 (TXNDC5)	TXNDC5	Q8NBS9	OID01176	Olink METABOLISM(v.3401)
Thymidine phosphorylase (TYMP)	TYMP	P19971	OID01153	Olink METABOLISM(v.3401)
Tyrosine-protein kinase receptor TYRO3 (TYRO3)	TYRO3	Q06418	OID01190	Olink METABOLISM(v.3401)
Urokinase-type plasminogen activator (uPA)	uPA	P00749	OID00631	Olink CARDIOVASCULAR III(v.6112)
Urokinase plasminogen activator surface receptor (U-PAR)	U-PAR	Q03405	OID00620	Olink CARDIOVASCULAR III(v.6112)
Ubiquitin carboxyl-terminal hydrolase 8 (USP8)	USP8	P40818	OID01200	Olink METABOLISM(v.3401)
Versican core protein (VCAN)	VCAN	P13611	OID01194	Olink METABOLISM(v.3401)
Vascular endothelial growth factor D (VEGFD)	VEGFD	O43915	OID00468	Olink CARDIOVASCULAR II(v.5003)
V-set and immunoglobulin domain-containing protein 2 (VSIG2)	VSIG2	Q96IQ7	OID00429	Olink CARDIOVASCULAR II(v.5003)
von Willebrand factor (vWF)	vWF	P04275	OID00651	Olink CARDIOVASCULAR III(v.6112)

Lymphotactin (XCL1)	XCL1	P47992	OID00433	Olink CARDIOVASCULAR II(v.5003)

Additional File Table S2. Change over time in reported daily non-fermented and fermented milk consumption in women and men.

	Change in reported intake	
	Non-fermented milk (mL/d; 95% CI)	Fermented milk (mL/d; 95% CI)
	Women, from 1987-1990 to 1997	
No new comorbidity	-86 (-88, -83)	76 (74, 79)
One new comorbidity	-80 (-87, -72)	73 (66, 80)
Two or more new comorbidities	-82 (-100, -63)	74 (55, 94)
No new MI	-85 (-87, -83)	76 (74, 78)
One new MI	-96 (-115, -76)	84 (64, 104)
	Women, from 1997 to 2009	
No new comorbidity	-1 (-4, 2)	31 (27, 36)
One new comorbidity	-8 (-15, -0)	32 (21, 42)
Two or more new comorbidities	-9 (-28, 10)	39 (15, 64)
No new MI	-2 (-5, 0)	32 (27, 36)
One new MI	-3 (-23, 17)	42 (18, 65)
	Men, from 1997 to 2009	
No new comorbidity	-32 (-37, -27)	42 (37, 47)
One new comorbidity	-40 (-51, -29)	42 (31, 53)
Two or more new comorbidities	-33 (-57, -8)	43 (15, 71)
No new MI	-34 (-38, -29)	42 (37, 46)
One new MI	-45 (-65, -24)	47 (26, 68)

Additional File Table S3. Non-fermented milk consumption and time to ischemic heart disease (IHD) in the Swedish Mammography Cohort and the Cohort of Swedish Men

	Categories of non-fermented milk intake				
	<1 glass/day<br><200 mL/day	1 to <2 glasses/day<br>200 to 399 mL/day	2 to <3 glasses/day<br>400 to 599 mL/day	≥3 glasses/day<br>≥600 mL/day	Continuous<br>per 200 ml
			Women		
Number of women with IHD	4493	2785	1637	619	9534
Person-years of follow-up	732,771	430,820	242,225	88,179	1,493,996
Rate/1000 person-years (95% CI)	6.1 (6.0-6.3)	6.5 (6.1-6.7)	6.8 (6.4-7.1)	7.0 (6.5-7.6)	
Age-adjusted HR (95% CI)	1.00 (reference)	1.06 (1.01, 1.12)<br>P=0.013	1.18 (1.11, 1.25)<br>P<0.001	1.37 (1.25, 1.49)<br>P<0.001	1.07 (1.05, 1.09)<br>P<0.001
Multivariable-adjusted HR (95% CI)*	1.00 (reference)	1.00 (0.95, 1.05)<br>P=0.93	1.07 (1.00, 1.14)<br>P=0.039	1.19 (1.08, 1.31)<br>P<0.001	1.04 (1.01, 1.06)<br>P=0.002
			Men		
Number of men with IHD	3688	1948	1419	1307	8362
Person-years of follow-up	324,067	158,804	107,119	94,624	684,613
Rate/1000 person-years (95% CI)	11.4 (11.0-11.8)	12.3 (11.7-12.8)	13.2 (12.6-14.0)	13.8 (13.1-14.6)	
Age-adjusted HR (95% CI)	1.00 (reference)	0.98 (0.93, 1.03)<br>P=0.42	1.07 (1.00, 1.13)<br>P=0.043	1.17 (1.10, 1.25)<br>P<0.001	1.03 (1.02, 1.05)<br>P<0.001

Multivariable-adjusted HR (95% CI)*	1.00 (reference)	0.95 (0.90, 1.01)<br>P=0.081	1.00 (0.94, 1.07)<br>P=0.98	1.04 (0.97, 1.12)<br>P=0.27	1.01 (0.99, 1.02)<br>P=0.24

HR: Hazard Ratio, CI: confidence interval

*The multivariable-adjusted hazard ratios were adjusted for age, educational level, living alone, leisure time exercise, walking/cycling, body mass index, height, total energy intake, fermented milk intake, cheese intake, alcohol consumption, fruits and vegetable intake, red meat intake, soft drinks and juice intake, total fat intake, saturated fat intake, smoking status, vitamin- and mineral supplements, baseline weighted Charlson comorbidity index, baseline CVD, and baseline diabetes mellitus.

Additional File Table S4. Fermented milk consumption and time to ischemic heart disease (IHD) in the Swedish Mammography Cohort and the Cohort of Swedish Men

	Categories of fermented milk intake				Continuous
	0 mL/day	1 to 199 mL/day	200 to 399 mL/day	≥400 mL/day	per 200 mL/day
			Women		
Number of women with IHD	2613	3828	2233	860	9534
Person-years of follow-up	336,465	684,500	360,900	112,131	1,493,996
Rate/1000 person-years (95% CI)	7.8 (7.5-8.1)	5.6 (5.4-5.8)	6.2 (5.9-6.4)	7.7 (7.2-8.2)	
Age-adjusted HR (95% CI)	1.00 (reference)	0.91 (0.87, 0.96)	0.83 (0.78, 0.87)	0.86 (0.80, 0.93)	0.94 (0.92, 0.97)
		P=<0.001	P<0.001	P<0.001	P<0.001
Multivariable-adjusted HR (95% CI)*	1.00 (reference)	0.96 (0.90, 1.03)	0.92 (0.85, 0.99)	0.94 (0.84, 1.05)	0.99 (0.96, 1.01)
		P=0.28	P=0.025	P=0.28	P=0.40
			Men		
Number of men with IHD	3165	1977	1861	1359	8362
Person-years of follow-up	237,959	181,381	159,397	105,877	684,613
Rate/1000 person-years (95% CI)	13,3 (12.8-13.89	10.9 (10.4-11.4)	11.7 (11.2-12.2)	12.8 (12.2-13.5)	
Age-adjusted HR (95% CI)	1.00 (reference)	0.92 (0.87, 0.97)	0.86 (0.86, 0.91)	0.91 (0.86, 0.97)	0.98 (0.96, 0.99)
		P=0.003	P<0.001	P=0.005	P=0.005

Multivariable-adjusted HR (95% CI)*	1.00 (reference)	0.98 (0.92, 1.04)<br>P=0.46	0.94 (0.88, 1.00)<br>P=0.03	0.97 (0.91, 1.04)<br>P=0.43	0.98 (0.97, 1.00)<br>P=0.079

HR: Hazard Ratio, CI: confidence interval

*The multivariable-adjusted hazard ratios were adjusted for age, educational level, living alone, leisure time exercise, walking/cycling, body mass index, height, total energy intake, non-fermented milk intake, cheese intake, alcohol consumption, fruits and vegetable intake, red meat intake, soft drinks and juice intake, total fat intake, saturated fat intake, smoking status, vitamin- and mineral supplements, baseline weighted Charlson comorbidity index, baseline CVD, and baseline diabetes mellitus.

Additional File Table S5. Baseline characteristics of the participants in the discovery and replication cohort with proteomics data.

Variable	Level	Discovery cohort	Replication cohort by sex	
			Women	Men
		N=5007	N=2572	N =4735
Age at baseline, mean (SD)	years	67.6 (6.8)	74.3 (3.7)	74.3 (6.0)
Sex, n (%)		5007 (100.0%)	2572 (100.0%)	4735 (100.0%)
Weight, mean (SD)	kg	69.6 (12.0)	70.3 (12.5)	82.3 (12.9)
Height, mean (SD)	cm	163.6 (6.1)	162.3 (6.1)	176.0 (6.6)
Body mass index, mean (SD)	kg/m^2	26.0 (4.3)	26.7 (4.6)	26.6 (3.7)
Energy intake, mean (SD)	kcal	1810 (542)	1858 (535)	2438 (704)
Charlson comorbidity index, n (%)	0	4265 (85.2%)	1835 (71.3%)	3005 (63.5%)
	1	470 (9.4%)	318 (12.4%)	809 (17.1%)
	2	272 (5.4%)	419 (16.3%)	921 (19.5%)
Smoking status, n (%)	Current	1090 (22.1%)	453 (19.8%)	923 (19.7%)
	Former	1332 (26.9%)	634 (27.7%)	1852 (39.5%)
	Never	2521 (51.0%)	1202 (52.5%)	1919 (40.9%)
Education level, n (%)	</= 9 years	1205 (24.0%)	687 (29.0%)	1103 (23.3%)
	10-12 years	1939 (38.6%)	1119 (47.3%)	2620 (55.2%)
	>12 years	1876 (37.4%)	559 (23.6%)	1020 (21.5%)
Self-rated health, n (%)	Very good	967 (21.7%)	516 (20.3%)	1016 (22.0%)
	Good	2477 (55.6%)	1426 (56.2%)	2701 (58.5%)
	Intermediate	931 (20.9%)	554 (21.8%)	838 (18.1%)
	Bad	73 (1.6%)	38 (1.5%)	60 (1.3%)
	Very bad	9 (0.2%)	2 (0.1%)	5 (0.1%)
Physical activity, n (%)	1	115 (2.7%)	69 (2.9%)	151 (3.4%)
	2	385 (9.2%)	210 (8.7%)	660 (14.6%)
	3	1612 (38.4%)	1033 (43.0%)	1629 (36.1%)
	4	1336 (31.8%)	779 (32.4%)	1241 (27.5%)
	5	492 (11.7%)	210 (8.7%)	502 (11.1%)
	6	255 (6.1%)	104 (4.3%)	324 (7.2%)

Additional File Table S6. Linear association proteomics results for both non-fermented and fermented milk intake in the discovery and replication cohort, also by sex.

Non-fermented milk

Protein	Discovery cohort, Female, Model A: Estimate (95% CI)	P-value	Discovery cohort, Female, Model B: Estimate (95% CI)	P-value	Replication cohort, All, Model A: Estimate (95% CI)	P-value	Replication cohort, All, Model B: Estimate (95% CI)	P-value
ACE2	0.0277 (0.0120, 0.0435)	0.001	0.0288 (0.0122, 0.0454)	0.001	0.0178 (0.0036, 0.0321)	0.014	0.0113 (-0.0039, 0.0265)	0.146
ACP6	-0.0027 (-0.0159, 0.0105)	0.691	-0.0064 (-0.0207, 0.0079)	0.381	-0.0016 (-0.0125, 0.0092)	0.767	-0.0047 (-0.0168, 0.0074)	0.443
ADAM-TS13	0.0004 (-0.0035, 0.0044)	0.831	0.0011 (-0.0032, 0.0053)	0.627	0.0007 (-0.0050, 0.0064)	0.803	0.0025 (-0.0039, 0.0089)	0.446
ADGRE2	-0.0002 (-0.0098, 0.0095)	0.975	-0.0092 (-0.0196, 0.0011)	0.079	0.0082 (0.0000, 0.0164)	0.050	-0.0020 (-0.0109, 0.0069)	0.657
ADGRG2	0.0053 (-0.0029, 0.0135)	0.205	0.0050 (-0.0036, 0.0135)	0.254	0.0040 (-0.0040, 0.0119)	0.328	0.0041 (-0.0045, 0.0127)	0.348
ADM	0.0006 (-0.0104, 0.0116)	0.914	-0.0023 (-0.0128, 0.0083)	0.674	0.0086 (-0.0014, 0.0186)	0.092	0.0011 (-0.0090, 0.0112)	0.835
AGRP	0.0050 (-0.0049, 0.0150)	0.324	0.0009 (-0.0099, 0.0117)	0.872	0.0069 (-0.0031, 0.0168)	0.178	0.0036 (-0.0075, 0.0147)	0.524
AHCY	0.0022 (-0.0090, 0.0135)	0.698	-0.0009 (-0.0131, 0.0113)	0.885	-0.0010 (-0.0137, 0.0117)	0.873	0.0039 (-0.0103, 0.0181)	0.589
ALCAM	0.0045 (-0.0026, 0.0117)	0.211	-0.0034 (-0.0111, 0.0042)	0.379	0.0152 (0.0087, 0.0217)	0.000	0.0054 (-0.0017, 0.0125)	0.138
ALDH1A1	0.0082 (-0.0043, 0.0207)	0.200	0.0069 (-0.0061, 0.0200)	0.296	0.0089 (-0.0023, 0.0200)	0.119	0.0077 (-0.0042, 0.0197)	0.205
AMBP	0.0062 (0.0012, 0.0111)	0.014	0.0042 (-0.0011, 0.0094)	0.121	0.0069 (0.0004, 0.0134)	0.038	0.0036 (-0.0037, 0.0108)	0.334
ANGPT1	-0.0080 (-0.0225, 0.0065)	0.279	-0.0033 (-0.0191, 0.0124)	0.681	0.0088 (-0.0053, 0.0230)	0.223	0.0057 (-0.0101, 0.0215)	0.478
ANGPT2	0.0055 (-0.0054, 0.0164)	0.321	0.0017 (-0.0099, 0.0132)	0.780	0.0132 (0.0032, 0.0232)	0.010	0.0079 (-0.0028, 0.0187)	0.149
ANGPTL1	0.0112 (0.0025, 0.0199)	0.012	0.0064 (-0.0030, 0.0157)	0.183	0.0013 (-0.0062, 0.0087)	0.742	-0.0052 (-0.0135, 0.0031)	0.219
ANGPTL7	-0.0002 (-0.0087, 0.0084)	0.969	-0.0041 (-0.0133, 0.0052)	0.390	0.0043 (-0.0038, 0.0124)	0.296	-0.0002 (-0.0092, 0.0088)	0.970
ANXA11	0.0174 (0.0013, 0.0334)	0.034	0.0211 (0.0037, 0.0385)	0.018	0.0033 (-0.0105, 0.0172)	0.636	-0.0055 (-0.0210, 0.0100)	0.485
ANXA4	0.0073 (0.0014, 0.0132)	0.016	0.0067 (0.0003, 0.0132)	0.040	0.0034 (-0.0031, 0.0099)	0.308	0.0040 (-0.0033, 0.0113)	0.288
AP-N	0.0017 (-0.0071, 0.0106)	0.700	0.0007 (-0.0089, 0.0104)	0.879	0.0048 (-0.0024, 0.0121)	0.189	0.0074 (-0.0007, 0.0155)	0.072
APEX1	0.0028 (-0.0125, 0.0181)	0.717	-0.0012 (-0.0177, 0.0152)	0.882	0.0026 (-0.0079, 0.0130)	0.630	-0.0093 (-0.0209, 0.0024)	0.118
APLP1	-0.0096 (-0.0235, 0.0044)	0.179	-0.0064 (-0.0209, 0.0081)	0.389	-0.0020 (-0.0138, 0.0098)	0.741	0.0010 (-0.0118, 0.0137)	0.879
ARG1	0.0134 (-0.0039, 0.0306)	0.130	0.0116 (-0.0072, 0.0304)	0.226	0.0010 (-0.0141, 0.0160)	0.899	-0.0103 (-0.0271, 0.0065)	0.228
AXL	0.0010 (-0.0083, 0.0103)	0.832	-0.0081 (-0.0180, 0.0019)	0.111	0.0110 (0.0030, 0.0191)	0.007	0.0013 (-0.0076, 0.0101)	0.782
AZU1	-0.0004 (-0.0222, 0.0215)	0.974	-0.0091 (-0.0327, 0.0146)	0.453	0.0137 (-0.0030, 0.0304)	0.109	-0.0078 (-0.0264, 0.0109)	0.414
BAG6	-0.0014 (-0.0082, 0.0053)	0.677	0.0005 (-0.0068, 0.0077)	0.903	0.0033 (-0.0036, 0.0102)	0.353	0.0010 (-0.0067, 0.0087)	0.803
BLM hydrolase	0.0056 (-0.0052, 0.0163)	0.312	0.0077 (-0.0039, 0.0194)	0.194	0.0118 (0.0014, 0.0223)	0.026	0.0090 (-0.0026, 0.0206)	0.129
BMP-6	-0.0024 (-0.0244, 0.0195)	0.830	-0.0161 (-0.0399, 0.0077)	0.186	0.0174 (0.0044, 0.0305)	0.009	0.0086 (-0.0060, 0.0232)	0.249
BNP	0.0102 (-0.0185, 0.0389)	0.487	0.0181 (-0.0128, 0.0491)	0.250	0.0045 (-0.0268, 0.0358)	0.780	-0.0027 (-0.0367, 0.0312)	0.874
BOC	0.0007 (-0.0060, 0.0074)	0.834	-0.0045 (-0.0117, 0.0027)	0.221	0.0086 (0.0012, 0.0160)	0.022	0.0058 (-0.0024, 0.0140)	0.167
CA13	0.0017 (-0.0114, 0.0149)	0.795	0.0043 (-0.0099, 0.0186)	0.550	0.0073 (-0.0060, 0.0206)	0.280	0.0023 (-0.0125, 0.0172)	0.757
CA5A	0.0516 (0.0242, 0.0789)	0.000	0.0461 (0.0172, 0.0750)	0.002	0.0051 (-0.0150, 0.0252)	0.620	-0.0054 (-0.0268, 0.0161)	0.623
CANT1	0.0030 (-0.0036, 0.0097)	0.367	0.0000 (-0.0071, 0.0071)	0.997	0.0010 (-0.0054, 0.0073)	0.758	-0.0052 (-0.0122, 0.0018)	0.143
CASP-3	-0.0051 (-0.0168, 0.0066)	0.395	-0.0037 (-0.0164, 0.0089)	0.564	0.0148 (0.0030, 0.0266)	0.014	0.0084 (-0.0047, 0.0215)	0.208
CCDC80	-0.0072 (-0.0214, 0.0070)	0.322	-0.0110 (-0.0263, 0.0043)	0.160	0.0048 (-0.0071, 0.0167)	0.428	-0.0032 (-0.0163, 0.0100)	0.636
CCL15	-0.0010 (-0.0157, 0.0138)	0.898	-0.0070 (-0.0229, 0.0089)	0.390	0.0113 (-0.0013, 0.0239)	0.079	-0.0053 (-0.0191, 0.0085)	0.455
CCL16	-0.0025 (-0.0178, 0.0127)	0.746	-0.0029 (-0.0190, 0.0132)	0.723	0.0142 (0.0011, 0.0273)	0.034	0.0073 (-0.0070, 0.0215)	0.316
CCL17	-0.0014 (-0.0183, 0.0156)	0.874	-0.0047 (-0.0230, 0.0135)	0.610	0.0046 (-0.0099, 0.0190)	0.538	-0.0049 (-0.0210, 0.0112)	0.553
CCL24	-0.0073 (-0.0331, 0.0186)	0.581	-0.0142 (-0.0424, 0.0139)	0.320	0.0107 (-0.0100, 0.0314)	0.309	0.0091 (-0.0140, 0.0323)	0.440
CCL3	0.0074 (-0.0107, 0.0255)	0.423	0.0002 (-0.0191, 0.0195)	0.985	0.0042 (-0.0102, 0.0185)	0.570	-0.0031 (-0.0188, 0.0127)	0.703
CD163	0.0155 (0.0016, 0.0294)	0.029	0.0044 (-0.0102, 0.0189)	0.555	0.0226 (0.0118, 0.0334)	0.000	0.0124 (0.0008, 0.0240)	0.036
CD164	0.0028 (-0.0039, 0.0094)	0.418	-0.0016 (-0.0088, 0.0056)	0.665	0.0025 (-0.0037, 0.0086)	0.432	-0.0042 (-0.0110, 0.0026)	0.226
CD1C	0.0004 (-0.0084, 0.0092)	0.931	-0.0046 (-0.0141, 0.0049)	0.346	0.0025 (-0.0057, 0.0106)	0.551	-0.0038 (-0.0129, 0.0052)	0.408
CD2AP	-0.0094 (-0.0205, 0.0017)	0.096	-0.0068 (-0.0187, 0.0052)	0.269	0.0103 (-0.0005, 0.0211)	0.062	0.0029 (-0.0091, 0.0150)	0.633
CD4	0.0066 (-0.0024, 0.0156)	0.149	0.0009 (-0.0087, 0.0106)	0.850	0.0095 (0.0008, 0.0181)	0.031	0.0058 (-0.0035, 0.0152)	0.222
CD40-L	-0.0062 (-0.0204, 0.0079)	0.386	-0.0044 (-0.0198, 0.0110)	0.574	0.0067 (-0.0074, 0.0207)	0.352	0.0049 (-0.0108, 0.0205)	0.543
CD79B	0.0018 (-0.0084, 0.0119)	0.732	-0.0108 (-0.0217, 0.0001)	0.051	0.0057 (-0.0050, 0.0163)	0.295	-0.0116 (-0.0233, 0.0001)	0.051
CD84	-0.0070 (-0.0155, 0.0015)	0.107	-0.0130 (-0.0222, -0.0038)	0.005	0.0100 (0.0012, 0.0187)	0.025	0.0013 (-0.0085, 0.0110)	0.798
CD93	-0.0025 (-0.0116, 0.0067)	0.598	-0.0132 (-0.0230, -0.0034)	0.008	0.0083 (0.0007, 0.0159)	0.031	-0.0038 (-0.0122, 0.0046)	0.376
CDH2	0.0035 (-0.0071, 0.0140)	0.518	-0.0070 (-0.0180, 0.0041)	0.217	0.0029 (-0.0072, 0.0131)	0.570	-0.0114 (-0.0223, -0.0005)	0.041
CDH5	0.0045 (-0.0045, 0.0136)	0.328	-0.0020 (-0.0118, 0.0078)	0.689	0.0165 (0.0080, 0.0250)	0.000	0.0077 (-0.0017, 0.0172)	0.110
CDHR5	-0.0019 (-0.0141, 0.0104)	0.765	-0.0151 (-0.0278, -0.0024)	0.020	0.0007 (-0.0091, 0.0104)	0.892	-0.0120 (-0.0227, -0.0013)	0.027
CEACAM8	0.0069 (-0.0101, 0.0238)	0.428	-0.0019 (-0.0201, 0.0164)	0.842	0.0115 (-0.0015, 0.0244)	0.084	-0.0071 (-0.0215, 0.0072)	0.328
CHI3L1	-0.0205 (-0.0421, 0.0011)	0.062	-0.0241 (-0.0470, -0.0012)	0.039	-0.0071 (-0.0268, 0.0126)	0.481	-0.0063 (-0.0273, 0.0148)	0.559
CHIT1	-0.0081 (-0.0488, 0.0326)	0.698	-0.0163 (-0.0604, 0.0279)	0.470	0.0289 (-0.0012, 0.0590)	0.060	0.0061 (-0.0275, 0.0397)	0.724
CHRDL2	0.0044 (-0.0106, 0.0194)	0.564	-0.0099 (-0.0260, 0.0063)	0.232	0.0126 (0.0013, 0.0240)	0.028	-0.0017 (-0.0143, 0.0108)	0.790
CLEC5A	0.0021 (-0.0068, 0.0109)	0.646	-0.0029 (-0.0123, 0.0065)	0.549	0.0038 (-0.0041, 0.0117)	0.344	-0.0095 (-0.0181, -0.0008)	0.031
CLMP	-0.0035 (-0.0169, 0.0098)	0.601	-0.0161 (-0.0293, -0.0028)	0.017	0.0076 (-0.0039, 0.0191)	0.195	-0.0106 (-0.0224, 0.0012)	0.077
CLSTN2	-0.0044 (-0.0145, 0.0056)	0.390	-0.0110 (-0.0218, -0.0002)	0.046	0.0072 (-0.0021, 0.0165)	0.128	0.0014 (-0.0089, 0.0116)	0.794
CLUL1	-0.0068 (-0.0205, 0.0069)	0.329	-0.0007 (-0.0153, 0.0140)	0.930	-0.0038 (-0.0157, 0.0080)	0.527	-0.0071 (-0.0203, 0.0060)	0.286
CNTN1	-0.0010 (-0.0090, 0.0071)	0.817	-0.0035 (-0.0121, 0.0052)	0.432	0.0063 (-0.0021, 0.0147)	0.144	0.0085 (-0.0009, 0.0178)	0.075
COL1A1	-0.0177 (-0.0294, -0.0060)	0.003	-0.0238 (-0.0363, -0.0112)	0.000	-0.0037 (-0.0120, 0.0046)	0.379	-0.0161 (-0.0252, -0.0069)	0.001
COMT	0.0023 (-0.0098, 0.0144)	0.707	0.0068 (-0.0062, 0.0197)	0.307	-0.0031 (-0.0135, 0.0073)	0.558	-0.0063 (-0.0177, 0.0052)	0.282
CPA1	-0.0107 (-0.0289, 0.0075)	0.250	0.0001 (-0.0196, 0.0198)	0.990	-0.0118 (-0.0288, 0.0052)	0.174	-0.0030 (-0.0219, 0.0159)	0.755
CPB1	-0.0088 (-0.0262, 0.0086)	0.322	0.0001 (-0.0188, 0.0190)	0.991	-0.0133 (-0.0296, 0.0030)	0.109	-0.0111 (-0.0292, 0.0070)	0.230
CRKL	-0.0026 (-0.0138, 0.0086)	0.648	-0.0013 (-0.0134, 0.0107)	0.828	0.0082 (-0.0038, 0.0201)	0.180	0.0000 (-0.0132, 0.0133)	0.997
CSTB	0.0143 (-0.0004, 0.0290)	0.057	0.0050 (-0.0101, 0.0202)	0.517	0.0183 (0.0062, 0.0303)	0.003	-0.0027 (-0.0155, 0.0102)	0.682
CTRC	0.0169 (-0.0018, 0.0356)	0.076	0.0211 (0.0010, 0.0412)	0.040	0.0083 (-0.0089, 0.0256)	0.344	0.0106 (-0.0085, 0.0298)	0.277
CTSD	-0.0003 (-0.0128, 0.0123)	0.967	-0.0021 (-0.0149, 0.0108)	0.754	0.0089 (-0.0032, 0.0210)	0.149	0.0050 (-0.0078, 0.0178)	0.444
CTSH	0.0049 (-0.0113, 0.0211)	0.552	-0.0008 (-0.0183, 0.0168)	0.930	0.0022 (-0.0134, 0.0178)	0.787	-0.0125 (-0.0300, 0.0049)	0.160
CTSL1	-0.0018 (-0.0111, 0.0076)	0.710	-0.0101 (-0.0202, -0.0001)	0.047	0.0089 (0.0003, 0.0175)	0.042	0.0023 (-0.0072, 0.0117)	0.639
CTSO	0.0094 (0.0007, 0.0181)	0.034	0.0027 (-0.0063, 0.0116)	0.559	0.0067 (-0.0012, 0.0146)	0.096	-0.0032 (-0.0117, 0.0053)	0.459
CTSZ	0.0024 (-0.0083, 0.0131)	0.662	-0.0112 (-0.0224, -0.0001)	0.048	0.0138 (0.0043, 0.0232)	0.004	-0.0038 (-0.0141, 0.0064)	0.463
CXCL1	-0.0090 (-0.0237, 0.0058)	0.233	-0.0137 (-0.0297, 0.0022)	0.092	0.0039 (-0.0101, 0.0178)	0.588	0.0008 (-0.0147, 0.0164)	0.917

RARRES2	0.0035 (-0.0022, 0.0093)	0.229	0.0018 (-0.0039, 0.0075)	0.539	0.0054 (-0.0001, 0.0109)	0.055	0.0027 (-0.0032, 0.0085)	0.369	-0.0031 (-0.0173, 0.0111)	0.667	-0.0076 (-0.0221, 0.0070)	0.309	0.0068 (0.0008, 0.0128)	0.026	0.0043 (-0.0022, 0.0108)	0.197
REG4	0.0001 (-0.0163, 0.0166)	0.989	0.0020 (-0.0155, 0.0194)	0.826	0.0085 (-0.0058, 0.0227)	0.245	0.0025 (-0.0130, 0.0179)	0.753	0.0116 (-0.0260, 0.0492)	0.545	0.0069 (-0.0323, 0.0460)	0.731	0.0081 (-0.0073, 0.0236)	0.303	0.0012 (-0.0158, 0.0183)	0.887
REN	0.0180 (-0.0033, 0.0392)	0.097	0.0221 (-0.0001, 0.0444)	0.051	0.0130 (-0.0063, 0.0323)	0.186	0.0259 (0.0057, 0.0461)	0.012	-0.0104 (-0.0605, 0.0396)	0.682	-0.0065 (-0.0569, 0.0438)	0.800	0.0168 (-0.0043, 0.0378)	0.118	0.0325 (0.0100, 0.0549)	0.005
RETN	-0.0006 (-0.0141, 0.0130)	0.936	-0.0177 (-0.0321, -0.0033)	0.016	0.0096 (-0.0014, 0.0207)	0.088	-0.0146 (-0.0266, -0.0025)	0.018	-0.0200 (-0.0492, 0.0092)	0.180	-0.0555 (-0.0859, -0.0251)	0.000	0.0150 (0.0030, 0.0269)	0.014	-0.0065 (-0.0199, 0.0069)	0.341
RNASE3	0.0225 (-0.0100, 0.0550)	0.175	0.0102 (-0.0250, 0.0454)	0.569	0.0193 (-0.0035, 0.0421)	0.098	-0.0051 (-0.0306, 0.0203)	0.692	0.0416 (-0.0188, 0.1019)	0.177	0.0200 (-0.0447, 0.0847)	0.544	0.0160 (-0.0087, 0.0406)	0.204	-0.0102 (-0.0381, 0.0178)	0.476
ROR1	-0.0010 (-0.0109, 0.0089)	0.847	-0.0120 (-0.0226, -0.0014)	0.026	0.0055 (-0.0030, 0.0139)	0.204	-0.0074 (-0.0167, 0.0020)	0.121	0.0005 (-0.0217, 0.0227)	0.964	-0.0149 (-0.0385, 0.0087)	0.217	0.0064 (-0.0027, 0.0156)	0.170	-0.0069 (-0.0172, 0.0035)	0.193
RTN4R	0.0089 (-0.0002, 0.0179)	0.055	-0.0027 (-0.0118, 0.0063)	0.551	0.0194 (0.0105, 0.0283)	0.000	0.0070 (-0.0021, 0.0160)	0.132	0.0187 (-0.0056, 0.0431)	0.132	-0.0022 (-0.0262, 0.0219)	0.859	0.0197 (0.0103, 0.0291)	0.000	0.0074 (-0.0023, 0.0171)	0.134
S100P	0.0123 (0.0046, 0.0199)	0.002	0.0079 (-0.0003, 0.0161)	0.060	0.0062 (-0.0037, 0.0161)	0.217	-0.0011 (-0.0121, 0.0100)	0.849	0.0020 (-0.0256, 0.0297)	0.886	-0.0121 (-0.0418, 0.0177)	0.427	0.0069 (-0.0033, 0.0171)	0.185	0.0009 (-0.0107, 0.0126)	0.873
SCF	-0.0135 (-0.0229, -0.0042)	0.005	-0.0216 (-0.0315, -0.0116)	0.000	0.0012 (-0.0071, 0.0096)	0.771	-0.0032 (-0.0124, 0.0060)	0.497	-0.0105 (-0.0325, 0.0114)	0.345	-0.0223 (-0.0457, 0.0010)	0.060	0.0030 (-0.0059, 0.0120)	0.508	0.0002 (-0.0099, 0.0104)	0.962
SCGB3A2	-0.0109 (-0.0337, 0.0119)	0.350	0.0060 (-0.0177, 0.0297)	0.619	-0.0120 (-0.0286, 0.0047)	0.159	-0.0115 (-0.0296, 0.0066)	0.212	-0.0284 (-0.0772, 0.0203)	0.253	-0.0222 (-0.0734, 0.0289)	0.394	-0.0090 (-0.0259, 0.0078)	0.292	-0.0078 (-0.0264, 0.0109)	0.414
SDC4	-0.0021 (-0.0171, 0.0130)	0.789	0.0006 (-0.0157, 0.0170)	0.940	0.0071 (-0.0064, 0.0206)	0.303	0.0025 (-0.0127, 0.0176)	0.748	-0.0089 (-0.0457, 0.0280)	0.637	-0.0171 (-0.0569, 0.0226)	0.398	0.0098 (-0.0045, 0.0242)	0.180	0.0058 (-0.0105, 0.0222)	0.484
SELE	0.0037 (-0.0127, 0.0201)	0.661	-0.0042 (-0.0215, 0.0131)	0.634	0.0215 (0.0078, 0.0353)	0.002	0.0122 (-0.0027, 0.0271)	0.108	0.0424 (0.0060, 0.0788)	0.023	0.0219 (-0.0160, 0.0599)	0.258	0.0181 (0.0033, 0.0330)	0.017	0.0110 (-0.0053, 0.0274)	0.186
SELP	0.0063 (-0.0064, 0.0190)	0.328	0.0011 (-0.0127, 0.0148)	0.880	0.0141 (0.0033, 0.0249)	0.011	0.0044 (-0.0076, 0.0164)	0.474	0.0207 (-0.0089, 0.0503)	0.171	0.0094 (-0.0224, 0.0412)	0.561	0.0130 (0.0016, 0.0245)	0.026	0.0040 (-0.0089, 0.0170)	0.541
SEMA3E	0.0022 (-0.0050, 0.0094)	0.552	-0.0048 (-0.0123, 0.0028)	0.214	0.0014 (-0.0055, 0.0084)	0.683	-0.0082 (-0.0157, -0.0007)	0.033	-0.0113 (-0.0298, 0.0071)	0.227	-0.0219 (-0.0408, -0.0029)	0.024	0.0038 (-0.0036, 0.0113)	0.314	-0.0057 (-0.0140, 0.0025)	0.175
SERPINA12	0.0310 (0.0010, 0.0610)	0.043	0.0321 (-0.0005, 0.0646)	0.053	0.0434 (0.0219, 0.0648)	0.000	0.0409 (0.0171, 0.0647)	0.001	0.0865 (0.0295, 0.1434)	0.003	0.0706 (0.0094, 0.1319)	0.024	0.0360 (0.0130, 0.0591)	0.002	0.0364 (0.0104, 0.0624)	0.006
SERPINB6	0.0038 (-0.0048, 0.0123)	0.386	0.0024 (-0.0069, 0.0116)	0.614	0.0067 (-0.0012, 0.0145)	0.097	-0.0038 (-0.0125, 0.0049)	0.397	-0.0087 (-0.0299, 0.0126)	0.424	-0.0214 (-0.0441, 0.0013)	0.065	0.0096 (0.0013, 0.0179)	0.024	-0.0004 (-0.0098, 0.0090)	0.938
SERPINB8	0.0118 (-0.0030, 0.0266)	0.118	0.0040 (-0.0120, 0.0200)	0.621	0.0021 (-0.0105, 0.0147)	0.747	-0.0136 (-0.0276, 0.0004)	0.056	-0.0046 (-0.0362, 0.0271)	0.777	-0.0238 (-0.0576, 0.0100)	0.168	0.0038 (-0.0101, 0.0177)	0.593	-0.0123 (-0.0280, 0.0035)	0.127
SHPS-1	-0.0057 (-0.0165, 0.0050)	0.298	-0.0106 (-0.0222, 0.0011)	0.075	0.0157 (0.0060, 0.0254)	0.002	0.0048 (-0.0060, 0.0155)	0.388	0.0250 (-0.0012, 0.0513)	0.062	0.0155 (-0.0126, 0.0436)	0.279	0.0146 (0.0042, 0.0249)	0.006	0.0026 (-0.0091, 0.0143)	0.660
SIGLEC7	0.0095 (0.0014, 0.0175)	0.022	0.0075 (-0.0009, 0.0160)	0.081	0.0083 (0.0008, 0.0158)	0.029	0.0060 (-0.0021, 0.0141)	0.145	0.0057 (-0.0143, 0.0256)	0.577	0.0052 (-0.0155, 0.0260)	0.621	0.0089 (0.0009, 0.0169)	0.029	0.0066 (-0.0023, 0.0155)	0.144
SLAMF7	-0.0024 (-0.0204, 0.0155)	0.789	-0.0003 (-0.0198, 0.0191)	0.972	0.0099 (-0.0065, 0.0263)	0.236	0.0053 (-0.0130, 0.0236)	0.570	-0.0078 (-0.0502, 0.0347)	0.720	-0.0089 (-0.0546, 0.0368)	0.702	0.0131 (-0.0047, 0.0310)	0.150	0.0056 (-0.0147, 0.0259)	0.591
SNAP23	-0.0064 (-0.0173, 0.0045)	0.250	-0.0041 (-0.0159, 0.0077)	0.493	0.0056 (-0.0034, 0.0147)	0.224	0.0021 (-0.0080, 0.0122)	0.683	0.0015 (-0.0232, 0.0262)	0.903	0.0033 (-0.0232, 0.0298)	0.806	0.0065 (-0.0031, 0.0161)	0.186	0.0011 (-0.0098, 0.0120)	0.847
SOD2	0.0006 (-0.0048, 0.0060)	0.827	0.0017 (-0.0041, 0.0075)	0.566	0.0014 (-0.0037, 0.0065)	0.603	0.0033 (-0.0024, 0.0090)	0.261	0.0008 (-0.0150, 0.0166)	0.924	0.0033 (-0.0138, 0.0203)	0.706	0.0013 (-0.0036, 0.0062)	0.605	0.0025 (-0.0030, 0.0081)	0.375
SORT1	-0.0106 (-0.0169, -0.0044)	0.001	-0.0083 (-0.0151, -0.0015)	0.016	0.0004 (-0.0065, 0.0073)	0.907	0.0013 (-0.0063, 0.0090)	0.732	-0.0118 (-0.0314, 0.0079)	0.241	-0.0097 (-0.0309, 0.0114)	0.367	0.0024 (-0.0047, 0.0096)	0.504	0.0029 (-0.0052, 0.0109)	0.486
SOST	-0.0038 (-0.0149, 0.0073)	0.504	-0.0048 (-0.0168, 0.0072)	0.435	0.0121 (0.0020, 0.0222)	0.019	0.0064 (-0.0047, 0.0175)	0.260	0.0217 (-0.0045, 0.0478)	0.104	0.0206 (-0.0072, 0.0485)	0.146	0.0108 (-0.0002, 0.0218)	0.054	0.0038 (-0.0086, 0.0161)	0.551
SPON1	0.0005 (-0.0048, 0.0059)	0.843	-0.0008 (-0.0067, 0.0050)	0.775	0.0063 (-0.0006, 0.0132)	0.074	0.0008 (-0.0068, 0.0084)	0.843	-0.0048 (-0.0227, 0.0132)	0.604	-0.0086 (-0.0277, 0.0105)	0.379	0.0084 (0.0009, 0.0159)	0.028	0.0014 (-0.0070, 0.0099)	0.741
SPON2	-0.0012 (-0.0054, 0.0030)	0.570	-0.0048 (-0.0092, -0.0004)	0.033	0.0034 (-0.0021, 0.0090)	0.225	-0.0009 (-0.0071, 0.0053)	0.775	0.0036 (-0.0132, 0.0204)	0.674	-0.0012 (-0.0192, 0.0168)	0.894	0.0034 (-0.0021, 0.0088)	0.230	-0.0014 (-0.0076, 0.0047)	0.649
SRC	-0.0111 (-0.0208, -0.0014)	0.026	-0.0061 (-0.0166, 0.0045)	0.261	0.0085 (-0.0031, 0.0200)	0.150	0.0043 (-0.0085, 0.0171)	0.507	-0.0066 (-0.0387, 0.0256)	0.688	-0.0103 (-0.0447, 0.0242)	0.559	0.0109 (-0.0011, 0.0229)	0.076	0.0066 (-0.0070, 0.0202)	0.343
SSC4D	0.0716 (0.0377, 0.1055)	0.000	0.0456 (0.0124, 0.0788)	0.007	0.0628 (0.0324, 0.0931)	0.000	0.0066 (-0.0248, 0.0380)	0.678	0.0189 (-0.0536, 0.0915)	0.609	-0.0492 (-0.1196, 0.0211)	0.170	0.0703 (0.0359, 0.1046)	0.000	0.0140 (-0.0225, 0.0505)	0.452
ST2	-0.0023 (-0.0136, 0.0091)	0.695	-0.0018 (-0.0141, 0.0104)	0.770	0.0055 (-0.0058, 0.0168)	0.339	0.0118 (-0.0007, 0.0242)	0.064	-0.0192 (-0.0480, 0.0096)	0.191	-0.0121 (-0.0425, 0.0183)	0.434	0.0099 (-0.0024, 0.0222)	0.116	0.0155 (0.0016, 0.0295)	0.029
STK4	-0.0076 (-0.0215, 0.0064)	0.288	-0.0066 (-0.0217, 0.0085)	0.393	0.0049 (-0.0093, 0.0192)	0.497	0.0011 (-0.0148, 0.0171)	0.889	0.0043 (-0.0332, 0.0418)	0.823	0.0021 (-0.0383, 0.0425)	0.920	0.0050 (-0.0105, 0.0204)	0.530	-0.0011 (-0.0187, 0.0165)	0.903
SUMF2	0.0293 (0.0112, 0.0475)	0.002	0.0120 (-0.0072, 0.0313)	0.220	0.0134 (-0.0011, 0.0278)	0.070	-0.0183 (-0.0340, -0.0026)	0.023	0.0314 (-0.0077, 0.0704)	0.115	0.0039 (-0.0364, 0.0441)	0.850	0.0106 (-0.0049, 0.0260)	0.180	-0.0236 (-0.0408, -0.0064)	0.007
TF	0.0030 (-0.0053, 0.0113)	0.476	-0.0002 (-0.0092, 0.0088)	0.960	0.0116 (0.0040, 0.0192)	0.003	0.0050 (-0.0035, 0.0135)	0.253	-0.0007 (-0.0219, 0.0204)	0.945	-0.0076 (-0.0303, 0.0151)	0.513	0.0135 (0.0055, 0.0216)	0.001	0.0069 (-0.0022, 0.0159)	0.138
TFF2	0.0127 (-0.0043, 0.0297)	0.143	0.0122 (-0.0056, 0.0300)	0.180	-0.0001 (-0.0145, 0.0142)	0.985	0.0012 (-0.0142, 0.0166)	0.880	-0.0218 (-0.0594, 0.0158)	0.256	-0.0268 (-0.0660, 0.0124)	0.180	0.0038 (-0.0117, 0.0194)	0.627	0.0063 (-0.0106, 0.0233)	0.465
TFF3	-0.0050 (-0.0180, 0.0079)	0.446	-0.0049 (-0.0188, 0.0090)	0.493	0.0032 (-0.0072, 0.0136)	0.543	-0.0115 (-0.0226, -0.0003)	0.043	-0.0085 (-0.0357, 0.0187)	0.541	-0.0260 (-0.0541, 0.0021)	0.070	0.0055 (-0.0058, 0.0168)	0.343	-0.0095 (-0.0219, 0.0028)	0.128
TFPI	-0.0003 (-0.0095, 0.0090)	0.956	-0.0043 (-0.0143, 0.0058)	0.405	0.0049 (-0.0035, 0.0133)	0.254	0.0047 (-0.0046, 0.0139)	0.322	-0.0280 (-0.0501, -0.0058)	0.013	-0.0247 (-0.0485, -0.0009)	0.042	0.0105 (0.0015, 0.0195)	0.023	0.0084 (-0.0017, 0.0185)	0.102
TGM2	0.0118 (-0.0024, 0.0259)	0.103	0.0063 (-0.0091, 0.0216)	0.423	0.0087 (-0.0026, 0.0200)	0.130	0.0041 (-0.0083, 0.0166)	0.514	-0.0006 (-0.0345, 0.0333)	0.972	-0.0052 (-0.0414, 0.0311)	0.780	0.0100 (-0.0010, 0.0211)	0.074	0.0036 (-0.0088, 0.0160)	0.567
THBS2	-0.0026 (-0.0073, 0.0021)	0.282	-0.0051 (-0.0102, 0.0000)	0.052	0.0024 (-0.0040, 0.0088)	0.456	0.0008 (-0.0063, 0.0079)	0.827	0.0041 (-0.0142, 0.0225)	0.659	0.0048 (-0.0149, 0.0245)	0.634	0.0020 (-0.0045, 0.0086)	0.543	-0.0015 (-0.0088, 0.0059)	0.697
THOP1	0.0075 (-0.0008, 0.0157)	0.076	0.0046 (-0.0042, 0.0134)	0.307	0.0096 (0.0018, 0.0174)	0.016	0.0023 (-0.0063, 0.0109)	0.605	0.0045 (-0.0154, 0.0244)	0.655	0.0047 (-0.0164, 0.0258)	0.662	0.0107 (0.0022, 0.0193)	0.014	0.0009 (-0.0087, 0.0106)	0.851
THPO	-0.0008 (-0.0084, 0.0069)	0.844	0.0007 (-0.0076, 0.0090)	0.867	0.0014 (-0.0075, 0.0103)	0.750	0.0000 (-0.0099, 0.0099)	0.999	-0.0280 (-0.0518, -0.0043)	0.021	-0.0283 (-0.0538, -0.0028)	0.030	0.0063 (-0.0032, 0.0158)	0.193	0.0046 (-0.0062, 0.0154)	0.408
TIE2	-0.0023 (-0.0085, 0.0039)	0.463	-0.0044 (-0.0111, 0.0023)	0.201	0.0060 (-0.0008, 0.0128)	0.081	0.0057 (-0.0019, 0.0133)	0.140	0.0113 (-0.0074, 0.0300)	0.236	0.0102 (-0.0099, 0.0303)	0.321	0.0051 (-0.0021, 0.0122)	0.166	0.0042 (-0.0039, 0.0123)	0.311
TIMP4	-0.0105 (-0.0232, 0.0022)	0.105	-0.0100 (-0.0237, 0.0036)	0.149	0.0112 (0.0007, 0.0217)	0.037	0.0072 (-0.0042, 0.0187)	0.217	0.0029 (-0.0258, 0.0316)	0.843	0.0013 (-0.0288, 0.0315)	0.930	0.0128 (0.0017, 0.0240)	0.024	0.0074 (-0.0050, 0.0199)	0.240
TINAGL1	0.0049 (-0.0015, 0.0113)	0.130	-0.0005 (-0.0073, 0.0064)	0.893	0.0028 (-0.0032, 0.0088)	0.360	-0.0019 (-0.0085, 0.0047)	0.576	-0.0005 (-0.0164, 0.0153)	0.950	-0.0047 (-0.0215, 0.0122)	0.589	0.0035 (-0.0030, 0.0099)	0.291	-0.0018 (-0.0091, 0.0055)	0.623
TLT-2	-0.0032 (-0.0128, 0.0065)	0.517	-0.0140 (-0.0243, -0.0037)	0.008	0.0039 (-0.0072, 0.0151)	0.487	-0.0139 (-0.0262, -0.0016)	0.026	-0.0127 (-0.0414, 0.0160)	0.386	-0.0427 (-0.0729, -0.0124)	0.006	0.0071 (-0.0050, 0.0193)	0.251	-0.0064 (-0.0201, 0.0073)	0.361
TM	0.0004 (-0.0079, 0.0087)	0.928	-0.0082 (-0.0171, 0.0007)	0.072	0.0121 (0.0038, 0.0205)	0.005	0.0009 (-0.0084, 0.0103)	0.844	0.0047 (-0.0175, 0.0268)	0.679	-0.0099 (-0.0335, 0.0137)	0.409	0.0133 (0.0043, 0.0223)	0.004	0.0034 (-0.0068, 0.0137)	0.512
TNF-R1	-0.0003 (-0.0105, 0.0099)	0.958	-0.0107 (-0.0213, -0.0001)	0.049	0.0137 (0.0044, 0.0231)	0.004	-0.0040 (-0.0140, 0.0059)	0.426	-0.0131 (-0.0383, 0.0122)	0.310	-0.0354 (-0.0607, -0.0100)	0.006	0.0185 (0.0085, 0.0285)	0.000	0.0016 (-0.0093, 0.0126)	0.767
TNF-R2	0.0003 (-0.0109, 0.0114)	0.962	-0.0120 (-0.0237, -0.0002)	0.046	0.0193 (0.0081, 0.0304)	0.001	0.0009 (-0.0112, 0.0131)	0.881	0.0178 (-0.0116, 0.0472)	0.235	-0.0003 (-0.0308, 0.0303)	0.986	0.0199 (0.0079, 0.0320)	0.001	0.0015 (-0.0119, 0.0149)	0.825
TNFRSF10A	0.0066 (-0.0030, 0.0163)	0.175	0.0053 (-0.0048, 0.0154)	0.301	0.0109 (0.0016, 0.0201)	0.022	0.0041 (-0.0058, 0.0139)	0.422	0.0165 (-0.0078, 0.0408)	0.183	0.0037 (-0.0212, 0.0287)	0.770	0.0100 (-0.0001, 0.0200)	0.051	0.0032 (-0.0078, 0.0141)	0.572
TNFRSF10C	-0.0006 (-0.0155, 0.0144)	0.942	-0.0085 (-0.0246, 0.0075)	0.298	0.0095 (-0.0026, 0.0217)	0.125	-0.0084 (-0.0219, 0.0051)	0.222	-0.0024 (-0.0346, 0.0298)	0.883	-0.0316 (-0.0658, 0.0025)	0.069	0.0118 (-0.0013, 0.0249)	0.078	-0.0021 (-0.0170, 0.0127)	0.777
TNFRSF11A	-0.0014 (-0.0128, 0.0100)	0.810	-0.0097 (-0.0214, 0.0019)	0.101	0.0162 (0.0053, 0.0272)	0.004	-0.0025 (-0.0139, 0.0089)	0.668	-0.0090 (-0.0390, 0.0209)	0.556	-0.0338 (-0.0638, -0.0037)	0.028	0.0208 (0.0092, 0.0324)	0.000	0.0032 (-0.0091, 0.0155)	0.611
TNFRSF13B	0.0063 (-0.0042, 0.0168)	0.242	0.0012 (-0.0102, 0.0125)	0.841	0.0044 (-0.0069, 0.0157)	0.448	-0.0042 (-0.0167, 0.0083)	0.511	-0.0207 (-0.0485, 0.0070)	0.143	-0.0417 (-0.0713, -0.0121)	0.006	0.0087 (-0.0039, 0.0213)	0.176	0.0030 (-0.0112, 0.0173)	0.678
TNFRSF14	-0.0018 (-0.0112, 0.0076)	0.706	-0.0131 (-0.0231, -0.0032)	0.009	0.0118 (0.0028, 0.0208)	0.010	-0.0046 (-0.0143, 0.0051)	0.355	-0.0184 (-0.0422, 0.0054)	0.130	-0.0402 (-0.0646, -0.0158)	0.001	0.0172 (0.0074, 0.0269)	0.001	0.0023 (-0.0084, 0.0130)	0.678
TNFSF13B	-0.0012 (-0.0108, 0.0085)	0.812	-0.0059 (-0.0163, 0.0044)	0.261	0.0112 (0.0019, 0.0204)	0.019	0.0035 (-0.0067, 0.0138)	0.499	0.0027 (-0.0219, 0.0274)	0.828	-0.0037 (-0.0299, 0.0225)	0.783	0.0130 (0.0030, 0.0229)	0.011	0.0052 (-0.0060, 0.0165)	0.362
TR	0.0024 (-0.0107, 0.0155)	0.723	-0.0106 (-0.0246, 0.0034)	0.139	0.0223 (0.0097, 0.0349)	0.001	0.0124 (-0.0014, 0.0263)	0.077	-0.0041 (-0.0375, 0.0293)	0.809	-0.0199 (-0.0552, 0.0154)	0.270	0.0267 (0.0132, 0.0403)	0.000	0.0183 (0.0032, 0.0335)	0.018
TR-AP	-0.0034 (-0.0132, 0.0064)	0.495	-0.0032 (-0.0138, 0.0073)	0.547	0.0001 (-0.0087, 0.0088)	0.985	-0.0001 (-0.0098, 0.0096)	0.980	-0.0069 (-0.0294, 0.0155)	0.544	-0.0051 (-0.0292, 0.0189)	0.675	0.0016 (-0.0080, 0.0112)	0.745	0.0005 (-0.0103, 0.0113)	0.934
TRAIL-R2	0.0000 (-0.0105, 0.0106)	0.996	-0.0083 (-0.0193, 0.0027)	0.139	0.0152 (0.0032, 0.0273)	0.013	-0.0008 (-0.0138, 0.0122)	0.904	-0.0001 (-0.0322, 0.0321)	0.998	-0.0166 (-0.0500, 0.0168)	0.331	0.0179 (0.0050, 0.0308)	0.007	0.0022 (-0.0120, 0.0164)	0.764
TSHB	-0.0040 (-0.0304, 0.0224)	0.767	-0.0033 (-0.0319, 0.0253)	0.821	-0.0001 (-0.0193, 0.0192)	0.993	0.0074 (-0.0141, 0.0289)	0.499	-0.0325 (-0.0901, 0.0251)	0.269	-0.0275 (-0.0893, 0.0344)	0.384	0.0056 (-0.0135, 0.0247)	0.564	0.0110 (-0.0108, 0.0328)	0.322
TXNDC5	0.0083 (-0.0021, 0.0187)	0.118	0.0031 (-0.0082, 0.0143)	0.593	0.0088 (-0.0017, 0.0193)	0.102	-0.0048 (-0.0162, 0.0066)	0.409	-0.0139 (-0.0397, 0.0120)	0.292	-0.0312 (-0.0582, -0.0043)	0.023	0.0127 (0.0010, 0.0245)	0.033	-0.0011 (-0.0141, 0.0120)	0.869
TYMP	0.0060 (-0.0130, 0.0249)	0.537	0.0038 (-0.0166, 0.0243)	0.712	0.0065 (-0.0104, 0.0234)	0.449	-0.0010 (-0.0197, 0.0177)	0.920	-0.0375 (-0.0817, 0.0067)	0.096	-0.0490 (-0.0961, -0.0019)	0.041	0.0145 (-0.0037, 0.0327)	0.120	0.0080 (-0.0126, 0.0286)	0.448
TYRO3	0.0005 (-0.0067, 0.0078)	0.883	-0.0033 (-0.0111, 0.0045)	0.404	0.0070 (-0.0003, 0.0143)	0.061	0.0048 (-0.0033, 0.0129)	0.246	0.0133 (-0.0066, 0.0332)	0.189	0.0096 (-0.0115, 0.0308)	0.372	0.0061 (-0.0017, 0.0139)	0.125	0.0040 (-0.0048, 0.0129)	0.370
U-PAR	0.0074 (-0.0023, 0.0172)	0.136	-0.0012 (-0.0113, 0.0090)	0.822	0.0164 (0.0076, 0.0252)	0.000	0.0027 (-0.0067, 0.0121)	0.573	-0.0080 (-0.0308, 0.0148)	0.492	-0.0228 (-0.0463, 0.0006)	0.056	0.0208 (0.0112, 0.0304)	0.000	0.0064 (-0.0041, 0.0169)	0.230
USP8	0.0127 (0.0047, 0.0208)	0.002	0.0105 (0.0018, 0.0192)	0.018	0.0061 (-0.0017, 0.0139)	0.127	0.0049 (-0.0039, 0.0136)	0.274	0.0106 (-0.0110, 0.0322)	0.336	0.0133 (-0.0100, 0.0365)	0.263	0.0052 (-0.0030, 0.0133)	0.215	0.0018 (-0.0075, 0.0111)	0.699
VCAN	-0.0067 (-0.0158, 0.0025)	0.152	-0.0073 (-0.0171, 0.0025)	0.145	-0.0042 (-0.0129, 0.0046)	0.352	-0.0081 (-0.0178, 0.0016)	0.102	-0.0067 (-0.0297, 0.0163)	0.568	-0.0051 (-0.0296, 0.0194)	0.684	-0.0036 (-0.0131, 0.0059)	0.457	-0.0109 (-0.0216, -0.0001)	0.047
VEGFD	-0.0051 (-0.0158, 0.0055)	0.345	-0.0008 (-0.0120, 0.0104)	0.892	-0.0061 (-0.0168, 0.0047)	0.270	-0.0014 (-0.0133, 0.0105)	0.821	-0.0039 (-0.0305, 0.0227)	0.772	0.0062 (-0.0221, 0.0345)	0.667	-0.0065 (-0.0185, 0.0055)	0.286	-0.0038 (-0.0174, 0.0097)	0.578
VSIG2	0.0185 (0.0043, 0.0326)	0.010	0.0170 (0.0019, 0.0321)	0.028	0.0168 (0.0040, 0.0296)	0.010	0.0087 (-0.0051, 0.0225)	0.216	-0.0034 (-0.0380, 0.0312)	0.846	-0.0206 (-0.0569, 0.0157)	0.266	0.0202 (0.0066, 0.0339)	0.004	0.0133 (-0.0016, 0.0282)	0.081
XCL1	-0.0129 (-0.0285, 0.0027)	0.105	-0.0169 (-0.0337, -0.0001)	0.049	0.0126 (-0.0008, 0.0260)	0.066	0.0032 (-0.0115, 0.0180)	0.668	0.0138 (-0.0220, 0.0496)	0.450	0.0012 (-0.0368, 0.0392)	0.951	0.0123 (-0.0020, 0.0267)	0.093	0.0026 (-0.0136, 0.0188)	0.750
hOSCAR	0.0029 (-0.0032, 0.0090)	0.355	-0.0021 (-0.0086, 0.0045)	0.539	0.0075 (0.0008, 0.0143)	0.029	0.0011 (-0.0064, 0.0086)	0.769	0.0076 (-0.0115, 0.0266)	0.436	0.0000 (-0.0204, 0.0203)	0.997	0.0074 (0.0005, 0.0144)	0.037	0.0017 (-0.0062, 0.0096)	0.676
t-PA	-0.0037 (-0.0210, 0.0135)	0.673	-0.0037 (-0.0211, 0.0137)	0.675	0.0168 (0.0041, 0.0295)	0.009	0.0144 (0.0014, 0.0274)	0.030	0.0112 (-0.0222, 0.0446)	0.511	0.0136 (-0.0191, 0.0464)	0.415	0.0180 (0.0043, 0.0317)	0.010	0.0131 (-0.0013, 0.0274)	0.074
uPA	0.0017 (-0.0076, 0.0110)	0.722	-0.0034 (-0.0135, 0.0066)	0.505	0.0125 (0.0044, 0.0206)	0.003	0.0073 (-0.0017, 0.0164)	0.114	-0.0049 (-0.0272, 0.0173)	0.663	-0.0107 (-0.0346, 0.0132)	0.380	0.0158 (0.0072, 0.0243)	0.000	0.0094 (-0.0004, 0.0191)	0.059
vWF	0.0206 (0.0016, 0.0396)	0.033	0.0160 (-0.0045, 0.0365)	0.125	0.0098 (-0.0047, 0.0243)	0.184	0.0058 (-0.0102, 0.0219)	0.477	-0.0257 (-0.0641, 0.0126)	0.189	-0.0223 (-0.0632, 0.0185)	0.284	0.0163 (0.0007, 0.0318)	0.041	0.0080 (-0.0096, 0.0256)	0.372

RARRES2	-0.0019 (-0.0077, 0.0039)	0.521	-0.0027 (-0.0090, 0.0035)	0.391	-0.0122 (-0.0174, -0.0070)	0.000	-0.0070 (-0.0125, -0.0014)	0.013	-0.0100 (-0.0196, -0.0004)	0.041	-0.0070 (-0.0173, 0.0033)	0.185	-0.0130 (-0.0192, -0.0068)	0.000	-0.0076 (-0.0142, -0.0010)	0.024
REG4	-0.0011 (-0.0173, 0.0151)	0.894	0.0021 (-0.0166, 0.0208)	0.825	-0.0106 (-0.0243, 0.0030)	0.127	-0.0038 (-0.0185, 0.0109)	0.611	-0.0083 (-0.0344, 0.0177)	0.530	-0.0045 (-0.0329, 0.0238)	0.753	-0.0112 (-0.0273, 0.0048)	0.171	-0.0036 (-0.0209, 0.0136)	0.682
REN	0.0014 (-0.0195, 0.0222)	0.899	0.0152 (-0.0087, 0.0390)	0.213	-0.0307 (-0.0491, -0.0123)	0.001	0.0030 (-0.0161, 0.0221)	0.760	-0.0322 (-0.0666, 0.0022)	0.067	0.0044 (-0.0315, 0.0403)	0.811	-0.0301 (-0.0519, -0.0082)	0.007	0.0034 (-0.0193, 0.0261)	0.769
RETN	0.0016 (-0.0119, 0.0151)	0.816	-0.0180 (-0.0337, -0.0023)	0.024	-0.0222 (-0.0327, -0.0117)	0.000	-0.0300 (-0.0414, -0.0186)	0.000	-0.0268 (-0.0465, -0.0070)	0.008	-0.0376 (-0.0592, -0.0160)	0.001	-0.0204 (-0.0328, -0.0080)	0.001	-0.0273 (-0.0409, -0.0138)	0.000
RNASE3	-0.0021 (-0.0340, 0.0298)	0.897	-0.0106 (-0.0483, 0.0271)	0.582	-0.0136 (-0.0354, 0.0082)	0.222	-0.0265 (-0.0506, -0.0023)	0.032	-0.0317 (-0.0735, 0.0101)	0.137	-0.0339 (-0.0807, 0.0129)	0.156	-0.0057 (-0.0312, 0.0199)	0.665	-0.0230 (-0.0513, 0.0053)	0.111
ROR1	-0.0019 (-0.0116, 0.0078)	0.703	-0.0171 (-0.0285, -0.0058)	0.003	-0.0032 (-0.0113, 0.0049)	0.439	-0.0095 (-0.0184, -0.0006)	0.036	-0.0180 (-0.0334, -0.0027)	0.022	-0.0275 (-0.0446, -0.0104)	0.002	0.0025 (-0.0070, 0.0121)	0.602	-0.0030 (-0.0135, 0.0074)	0.567
RTN4R	0.0049 (-0.0040, 0.0138)	0.276	-0.0027 (-0.0124, 0.0070)	0.586	-0.0090 (-0.0175, -0.0005)	0.038	-0.0034 (-0.0120, 0.0053)	0.445	-0.0154 (-0.0322, 0.0015)	0.074	-0.0127 (-0.0301, 0.0047)	0.152	-0.0065 (-0.0163, 0.0033)	0.191	-0.0003 (-0.0101, 0.0096)	0.960
S100P	-0.0031 (-0.0106, 0.0045)	0.424	-0.0058 (-0.0147, 0.0030)	0.196	-0.0058 (-0.0153, 0.0036)	0.223	-0.0074 (-0.0179, 0.0030)	0.163	-0.0113 (-0.0306, 0.0080)	0.250	-0.0166 (-0.0382, 0.0051)	0.134	-0.0038 (-0.0143, 0.0068)	0.485	-0.0044 (-0.0161, 0.0073)	0.463
SCF	-0.0005 (-0.0098, 0.0087)	0.909	-0.0106 (-0.0213, 0.0001)	0.052	-0.0062 (-0.0141, 0.0018)	0.127	-0.0087 (-0.0174, 0.0000)	0.050	-0.0074 (-0.0225, 0.0076)	0.333	-0.0153 (-0.0319, 0.0013)	0.071	-0.0058 (-0.0151, 0.0035)	0.222	-0.0064 (-0.0166, 0.0039)	0.222
SCGB3A2	-0.0318 (-0.0546, -0.0089)	0.006	-0.0106 (-0.0364, 0.0152)	0.421	-0.0089 (-0.0247, 0.0069)	0.270	-0.0092 (-0.0263, 0.0079)	0.290	-0.0090 (-0.0421, 0.0240)	0.593	-0.0087 (-0.0450, 0.0276)	0.639	-0.0089 (-0.0263, 0.0086)	0.320	-0.0090 (-0.0278, 0.0099)	0.350
SDC4	-0.0076 (-0.0224, 0.0071)	0.312	0.0008 (-0.0167, 0.0184)	0.925	0.0023 (-0.0107, 0.0152)	0.729	-0.0006 (-0.0150, 0.0138)	0.936	0.0101 (-0.0154, 0.0356)	0.439	0.0093 (-0.0194, 0.0381)	0.524	-0.0005 (-0.0154, 0.0145)	0.953	-0.0034 (-0.0200, 0.0131)	0.683
SELE	0.0061 (-0.0103, 0.0225)	0.467	-0.0030 (-0.0218, 0.0159)	0.757	-0.0142 (-0.0273, -0.0011)	0.034	-0.0110 (-0.0250, 0.0031)	0.127	-0.0206 (-0.0453, 0.0041)	0.103	-0.0260 (-0.0529, 0.0010)	0.059	-0.0119 (-0.0274, 0.0035)	0.130	-0.0074 (-0.0239, 0.0092)	0.384
SELP	-0.0007 (-0.0134, 0.0120)	0.915	-0.0069 (-0.0218, 0.0081)	0.367	-0.0059 (-0.0162, 0.0044)	0.261	-0.0055 (-0.0169, 0.0059)	0.343	-0.0027 (-0.0228, 0.0174)	0.791	-0.0074 (-0.0299, 0.0152)	0.521	-0.0072 (-0.0191, 0.0048)	0.239	-0.0062 (-0.0193, 0.0070)	0.358
SEMA3E	-0.0005 (-0.0075, 0.0066)	0.893	-0.0084 (-0.0164, -0.0003)	0.043	-0.0081 (-0.0148, -0.0015)	0.016	-0.0089 (-0.0160, -0.0017)	0.015	-0.0178 (-0.0305, -0.0050)	0.006	-0.0170 (-0.0307, -0.0033)	0.015	-0.0044 (-0.0121, 0.0034)	0.270	-0.0056 (-0.0140, 0.0028)	0.190
SERPINA12	0.0169 (-0.0125, 0.0464)	0.260	0.0254 (-0.0095, 0.0603)	0.153	0.0091 (-0.0114, 0.0295)	0.384	0.0132 (-0.0094, 0.0357)	0.252	0.0179 (-0.0214, 0.0571)	0.372	0.0093 (-0.0343, 0.0530)	0.676	0.0054 (-0.0185, 0.0293)	0.659	0.0137 (-0.0126, 0.0400)	0.309
SERPINB6	-0.0010 (-0.0094, 0.0074)	0.823	-0.0007 (-0.0106, 0.0092)	0.889	-0.0101 (-0.0176, -0.0026)	0.009	-0.0139 (-0.0222, -0.0056)	0.001	-0.0146 (-0.0293, 0.0001)	0.052	-0.0203 (-0.0367, -0.0039)	0.015	-0.0080 (-0.0167, 0.0007)	0.071	-0.0110 (-0.0206, -0.0015)	0.024
SERPINB8	-0.0013 (-0.0158, 0.0132)	0.862	-0.0086 (-0.0258, 0.0085)	0.325	-0.0182 (-0.0302, -0.0061)	0.003	-0.0227 (-0.0360, -0.0094)	0.001	-0.0188 (-0.0411, 0.0034)	0.097	-0.0166 (-0.0415, 0.0083)	0.191	-0.0177 (-0.0321, -0.0033)	0.016	-0.0246 (-0.0405, -0.0086)	0.002
SHPS-1	0.0107 (0.0000, 0.0215)	0.051	0.0058 (-0.0069, 0.0185)	0.371	-0.0063 (-0.0155, 0.0029)	0.181	-0.0098 (-0.0200, 0.0004)	0.060	-0.0163 (-0.0341, 0.0015)	0.073	-0.0202 (-0.0402, -0.0003)	0.047	-0.0023 (-0.0131, 0.0084)	0.673	-0.0064 (-0.0183, 0.0054)	0.287
SIGLEC7	0.0028 (-0.0051, 0.0108)	0.486	0.0032 (-0.0059, 0.0123)	0.488	-0.0072 (-0.0143, 0.0000)	0.049	-0.0026 (-0.0103, 0.0052)	0.516	-0.0253 (-0.0391, -0.0116)	0.000	-0.0225 (-0.0375, -0.0074)	0.003	-0.0001 (-0.0084, 0.0082)	0.977	0.0049 (-0.0041, 0.0139)	0.287
SLAMF7	0.0205 (0.0030, 0.0381)	0.022	0.0185 (-0.0024, 0.0393)	0.082	-0.0050 (-0.0206, 0.0106)	0.531	-0.0024 (-0.0197, 0.0148)	0.783	-0.0116 (-0.0408, 0.0176)	0.436	-0.0023 (-0.0349, 0.0302)	0.888	-0.0024 (-0.0210, 0.0162)	0.801	-0.0038 (-0.0243, 0.0168)	0.719
SNAP23	-0.0089 (-0.0196, 0.0017)	0.101	-0.0013 (-0.0140, 0.0113)	0.839	-0.0108 (-0.0195, -0.0021)	0.015	-0.0125 (-0.0221, -0.0029)	0.010	-0.0137 (-0.0308, 0.0034)	0.116	-0.0097 (-0.0288, 0.0095)	0.324	-0.0091 (-0.0191, 0.0008)	0.072	-0.0121 (-0.0232, -0.0011)	0.032
SOD2	-0.0005 (-0.0058, 0.0047)	0.843	0.0013 (-0.0050, 0.0075)	0.688	-0.0003 (-0.0052, 0.0046)	0.902	0.0012 (-0.0041, 0.0066)	0.651	0.0006 (-0.0103, 0.0114)	0.920	-0.0011 (-0.0133, 0.0110)	0.857	-0.0007 (-0.0058, 0.0043)	0.781	0.0016 (-0.0040, 0.0072)	0.582
SORT1	-0.0045 (-0.0107, 0.0017)	0.152	-0.0018 (-0.0091, 0.0054)	0.621	-0.0044 (-0.0110, 0.0022)	0.192	-0.0015 (-0.0088, 0.0058)	0.687	-0.0026 (-0.0161, 0.0109)	0.704	-0.0021 (-0.0171, 0.0130)	0.787	-0.0051 (-0.0125, 0.0023)	0.179	-0.0018 (-0.0099, 0.0064)	0.672
SOST	0.0025 (-0.0084, 0.0134)	0.658	0.0024 (-0.0105, 0.0153)	0.717	-0.0033 (-0.0130, 0.0064)	0.503	-0.0047 (-0.0153, 0.0058)	0.380	-0.0011 (-0.0192, 0.0171)	0.909	-0.0009 (-0.0210, 0.0192)	0.932	-0.0037 (-0.0151, 0.0077)	0.522	-0.0061 (-0.0186, 0.0064)	0.341
SPON1	0.0033 (-0.0021, 0.0087)	0.226	0.0030 (-0.0033, 0.0093)	0.354	-0.0097 (-0.0162, -0.0031)	0.004	-0.0082 (-0.0154, -0.0010)	0.025	-0.0176 (-0.0298, -0.0054)	0.005	-0.0156 (-0.0292, -0.0021)	0.024	-0.0064 (-0.0142, 0.0015)	0.111	-0.0058 (-0.0143, 0.0027)	0.181
SPON2	0.0004 (-0.0037, 0.0045)	0.845	-0.0038 (-0.0086, 0.0009)	0.116	-0.0059 (-0.0112, -0.0006)	0.029	-0.0063 (-0.0121, -0.0005)	0.034	-0.0087 (-0.0202, 0.0029)	0.142	-0.0118 (-0.0246, 0.0011)	0.072	-0.0049 (-0.0106, 0.0008)	0.090	-0.0044 (-0.0106, 0.0018)	0.165
SRC	-0.0113 (-0.0208, -0.0017)	0.021	-0.0028 (-0.0142, 0.0085)	0.623	-0.0068 (-0.0178, 0.0042)	0.226	-0.0055 (-0.0176, 0.0066)	0.369	-0.0019 (-0.0240, 0.0202)	0.867	0.0007 (-0.0239, 0.0252)	0.958	-0.0088 (-0.0213, 0.0037)	0.167	-0.0072 (-0.0210, 0.0066)	0.304
SSC4D	0.0023 (-0.0310, 0.0357)	0.891	-0.0059 (-0.0415, 0.0297)	0.744	-0.0234 (-0.0525, 0.0057)	0.115	-0.0235 (-0.0533, 0.0064)	0.123	-0.0445 (-0.0948, 0.0057)	0.082	-0.0304 (-0.0813, 0.0205)	0.242	-0.0158 (-0.0516, 0.0200)	0.388	-0.0217 (-0.0587, 0.0152)	0.249
ST2	0.0000 (-0.0114, 0.0114)	0.998	0.0004 (-0.0130, 0.0138)	0.953	-0.0094 (-0.0201, 0.0013)	0.084	0.0011 (-0.0107, 0.0128)	0.859	-0.0138 (-0.0333, 0.0057)	0.165	-0.0001 (-0.0216, 0.0215)	0.995	-0.0078 (-0.0206, 0.0050)	0.232	0.0006 (-0.0135, 0.0147)	0.938
STK4	-0.0079 (-0.0215, 0.0058)	0.259	-0.0056 (-0.0218, 0.0106)	0.498	-0.0122 (-0.0259, 0.0014)	0.078	-0.0156 (-0.0307, -0.0005)	0.042	-0.0016 (-0.0274, 0.0242)	0.902	-0.0054 (-0.0342, 0.0234)	0.713	-0.0160 (-0.0321, 0.0000)	0.050	-0.0199 (-0.0377, -0.0021)	0.029
SUMF2	-0.0096 (-0.0274, 0.0083)	0.293	-0.0250 (-0.0457, -0.0044)	0.018	-0.0261 (-0.0399, -0.0123)	0.000	-0.0313 (-0.0462, -0.0164)	0.000	-0.0395 (-0.0666, -0.0125)	0.004	-0.0272 (-0.0563, 0.0020)	0.068	-0.0210 (-0.0370, -0.0049)	0.010	-0.0323 (-0.0497, -0.0149)	0.000
TF	0.0059 (-0.0022, 0.0141)	0.154	0.0030 (-0.0067, 0.0127)	0.544	-0.0032 (-0.0104, 0.0041)	0.397	-0.0061 (-0.0142, 0.0019)	0.136	-0.0028 (-0.0174, 0.0117)	0.702	-0.0083 (-0.0245, 0.0079)	0.315	-0.0034 (-0.0117, 0.0049)	0.424	-0.0053 (-0.0145, 0.0039)	0.255
TFF2	0.0031 (-0.0136, 0.0198)	0.716	0.0108 (-0.0083, 0.0299)	0.268	-0.0067 (-0.0204, 0.0070)	0.341	0.0071 (-0.0075, 0.0218)	0.340	-0.0033 (-0.0293, 0.0228)	0.807	0.0045 (-0.0239, 0.0329)	0.755	-0.0078 (-0.0239, 0.0084)	0.345	0.0073 (-0.0099, 0.0245)	0.403
TFF3	0.0107 (-0.0023, 0.0237)	0.107	0.0121 (-0.0031, 0.0272)	0.119	-0.0136 (-0.0235, -0.0037)	0.007	-0.0138 (-0.0243, -0.0032)	0.010	-0.0099 (-0.0283, 0.0086)	0.294	-0.0133 (-0.0332, 0.0067)	0.193	-0.0150 (-0.0267, -0.0033)	0.012	-0.0140 (-0.0264, -0.0015)	0.028
TFPI	0.0024 (-0.0069, 0.0117)	0.609	-0.0043 (-0.0153, 0.0066)	0.437	-0.0108 (-0.0188, -0.0028)	0.008	-0.0103 (-0.0190, -0.0015)	0.021	-0.0096 (-0.0246, 0.0055)	0.213	-0.0043 (-0.0212, 0.0126)	0.616	-0.0110 (-0.0204, -0.0016)	0.021	-0.0129 (-0.0231, -0.0028)	0.013
TGM2	-0.0028 (-0.0167, 0.0111)	0.691	-0.0090 (-0.0255, 0.0075)	0.285	-0.0054 (-0.0162, 0.0053)	0.323	-0.0026 (-0.0144, 0.0091)	0.661	-0.0153 (-0.0386, 0.0080)	0.198	-0.0090 (-0.0348, 0.0169)	0.496	-0.0016 (-0.0130, 0.0099)	0.785	-0.0007 (-0.0132, 0.0118)	0.911
THBS2	-0.0018 (-0.0065, 0.0028)	0.436	-0.0046 (-0.0101, 0.0009)	0.101	-0.0029 (-0.0090, 0.0032)	0.348	-0.0017 (-0.0084, 0.0050)	0.612	-0.0013 (-0.0139, 0.0113)	0.837	-0.0020 (-0.0160, 0.0121)	0.783	-0.0036 (-0.0104, 0.0031)	0.292	-0.0024 (-0.0099, 0.0050)	0.517
THOP1	0.0025 (-0.0056, 0.0106)	0.540	0.0011 (-0.0084, 0.0105)	0.822	-0.0087 (-0.0161, -0.0012)	0.023	-0.0089 (-0.0171, -0.0007)	0.033	-0.0131 (-0.0269, 0.0007)	0.062	-0.0087 (-0.0240, 0.0065)	0.263	-0.0066 (-0.0155, 0.0023)	0.144	-0.0091 (-0.0188, 0.0007)	0.069
THPO	-0.0021 (-0.0097, 0.0054)	0.578	0.0001 (-0.0088, 0.0090)	0.978	-0.0003 (-0.0088, 0.0082)	0.944	-0.0006 (-0.0099, 0.0088)	0.900	-0.0042 (-0.0205, 0.0122)	0.618	-0.0041 (-0.0223, 0.0141)	0.659	0.0013 (-0.0086, 0.0112)	0.799	0.0004 (-0.0105, 0.0113)	0.939
TIE2	0.0031 (-0.0030, 0.0091)	0.324	0.0007 (-0.0065, 0.0079)	0.857	-0.0007 (-0.0072, 0.0058)	0.838	0.0009 (-0.0063, 0.0080)	0.809	-0.0060 (-0.0189, 0.0068)	0.358	-0.0068 (-0.0211, 0.0076)	0.354	0.0014 (-0.0060, 0.0088)	0.713	0.0036 (-0.0046, 0.0117)	0.394
TIMP4	-0.0048 (-0.0175, 0.0079)	0.460	-0.0035 (-0.0184, 0.0114)	0.648	-0.0180 (-0.0280, -0.0080)	0.000	-0.0115 (-0.0224, -0.0007)	0.037	-0.0266 (-0.0460, -0.0072)	0.007	-0.0170 (-0.0384, 0.0043)	0.118	-0.0144 (-0.0260, -0.0028)	0.015	-0.0088 (-0.0214, 0.0037)	0.169
TINAGL1	0.0021 (-0.0042, 0.0083)	0.517	-0.0054 (-0.0127, 0.0019)	0.147	0.0003 (-0.0054, 0.0060)	0.916	-0.0015 (-0.0078, 0.0048)	0.649	-0.0088 (-0.0198, 0.0021)	0.114	-0.0088 (-0.0211, 0.0034)	0.156	0.0040 (-0.0027, 0.0107)	0.242	0.0018 (-0.0056, 0.0092)	0.633
TLT-2	0.0010 (-0.0087, 0.0106)	0.846	-0.0128 (-0.0240, -0.0015)	0.026	-0.0061 (-0.0166, 0.0045)	0.260	-0.0103 (-0.0219, 0.0013)	0.081	-0.0142 (-0.0337, 0.0052)	0.152	-0.0292 (-0.0506, -0.0077)	0.008	-0.0027 (-0.0153, 0.0099)	0.676	-0.0034 (-0.0173, 0.0105)	0.631
TM	0.0050 (-0.0031, 0.0131)	0.229	-0.0045 (-0.0141, 0.0050)	0.351	-0.0016 (-0.0096, 0.0064)	0.692	-0.0057 (-0.0145, 0.0031)	0.202	-0.0100 (-0.0253, 0.0052)	0.197	-0.0170 (-0.0338, -0.0002)	0.048	0.0015 (-0.0079, 0.0109)	0.759	-0.0013 (-0.0116, 0.0091)	0.810
TNF-R1	0.0085 (-0.0017, 0.0187)	0.104	-0.0033 (-0.0149, 0.0082)	0.572	-0.0200 (-0.0289, -0.0111)	0.000	-0.0203 (-0.0296, -0.0109)	0.000	-0.0328 (-0.0499, -0.0158)	0.000	-0.0340 (-0.0520, -0.0160)	0.000	-0.0150 (-0.0254, -0.0046)	0.005	-0.0147 (-0.0257, -0.0037)	0.009
TNF-R2	0.0019 (-0.0093, 0.0131)	0.738	-0.0128 (-0.0256, 0.0001)	0.051	-0.0077 (-0.0183, 0.0029)	0.155	-0.0091 (-0.0205, 0.0024)	0.120	0.0010 (-0.0189, 0.0209)	0.923	0.0004 (-0.0213, 0.0221)	0.969	-0.0111 (-0.0236, 0.0015)	0.083	-0.0123 (-0.0259, 0.0012)	0.075
TNFRSF10A	0.0052 (-0.0042, 0.0147)	0.277	0.0081 (-0.0027, 0.0189)	0.143	-0.0129 (-0.0217, -0.0040)	0.004	-0.0054 (-0.0147, 0.0040)	0.261	-0.0208 (-0.0376, -0.0041)	0.015	-0.0150 (-0.0328, 0.0028)	0.098	-0.0099 (-0.0203, 0.0005)	0.062	-0.0022 (-0.0132, 0.0089)	0.699
TNFRSF10C	-0.0022 (-0.0171, 0.0127)	0.772	-0.0102 (-0.0277, 0.0073)	0.255	-0.0213 (-0.0329, -0.0098)	0.000	-0.0257 (-0.0384, -0.0129)	0.000	-0.0261 (-0.0479, -0.0043)	0.019	-0.0359 (-0.0601, -0.0117)	0.004	-0.0193 (-0.0329, -0.0057)	0.005	-0.0216 (-0.0366, -0.0066)	0.005
TNFRSF11A	0.0080 (-0.0032, 0.0191)	0.162	0.0009 (-0.0115, 0.0134)	0.883	-0.0170 (-0.0275, -0.0065)	0.002	-0.0144 (-0.0252, -0.0036)	0.009	-0.0255 (-0.0461, -0.0049)	0.015	-0.0252 (-0.0466, -0.0037)	0.021	-0.0139 (-0.0260, -0.0019)	0.023	-0.0099 (-0.0223, 0.0026)	0.119
TNFRSF13B	0.0028 (-0.0075, 0.0131)	0.597	-0.0027 (-0.0148, 0.0094)	0.662	-0.0076 (-0.0184, 0.0032)	0.167	-0.0063 (-0.0181, 0.0055)	0.294	-0.0108 (-0.0299, 0.0083)	0.268	-0.0170 (-0.0381, 0.0041)	0.114	-0.0065 (-0.0196, 0.0066)	0.334	-0.0024 (-0.0168, 0.0120)	0.743
TNFRSF14	0.0072 (-0.0022, 0.0166)	0.132	-0.0061 (-0.0169, 0.0047)	0.268	-0.0132 (-0.0217, -0.0046)	0.003	-0.0132 (-0.0224, -0.0040)	0.005	-0.0224 (-0.0386, -0.0063)	0.007	-0.0258 (-0.0431, -0.0084)	0.004	-0.0095 (-0.0196, 0.0006)	0.065	-0.0083 (-0.0191, 0.0025)	0.134
TNFSF13B	-0.0020 (-0.0116, 0.0077)	0.691	-0.0056 (-0.0169, 0.0057)	0.331	-0.0104 (-0.0192, -0.0016)	0.021	-0.0072 (-0.0169, 0.0025)	0.144	-0.0147 (-0.0314, 0.0020)	0.085	-0.0113 (-0.0299, 0.0073)	0.232	-0.0084 (-0.0188, 0.0020)	0.112	-0.0053 (-0.0167, 0.0060)	0.359
TR	0.0059 (-0.0072, 0.0191)	0.376	-0.0073 (-0.0226, 0.0080)	0.351	-0.0056 (-0.0176, 0.0063)	0.356	-0.0049 (-0.0180, 0.0081)	0.461	-0.0075 (-0.0302, 0.0151)	0.514	-0.0139 (-0.0390, 0.0112)	0.278	-0.0047 (-0.0187, 0.0093)	0.511	-0.0026 (-0.0180, 0.0127)	0.737
TR-AP	0.0022 (-0.0076, 0.0120)	0.659	0.0010 (-0.0105, 0.0125)	0.862	-0.0106 (-0.0190, -0.0023)	0.012	-0.0053 (-0.0144, 0.0039)	0.260	-0.0215 (-0.0367, -0.0063)	0.006	-0.0160 (-0.0330, 0.0011)	0.066	-0.0063 (-0.0163, 0.0036)	0.212	-0.0016 (-0.0125, 0.0093)	0.775
TRAIL-R2	0.0020 (-0.0083, 0.0124)	0.701	-0.0034 (-0.0152, 0.0084)	0.574	-0.0068 (-0.0183, 0.0047)	0.245	-0.0046 (-0.0168, 0.0077)	0.466	-0.0008 (-0.0230, 0.0213)	0.941	-0.0017 (-0.0256, 0.0221)	0.886	-0.0091 (-0.0225, 0.0043)	0.182	-0.0060 (-0.0203, 0.0084)	0.416
TSHB	-0.0118 (-0.0377, 0.0141)	0.372	-0.0045 (-0.0351, 0.0262)	0.776	-0.0009 (-0.0194, 0.0175)	0.920	0.0045 (-0.0160, 0.0249)	0.668	-0.0073 (-0.0472, 0.0326)	0.720	-0.0067 (-0.0514, 0.0380)	0.769	0.0016 (-0.0183, 0.0215)	0.874	0.0083 (-0.0138, 0.0303)	0.461
TXNDC5	0.0006 (-0.0096, 0.0108)	0.906	-0.0044 (-0.0165, 0.0076)	0.470	-0.0217 (-0.0317, -0.0116)	0.000	-0.0196 (-0.0305, -0.0088)	0.000	-0.0334 (-0.0513, -0.0156)	0.000	-0.0297 (-0.0492, -0.0102)	0.003	-0.0171 (-0.0293, -0.0049)	0.006	-0.0154 (-0.0286, -0.0022)	0.022
TYMP	0.0061 (-0.0125, 0.0247)	0.523	0.0085 (-0.0134, 0.0305)	0.445	-0.0202 (-0.0364, -0.0041)	0.014	-0.0161 (-0.0339, 0.0017)	0.076	-0.0331 (-0.0637, -0.0025)	0.034	-0.0314 (-0.0655, 0.0027)	0.071	-0.0152 (-0.0342, 0.0037)	0.116	-0.0097 (-0.0306, 0.0112)	0.364
TYRO3	-0.0043 (-0.0115, 0.0028)	0.235	-0.0066 (-0.0149, 0.0018)	0.124	0.0023 (-0.0047, 0.0093)	0.521	0.0057 (-0.0020, 0.0135)	0.144	-0.0042 (-0.0180, 0.0096)	0.550	-0.0050 (-0.0203, 0.0103)	0.520	0.0049 (-0.0032, 0.0130)	0.239	0.0090 (0.0000, 0.0179)	0.049
U-PAR	0.0069 (-0.0029, 0.0167)	0.168	0.0003 (-0.0107, 0.0114)	0.951	-0.0110 (-0.0194, -0.0026)	0.010	-0.0087 (-0.0176, 0.0002)	0.056	-0.0186 (-0.0341, -0.0032)	0.018	-0.0145 (-0.0312, 0.0021)	0.088	-0.0079 (-0.0178, 0.0021)	0.121	-0.0064 (-0.0170, 0.0041)	0.233
USP8	-0.0019 (-0.0098, 0.0060)	0.631	-0.0027 (-0.0120, 0.0066)	0.573	-0.0048 (-0.0123, 0.0026)	0.203	-0.0034 (-0.0117, 0.0048)	0.414	-0.0103 (-0.0253, 0.0046)	0.175	-0.0035 (-0.0203, 0.0134)	0.685	-0.0025 (-0.0110, 0.0060)	0.559	-0.0031 (-0.0125, 0.0063)	0.514
VCAN	0.0045 (-0.0044, 0.0135)	0.322	0.0035 (-0.0070, 0.0140)	0.517	0.0016 (-0.0067, 0.0100)	0.700	0.0013 (-0.0080, 0.0105)	0.784	-0.0055 (-0.0214, 0.0105)	0.502	0.0007 (-0.0170, 0.0184)	0.940	0.0046 (-0.0053, 0.0145)	0.363	0.0018 (-0.0091, 0.0126)	0.751
VEGFD	-0.0069 (-0.0174, 0.0036)	0.195	-0.0042 (-0.0162, 0.0078)	0.494	0.0045 (-0.0058, 0.0147)	0.392	0.0047 (-0.0066, 0.0159)	0.417	0.0003 (-0.0180, 0.0186)	0.977	-0.0045 (-0.0246, 0.0157)	0.663	0.0063 (-0.0062, 0.0187)	0.325	0.0071 (-0.0066, 0.0208)	0.311
VSIG2	0.0028 (-0.0110, 0.0167)	0.689	0.0071 (-0.0091, 0.0233)	0.393	-0.0119 (-0.0241, 0.0004)	0.057	-0.0051 (-0.0181, 0.0080)	0.448	-0.0177 (-0.0415, 0.0061)	0.145	-0.0163 (-0.0422, 0.0096)	0.218	-0.0098 (-0.0240, 0.0043)	0.173	-0.0018 (-0.0169, 0.0133)	0.814
XCL1	-0.0102 (-0.0255, 0.0051)	0.191	-0.0137 (-0.0317, 0.0043)	0.136	-0.0123 (-0.0251, 0.0005)	0.059	-0.0103 (-0.0243, 0.0037)	0.148	-0.0206 (-0.0452, 0.0040)	0.100	-0.0117 (-0.0387, 0.0154)	0.399	-0.0090 (-0.0239, 0.0059)	0.237	-0.0095 (-0.0259, 0.0068)	0.254
hOSCAR	0.0012 (-0.0048, 0.0072)	0.685	-0.0034 (-0.0105, 0.0036)	0.341	-0.0035 (-0.0100, 0.0029)	0.281	-0.0046 (-0.0116, 0.0025)	0.205	-0.0046 (-0.0177, 0.0085)	0.495	-0.0070 (-0.0216, 0.0075)	0.342	-0.0033 (-0.0105, 0.0039)	0.369	-0.0039 (-0.0118, 0.0041)	0.341
t-PA	-0.0018 (-0.0190, 0.0155)	0.841	0.0018 (-0.0172, 0.0207)	0.854	-0.0215 (-0.0335, -0.0094)	0.000	-0.0069 (-0.0191, 0.0054)	0.271	-0.0210 (-0.0437, 0.0016)	0.068	0.0008 (-0.0224, 0.0241)	0.944	-0.0215 (-0.0357, -0.0073)	0.003	-0.0102 (-0.0247, 0.0043)	0.168
uPA	0.0046 (-0.0047, 0.0139)	0.329	-0.0017 (-0.0126, 0.0093)	0.766	0.0007 (-0.0070, 0.0084)	0.857	-0.0025 (-0.0110, 0.0061)	0.569	-0.0036 (-0.0187, 0.0114)	0.636	-0.0104 (-0.0274, 0.0066)	0.229	0.0026 (-0.0063, 0.0115)	0.562	-0.0008 (-0.0106, 0.0090)	0.872
vWF	0.0024 (-0.0167, 0.0214)	0.808	0.0032 (-0.0191, 0.0255)	0.777	-0.0176 (-0.0313, -0.0038)	0.012	-0.0079 (-0.0230, 0.0073)	0.310	-0.0328 (-0.0588, -0.0068)	0.013	-0.0096 (-0.0386, 0.0194)	0.515	-0.0110 (-0.0272, 0.0051)	0.181	-0.0066 (-0.0244, 0.0112)	0.469

Milk and fermented milk intake and Ischemic Heart Disease (IHD)

Objective

The aim of the study is to investigate associations between time updated information of milk and fermented milk consumption and risk of ischemic heart disease (IHD) as primary outcome and acute myocardial infarction (MI) as secondary outcome.

Ischemic heart disease (IHD) is a consequence of both genetic and environmental influences[(1)] and is the leading cause of years lost in Europe and globally.[(2,3)] While survival after a diagnosis of IHD has improved dramatically and is suggested to be largely explained by improved treatments,[(4,5)] the 40% reduction in age-standardized IHD incidence during the last two decades in Sweden, which is more accentuated in women,[(6)] can both be attributable to medical prevention efforts and population changes in lifestyle.[(7-9)] Individual lifestyle factors are by growing age becoming of stronger importance for the development of IHD[(1)] and one such factor is our diet. Specifically, there is uncertainty about the relevance of intakes of dairy products since previous study results have been conflicting,[(10-14)] without consensus in recommendations has been reached.[(14,15)] These discrepancies in results may be a consequence of different types of dairy products investigated, fat content and exposure width of examined intake. Both non-fermented and fermented milk are widely consumed and they may have differential effect on cardiovascular health.[(10-14,16,17)] Consumption patterns of different dairy products have however considerably changed during the last half century, with on average continued lowered intake of non-fermented milk in many settings[(18,19)] and these changes need to be accurately captured by the study design and analysis.[(20)]

We therefore used data from two large Swedish longitudinal cohorts consisting of women and men to assess the risk of non-fatal and fatal myocardial infarction and ischemic heart disease with a wide range of consumption patterns of non-fermented and fermented milk consumption. The aim of the study is to investigate associations between time-updated information of non-fermented and fermented milk consumption and risk of IHD and acute myocardial infarction. With use of a replication and discovery design in subcohorts of the larger cohorts, a secondary aim is to examine non-fermented and fermented milk consumption in relation to patterns of cardiovascular plasma proteomic concentrations.

Study Population

Swedish Mammography Cohort (SMC) and the Cohort of Swedish Men (COSM).
Examinations; 1987-90 (women only), 1997, 2008/2009.

Exclusions:

- Exclude due to missing baseline questionnaire. When 2009 is set to baseline, we base this on the combination of the 2008 & 2009 questionnaires, requiring participation in both.
- Exclude participants with an incorrect or a missing personal identity numbers and those with a history of cancer or death before baseline.
- Excluded those with an implausible energy intake in the baseline questionnaire (defined as 3 standard deviations from the log-transformed mean energy intake in women and men separately). This is already done for SMC 1987.
- Exclude those with an IHD diagnosis (as recorded in main and all other diagnoses) before baseline. We will exclude: ICD-8: 410-413, 426, 429; ICD-9: 410-414; ICD-10: I20-I25.

For baseline other than SMC-1987 and COSM-1997, exclusion variables based on the first three of the above criteria is called `exclude_baseline_1997` & `exclude_baseline_2009`. The last criteria is denoted by `prev_ihd1987`, `prev_ihd1997` & `prev_ihd2009`. The special treatment of this last criterion is due to a sensitivity analysis in which we ignore this exclusion criterion.

Baseline & time updates:

1987-1989: Mamdate (SMC)
1998: January 1st 1998
2008/2009: April 14th 2009

Don't update information on exposure and covariates for those with:

- Missing questionnaire
- Implausible energy intake

This applies only to the questionnaire of interest. For instance, in SMC, if 1997 questionnaire has implausible energy intake, but not 2009, then update will be done in 2009 for the analyses with baseline 1987.
Two variables are created for this purpose: `update_1997` and `update_2009` that have the value 1 if an individual's information should be updated for that questionnaire. These variables are based on the two conditions on missing questionnaire and implausible energy intake.

Main exposure

- Milk intake, including total non-fermented milk intake and milk by three fat content categories
- Fermented milk intake including yogurt and sour milk

Milk and fermented milk intake will be presented in grams.

1987:

- Milk
    - `egen milk1987 = rowtotal(gram_milk05_87 gram_milk15_87 gram_milk30_87)`
        - For one sensitivity analysis, create an indicator variable `milk_miss1987` if all three variables above are missing
    - Fat content specific
        - `gen milk_low1987 = gram_milk05_87`
        - `gen milk_medium1987 = gram_milk15_87`
        - `gen milk_high1987 = gram_milk30_87`
- Fermented milk
    - `egen fil1987 = rowtotal(gram_sourmilk05_87 gram_sourmilk30_87)`
        - For one sensitivity analysis, create an indicator variable `fil_miss1987` if both variables above are missing

1997:

- Milk
    - `egen milk1997 = rowtotal(gramf52 gramf54 gramf56)`
        - For one sensitivity analysis, create an indicator variable `milk_miss1997` if all three variables above are missing
    - Fat content specific
        - `gen milk_low1997 = gramf52`
        - `gen milk_medium1997 = gramf54`
        - `gen milk_high1997 = gramf56`

- Fermented milk
    - `egen fil1997 = rowtotal(gramf58 gramf60)`
        - For one sensitivity analysis, create an indicator variable `fil_miss1997` if both variables above are missing

2008/2009:

- Milk
    - `egen milk2009 = rowtotal(f15gr_1 f15gr_3 f15gr_5)`
        - For one sensitivity analysis, create an indicator variable `milk_miss2009` if all three variables above are missing
    - Fat content specific
        - `gen milk_low2009 = f15gr_1`
        - `gen milk_medium2009 = f15gr_3`
        - `gen milk_high2009 = f15gr_5`

- Fermented milk

- `egen fil2009 = rowtotal(f15gr_7 f15gr_9 f15gr_11 f15gr_13)`
    - For one sensitivity analysis, create an indicator variable `fil_miss2009` if all four variables above are missing
    - For some sensitivity analyses, get information without fruit yogurt:
        - `egen fil_nofruit2009 = rowtotal(f15gr_9 f15gr_11 f15gr_13)`

Outcome

Incident and previous IHD cases will be identified by linkage with the Swedish National Patient and Cause of Death Registers. To classify IHD diagnoses we will use the ICD-9 codes (International Classification of Diseases 10th Revision) 410-414 and the ICD-10 codes I20-I25. We will use first-ever primary (main diagnosis) inpatient IHD diagnosis or IHD (main) diagnosis indicated as cause of death after baseline.

Main outcome

- IHD:
    - ICD-9: 410-414
    - ICD-10: I20-I25

Subtype

- Acute myocardial infarction (MI)
    - ICD-9: 410
    - ICD-10: I21

End of follow-up; 31 December 2019

Censoring: The first of:

- Date of death
- End of follow-up
- Other subtype of IHD (only applicable in subtype-analyses)

Description of possible confounders

Sex	Stratification variable
Age	
Educational level	≤9 years, 10–12 years, >12 years, or other
Living alone	Yes/No
Leisure time exercise	<1 h/w, 1 h/w, 2-3 h/w, 4-5 h/w, >5 h/w
Walking/cycling	Never/Seldom, <20 min/d, 20-40 min/d, 40-60 min/d, 1-1.5 h/d, >1.5 h/d
Body mass index (BMI)	Weight in kg divided by height in m^2; continuous
Height	Height in cm, continuous
Total energy intake	kcal/day; continuous
Fermented milk / Non-fermented milk	Depending on the main exposure. Continuous
Cheese	Gram/day. Continuous
Alcohol consumption	Continuous
Fruits and vegetables	Servings/day of: Continuous
Red meat	Gram/day.<br>Continuous
Soft drinks and Juice	Servings/day. Continuous
Coffee	cups/day; continuous
Total fat intake	Energy-adjusted total fat intake. Continuous
Saturated fat intake	Energy-adjusted saturated fat intake. Continuous
Smoking status	Current, former, never
Vitamin- and mineral supplements	Yes/No
Weighted Charlson Comorbidity Index.	Charlson Comorbidity Index. From baseline, no time-update. Continuous
Major cardiovascular disease other than IHD (here called "CVD")	ICD8: 412, 420-429 ICD9: 415-438, ICD10: I26-I69. Yes/No. From baseline, no time-update
Diabetes mellitus	History of diabetes (yes/no), self-reported in combination with NPR. From baseline, no time-update

Models:

Model 1: adjust for sex through stratification + age.

Model 2: Sex, Age, Educational level, Living alone, Leisure time exercise, Walking/cycling, Body mass index (BMI), Height, Total energy intake, Fermented milk / Non-fermented milk, Cheese, Alcohol consumption, Fruits and vegetables, Red meat, Soft drinks and Juice, Coffee, Total fat intake, Saturated fat intake, Smoking status, Vitamin- and mineral supplements, baseline Weighted Charlson Comorbidity Index, baseline CVD, baseline Diabetes mellitus.

Statistical analyses

Time at risk of IHD for each participant will be calculated from baseline (according to the baseline dates described above) until the date of the first IHD diagnosis, date of death, end of follow-up or other subtype of IHD (only applicable in analyses concerning subtypes of IHD), whichever came first.

In the main analyses, if possible, we will use information of exposure and covariates from the following investigations; 1987-90 (women only), 1997, and 2008/2009. In the analysis we will time-update exposure and covariates. Missing information of covariates will be imputed, unless the complete questionnaire is missing or the questionnaire has implausible energy intake, in which case no update is made.

Cox proportional hazards regression models with time since baseline as time scale will be used to estimate hazard ratios with 95% confidence intervals (CI) for categories of milk and fermented milk intake. We will investigate total IHD and MI. When investigating MI, the other subtypes of IHD will be censored.

Intake will be divided into categories <200, 200-399, 400-599, and ≥600 grams per day for milk consumption and 0, 0-199, 200-399, ≥400 grams per day for fermented milk consumption. The category with the lowest intake will be used as reference (<200 grams for milk and 0 grams for fermented milk).

We will also look at the continuous version of Milk/fermented milk, in which we estimate as a linear variable, with HR presented per 200 gram. We will also treat the continuous variables as non-linear, using restricted cubic splines using three knots (percentiles 10, 50, 90). These results will be presented as graphs and tables containing HR for 200, 400, 600, 800 gram intake, compared to 100 gram intake as reference.

Analyses time to event

All analyses will include categorized main exposures, linear continuous main exposure and non-linear continuous main exposure (restricted cubic spline). There are two sets of adjustment variables, according to the two models above.

1) Descriptive statistics

2)

Start 1987-90 (mamdate) for SMC + time updated info from 1997 and 2008/2009.
Start 1998-01-01 for Pooled, SMC, and COSM + time updated info from 2008/2009.

3)

Start 1987-90 (mamdate) for SMC but no time update.
Start 1998-01-01 for Pooled, SMC, and COSM but no time update.
Start 2009-04-14 for Pooled, SMC, and COSM.

4) As in 3, but we remove those with missing information at baseline on all types of milk and all types of yogurt/sour milk respectively. The variables that defines whether or not milk is missing is called `milk_miss1987`, `milk_miss1997`, `milk_miss2009`. In the analyses where milk is the main exposure, we remove those with missing information on milk and correspondingly for the analyses where yogurt/sour milk is the main exposure.

5) As in Analysis 2 but only for fermented milk intake and in these analyses fermented milk with fruit is excluded. That is, before splitting data, `replace fil2009 = fil_nofruit2009`. Don't forget to re-categorize the categorical version of the variable.

6) As in 3 (2009) but only for fermented milk intake and in these analyses fermented milk with fruit is excluded. That is, `replace fil2009 = fil_nofruit2009`. Don't forget to re-categorize the categorical version of the variable.

7) Test the proportional hazard assumptions graphically using Schoenfeld residuals. As in 2). Use the final adjustment model 2. This is done on the first imputed data set, as if it was observed.

8) As in 3) but Complete case analyses. For SMC 1987-1990, do not adjust for the variables that are not in 1987-questionnaire, since they will be missing for everyone that died before 1997 or that didn't answer the 1997-questionnaire.

9) Substitution analyses according to Figure 2 in *doi: 10.1136/bmj.l6204*. Total dairy is defined as
milk + fermented milk + cheese.
Substitution hazard ratios to be calculated are when substituting fermented milk intake for non-fermented milk intake (meaning that we increase fermented milk and decrease non-fermented milk)
Baselines are 1987-1990, 1997 & 2009, with time updates.
The dairy product variables are all modelled linearly.

10) As in 2), but analyse each fat content of milk separately, as in analysis A in https://www.ncbi.nlm.nih.gov/pmc/articles/PMC7284719/. Adjust for other fat contents. Spline-curves for each fat-content separately. Due to the high number of zeros, the knots for the spline-variables needs to be decided manually, deviating from Harrell's recommendation.
For each fat content, delay entry or censor those with ≥200g milk with other fat content in the sense that no one is in the risk set when drinking ≥200g milk with other fat content. In Stata-terms it might look like this:
```
replace _st = 0 if (mellanmjölk + standardmjölk) >=200
```

11) As in 2), but stratified on the variables sex, year of birth, BMI, exercise, walking, marital status, education, Charlson comorbidity index, smoking status, and energy intake, using an interaction term. Wald test concerning potential effect modification is conducted. Milk/fermented milk treated as linear. Results presented as forest plot, with cut-offs as in Figure 2, https://doi.org/10.1371/journal.pmed.1003331

12) As in 2), but exclude individuals with pre-existing cardiovascular disease (`prev_cvd`), and diabetes (`prev_diabetes`).

13) As in 2), but include individuals with pre-existing IHD.

14) Austin, RR and RD. Högsta mot lägsta kategorin. To be continued.

15) SMC, start 1987, update 1997 (not 2009). Include individuals with pre-existing IHD. Outcome is time to general death. End of followup is 31 dec 2010, 30 sep 2015, & 31 dec 2019.
Three different adjustments:
Model 2: As above
Model 3: As model 2, but exclude coffee, soft drink, alcohol, fat intake, saturated fat intake, previous diabetes, previous CVD.
Model 4: As model 3, but use linear (instead of spline) adjustment of continuous covariates. Also, exclude walking/cycling.

16) As 02, IHD, model 2, for categorical and spline exposure. Calculate p-value for interaction terms with sex.

17) Splines (three knots) and Forest plots for the dose-response association between non-fermented and fermented milk intake with plasma protein biomarkers from Olink's panels CVD II, CVD III, and Metabolism. The subcohort SMCC in Uppsala is used for discovery (FDR cutoff 0.05), and the subcohort in Västerås (SMCC + COSMC) for replication. See below for details.

18) Difference between milk- and sour-milk estimates

19) Include a two-year washout period

20) Multivariable HRs for the association between milk-related proteins and IHD, continuous exposure including splines.

Variables from questionnaires

Variabelnamn	Definition 1987	Definition 1997	Definition 2008/2009	Kommentar
male				
smoke	Hämta information från 1997	tobac<br>1: Current<br>2: Former<br>3: Never	f27_1<br>1: Never<br>2: Current<br>3: Former	
bmi	weight_87 & height_87	bmi	q47/((q46/100)^2	Kräver förmodligen lite städning av data för alla tre enkäterna
height	height_87	height	q46	Enhet i cm
educat	edu_87<br>1-3: <=9 years<br>4: 10-12 years<br>5: >12 years<br>6: Missing	educat<br>1: <=9 years<br>2: 10-12 years<br>3: >12 years<br>4: Missing		Högsta värdet av 1987 och 1997 (2009 har inte den informationen)
walk	Hämta information från 1997	f37<br>1: Never/seldom<br>2: < 20 min/day<br>3: 20-40 min<br>4: 40-60 min<br>5: 1-1.5 hours<br>6: >1.5 hours/day	f1_1<br>1: Never/seldom<br>2: < 20 min/day<br>3: 20-40 min<br>4: 40-60 min<br>5: 1-1.5 hours<br>6: >1.5 hours/day	
exercise	Hämta information från 1997	f49<br>1: < 1 hour/week,<br>2: 1 hour,<br>3: 2-3 hours,<br>4: 4-5 hours,<br>5: >5 hours/week	f1_5<br>1-2: < 1 hour/week,<br>3: 1 hour,<br>4: 2-3 hours,<br>5: 4-5 hours,<br>6: >5 hours/week	
alcohol	Alco	col1	nut8	
coffee	freq_coffe	g68	rowtotal(f15fr_31 f15fr_33), miss	
alone	mar_status_87<br>2, 5: No<br>1, 3, 4: Yes	SMC: f336<br>1: No<br>2: Yes	if inlist(q221, 2, 3, 4) | (q222>1 & !missing(q222)): No	

		COSM: f1327<br>2: No<br>1, 3, 4: Yes	if ((q221==1) & (inlist(q222, 0, 1)) | (missing(q222) & !missing(q223))): Yes	
milk	rowtotal(gram_milk05_87 gram _milk15_87 gram milk30_87)	rowtotal(gramf52 gramf54 gramf56)	rowtotal(f15gr_1 f15gr_3 f15gr_5)	
fil	rowtotal(gram_sourmilk30_87 gram_sourmilk05_87)	rowtotal(gramf58 gramf60)	rowtotal(f15gr_7 f15gr_9 f15gr_11 f15gr_13)	
fil_nofruit	Only applicable to 2009	Only applicable to 2009	rowtotal(f15gr_11 f15gr_9 f15gr_13)	
cheese	gram_cheese_87	rowtotal(gramf72 gramf74)	rowtotal(f15gr_45 f15gr_47)	
vegs	rowtotal(freq_rootvegetable_87 freq_whitecabbage_87 freq_tomato_87 freq_lettuce_87 freq_spinach_87 freq_brownbeans_87), miss	rowtotal(g133 g134 g135 g136 g137 g138 g139 g140 g141 g142 g143 g144 g145 g146), miss	rowtotal(f17fr_45 f17fr_46 f17fr_47 f17fr_48 f17fr_49 f17fr_50 f17fr_51 f17fr_52 f17fr_53 f17fr_54 f17fr_55 f17fr_56 f17fr_57 f17fr_58 f17fr_59 f17fr_60 f17fr_61 f17fr_62 f17fr_63 f17fr_64), miss	
fruits	rowtotal(freq_apple_87 freq_citrusfruit_87 freq_banana_87), miss	rowtotal(g148 g150 g151 g152 g153), miss	rowtotal(f17fr_65 f17fr_67 f17fr_68 f17fr_69 f17fr_70), miss	
fv	rowtotal(fruits vegs), miss	rowtotal(fruits vegs), miss	rowtotal(fruits vegs), miss	

`red_meat`	rowtotal(gram_meat_87 gram_meatstew_87 gram_bacon_87 gram_mincedmeat_87 gram_sausage_87 gram_sandwichfill_87 gram_pate_87 gram_blackpudding_87 gram_liver_87), miss	rowtotal(gramf115 gramf116 gramf117 gramf118 gramf119 gramf120 gramf121 gramf122), miss	rowtotal(f15gr_51 f15gr_53 f17gr_15 f17gr_16 f17gr_17 f17gr_18 f17gr_19 f17gr_20 f17gr_21 f17gr_22 f17gr_23 f17gr_24 f17gr_25 f17gr_26), miss	
`softdrink`	rowtotal(freq_juice_87 freq_softdrink_87 freq_fruitdrink_87), miss	rowtotal(g62 g149), miss	rowtotal(f15fr_17 f15fr_19 f15fr_21 f15fr_23 f17fr_66), miss	
`supplements`	Hämta information från 1997	inlist(f194, 1, 2) if inlist(f194, 1, 2, 3)	inlist(f23_1, 2, 3) if inlist(f23_1, 1, 2, 3)	
`energy_total`	ener	col4	nut1	
`adj_fat`	adjfat87	col_eadj6	enut3	
`adj_sat_fat`	adjsfat87	col_eadj14	enut13	
`diabetes`	Finns inte information i 1987. Här får patientregistret bestämma	f260==1 | f261!=.	q41==2 | q42!=.	Denna variabel kombineras senare med `prev_diabetes` från patientregistret, och kommer då gå under namnet `prev_diabetes`

Variables from patient register. For exclusion and adjustment

	ICD-8	ICD-9	ICD-10	Kommentar
prev_diabetes	250	250	E10-E14	Både huvuddiagnos och bidiagnos. Slutenvård
cci_w (weighted)	Se Stata-kod			Både huvuddiagnos och bidiagnos. Slutenvård
cci_unw (unweighted)				
prev_cvd	412, 420-429	415-438	I26-I69	Både huvuddiagnos och bidiagnos. Slutenvård
prev_ihd	410-413, 426, 429	410-414	I20-I25	Både huvuddiagnos och bidiagnos. Slutenvård
prev_cancer	14 15 16 17 18 190 192 193 194 195 196 197 198 199 200 201 202 203 204 205	14 15 16 170 171 172 174 175 176 177 178 179 18 19 20	C0 C1 C2 C3 C40 C41 C42 C43 C45 C46 C47 C48 C49 C5 C6 C7 C8 C9	Både huvuddiagnos och bidiagnos. Slutenvård

Outcome variables from patient register. For each outcome, we create one variable per baseline, giving the first date of the outcome since baseline.

	ICD-9	ICD-10	Kommentar
ihd_outcome	410-414	I20-I25	Enbart huvuddiagnos. Slutenvård.
mi_outcome	410	I21	Enbart huvuddiagnos. Slutenvård.

Analysis non-fermented and fermented milk in relation to cardiometabolic proteins

Data
Två data (SMCC & COSMC) som är färdiga från Liisa.

Variabler
Utfall:
Proteinerna i OLINK-panelerna CVD2, CVD3 & Metabolism

Huvudexponeringar:
Mjölk och filmjölk

Justeringar:
A) `male, visitage, visityear`
B) UTÖVER A), lägg även till `educat, livealone, exercise, walking, bmi, height, energy, milk/fermentedmilk, cheese, alcohol, fruitveg_gram, meat, juicesoda_gram, totalfat, satfat, tobac, supplement, cci_weighted, cvd_baseline, diabetes_baseline`.

Notis: Vissa av dessa variabler är inte identiska mellan de två kohorterna. Exempelvis är enheten för variabeln `fruitveg_gram` inte gram för SMCC, eller att `tobac` har fyra kategorier i SMCC och tre kategorier i COSMC. Detta gör att vi inte bör slå ihop dessa data och dessutom vara försiktiga vid tolkning av dessa kovariat.

Analyser
1) Descriptives
2) Imputerat data.
   a. Börja med SMCC, justering A & B. För både mjölk och filmjölk, kör multipel linjär regression på samtliga protein i de tre panelerna. Använd FDR för respektive mjölk och filmjölk. Spara ned de som är dubbelt FDR-signifikanta (justering A & B) för åtminstone en av mjölk/fil.
   b. Utgå från listan efter a. och kör motsvarande analyser i COSMC (total). Spara ned de som är nominellt signifikanta ($p<0.05$) i åtminstone en av mjölk/fil.
   c. Från listan efter b., skapa två forest plots:
      En med mjölk-estimat (SMCC & COSMC)
      En med fil-estimat (SMCC & COSMC)
      I dessa grafer ska både justering A och B vara med
   d. Från listan efter b., kör analyser med splines. Presentera spline-grafer med såväl mjölk som filmjölk som exponering. Gör detta för båda kohorterna och ALL, Female och Male. Båda justeringarna A & B.

    e. Från listan efter a., kör linjära analyser för COSMC-kvinnor samt COSMC-män. Presenteras med tabell och forest plots. Både mjölk och filmjölk som exponering. Båda justeringarna A & B.
3) Som 2) fast för complete case.
4) Skapa linjär regression med mjölk/filmjölk som exponering och `kolesterol_ny`, `hdl_kolesterol_ny`, `ldl_kolesterol_ny` samt `crp` som utfall. Splinekurvor. Justering B.
5) Skapa splinekurvor med 6 grafer per protein, för de 5 protein som Kalle skickade mailledes 20230816. Kapa x-axeln vid 3. I övrigt följ Kalles instruktioner i mailet. Ska bli 5 sidor, med 6 kombinerade grafer i vardera. Alltså, egentligen bara en mer städad och avskalad variant av 2d.
